# Tumor Intrinsic METTL5 Modulates ATF4 Translation to Prevent T Cell‐Induced Ferroptosis in Ovarian Cancer

**DOI:** 10.1002/advs.202507718

**Published:** 2025-10-03

**Authors:** Jiakai Hou, Cheng‐Wei Ju, Nicholas A. Egan, Yanjun Wei, Yunfei Wang, Minghao Dang, Tianyi Zhou, Leilei Shi, Ningbo Zheng, Si Chen, Ashley M. Guerrero, Xiaofang Liang, Wanfu Wu, Areej Akhtar, Chitra Dhiman, Debanwita Roy Burman, Andro E. Gerges, Mason D. Flores, Han Li, Li‐Sheng Zhang, Marleen Kok, Xiaobo Mao, Linghua Wang, Qin Feng, Yiwen Chen, Sanghoon Lee, Daniel J. McGrail, Nidhi Sahni, Chuan He, Amir A. Jazaeri, Weiyi Peng

**Affiliations:** ^1^ Department of Biology and Biochemistry University of Houston Houston TX 77204 USA; ^2^ Department of Chemistry The University of Chicago Chicago IL 60637 USA; ^3^ Howard Hughes Medical Institute The University of Chicago Chicago IL 60637 USA; ^4^ Pritzker School of Molecular Engineering The University of Chicago Chicago IL 60637 USA; ^5^ Department of Bioinformatics and Computational Biology The University of Texas MD Anderson Cancer Center Houston TX 77030 USA; ^6^ Clinical Science Lab H. Lee Moffitt Cancer Center & Research Institute Tampa FL 33612 USA; ^7^ Department of Lymphoma and Myeloma The University of Texas MD Anderson Cancer Center Houston TX 77030 USA; ^8^ Department of Epigenetics and Molecular Carcinogenesis The University of Texas MD Anderson Cancer Center Houston TX 77030 USA; ^9^ Division of Tumor Biology & Immunology The Netherlands Cancer Institute Amsterdam 1066 CX The Netherlands; ^10^ Department of Medical Oncology The Netherlands Cancer Institute Amsterdam 1066 CX The Netherlands; ^11^ Neuroregeneration and Stem Cell Programs Institute for Cell Engineering Department of Neurology Johns Hopkins University School of Medicine Baltimore MD 21218 USA; ^12^ Department of Genomic Medicine The University of Texas MD Anderson Cancer Center Houston TX 77030 USA; ^13^ Quantitative Sciences Program The University of Texas MD Anderson Cancer Center UT Health Graduate School of Biomedical Sciences Houston TX USA; ^14^ Department of Gynecologic Oncology and Reproductive Medicine Division of Surgery The University of Texas MD Anderson Cancer Center Houston TX 77030 USA; ^15^ Center for Immunotherapy and Precision Immuno‐Oncology Cleveland Clinic Cleveland OH 44195 USA; ^16^ Department of Biochemistry and Molecular Biology The University of Chicago Chicago IL 60637 USA; ^17^ Institute for Biophysical Dynamics The University of Chicago Chicago IL 60637 USA

**Keywords:** ATF4, CRISPR screen, ferroptosis, immunotherapy, METTL5, ovarian cancer

## Abstract

Poor clinical responses to immune checkpoint blockade (ICB) observed in ovarian cancer (OC) highlight an unmet need to understand the mechanisms driving immune evasion in this disease. To address this, an integrative analysis is conducted by combining in vitro genome‐wide immune screens, in vivo ICB screens, and clinical data mining, and METTL5 is identified as a crucial OC‐intrinsic factor that promotes immune resistance. Immunologically “cold” OC tumors and poor responders to ICB exhibit elevated *METTL5* expression. Mechanistically, knocking out (KO) *METTL5* in OC disrupts ATF4 translation by altering 18S rRNA m^6^A levels, leading to the downregulation of *SLC7A11* and *SLC3A2*, whose function is to suppress ferroptosis activity. Consequently, *METTL5* KO enhances tumor sensitivity to T cell‐mediated antitumor immunity. Notably, the immune‐sensitive phenotypes seen in *METTL5*‐KO tumors can be reversed by either *ATF4* overexpression or ferroptosis inhibition. These findings underscore the central role of the METTL5/ATF4/ferroptosis axis in controlling OC responses to immunotherapy.

## Graphical abstract

An integrative analysis combining in vitro genome‐wide immune screens, in vivo ICB screens, and clinical data mining identifies METTL5 as a key tumor‐intrinsic immune factor. Mechanistic studies further reveal the importance of the METTL5‐ATF4‐SLC7A11/SLC3A2‐ferroptosis axis in modulating antitumor immune responses. These findings highlight METTL5 as a prognostic biomarker and therapeutic target in ovarian cancer.

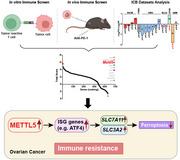

## 1 Introduction

Despite recent achievements in immune checkpoint blockade (ICB), only 5–15% of patients with ovarian cancer (OC) respond to single‐agent ICB treatment in part due to immunosuppressive mechanisms exploited by tumor cells and cancer‐educated stromal cells.^[^ ^1^, ^2^, ^3^ ^]^ This highlights an urgent need to identify OC‐associated immune evasion mechanisms and develop novel strategies to overcome primary resistance to immunotherapy in patients with OC. Our group, along with others, has previously developed high‐throughput genetic screening platforms to investigate the immunosuppressive roles of tumor intrinsic factors using paired tumors and tumor‐reactive T cells.^[^ ^4^, ^5^, ^6^, ^7^, ^8^, ^9^ ^]^ Using these platforms, we discovered that hyperactivation of oncogenic or metabolic pathways makes tumors resistant to T‐cell killing. Consequently, inhibiting these tumor intrinsic pathways has been reported to enhance the efficacy of immunotherapy for melanoma.^[^ ^4^, ^5^, ^10^ ^]^ These preclinical findings have guided the development of effective and safe immuno‐oncology (IO) combinations to overcome immune resistance in melanoma patients.^[^ ^11^ ^]^

However, recent clinical results from the OC‐ICB trials, including JAVELIN‐200,^[^ ^12^ ^]^ IMAGYN‐50,^[^ ^13^ ^]^ and KEYNOTE‐100,^[^ ^2^ ^]^ suggest that OC exhibits distinctive immunobiology, making it challenging to directly extrapolate immune‐related findings from melanoma to OC. For example, OC is comprised of various histopathological subsets with high heterogeneity, each exhibiting distinct molecular features.^[^ ^14^ ^]^ Among them, high‐grade serous ovarian carcinoma (HGSOC) is the most common histology, accounting for around 70% of cases. More than 96% of HGSOC patients carry pathogenic *TP53* mutations.^[^ ^15^ ^]^ In addition to well‐established roles in controlling cell‐cycle arrest, apoptosis, and DNA damage repair, *TP53* mutations have been reported to impair cGAS/STING activation, downregulate MHC class I expression, suppress antigen presentation in tumor cells, and increase circulating immunosuppressive neutrophils.^[^ ^16^, ^17^, ^18^ ^]^ These pathways controlled by *TP53* mutations exert suppressive effects on both the innate and adaptive immune systems, promoting an immunologically “cold” tumor microenvironment in OC. Moreover, unlike melanoma, which has a high tumor mutation burden (TMB; median> 10 mutations per megabase) due to UV exposure,^[^ ^19^ ^]^ HGSOC displays a low TMB (median <3 mutations per megabase). Even in *BRCA*‐mutated OC tumors with defective DNA repair machinery, TMBs largely remain below 10 mutations per megabase.^[^ ^1^, ^20^ ^]^ Low TMB in OC impedes immune recognition, particularly by T cells. Conversely, recent clinical data have shown that using a predetermined TMB cutoff of 10 mutations per megabase can distinguish OC patients who respond to pembrolizumab from those who do not.^[^ ^2^ ^]^ The distinctive immunobiology of OC underscores the importance of characterizing the landscape of OC‐intrinsic immune factors to develop future personalized therapeutic approaches for OC patients.

To better understand OC‐intrinsic mechanisms of immunotherapy resistance, we conducted an integrative analysis by combining in vitro genome‐wide immune screens, in vivo ICB screens, and clinical data mining in the context of OC. Our integrative analyses identified METTL5 as an OC‐intrinsic factor that promotes immune resistance. METTL5, a member of the methyltransferase‐like (METTL) family, exhibits methyltransferase activity, transferring methyl groups from S‐adenosyl methionine (SAM) to the nucleotide A‐1832 (A1832) of 18S ribosomal RNA (rRNA). The A1832 methylation of 18S rRNA plays a critical role in neural development and lipid metabolism by regulating translation efficiency.^[^ ^21^ ^]^ Loss‐of‐function mutations or deletion of *METTL5* in humans and mice lead to developmental abnormalities.^[^ ^22^, ^23^ ^]^ Additionally, increased METTL5 activity is associated with the development of hepatocellular carcinoma.^[^ ^24^ ^]^ However, the immunological role of METTL5 remains largely unexplored.

Here, we confirmed a negative correlation between *METTL5* expression and the clinical outcomes following immunotherapy in HGSOC patients. Mechanistically, our multiomic analyses demonstrated that knocking out *METTL5* reduces the translation efficiency of ATF4 by altering N6‐methyladenosine (m^6^A) methylation of 18S rRNA. Reduced ATF4 translation in *METTL5*‐deficient OC cells enhances tumor ferroptosis. Since ferroptosis was recently identified as a T cell‐mediated antitumor mechanism,^[^ ^25^, ^26^ ^]^ *METTL5* knockout ultimately increases tumor sensitivity to T cell‐mediated immune responses. Overexpressing *ATF4* can reverse the immune‐sensitive phenotype of *METTL5*‐deficient OC cells both in vitro and in vivo. Collectively, this work provides novel insights regarding the landscape of OC‐intrinsic immune regulators and reveals the potential of METTL5 as a promising therapeutic target to overcome immune resistance in OC patients.

## 2 Results

### 2.1 In Vitro Genome‐Wide Immune Screens and Clinical Correlation Analysis are Used to Guide the Establishment of ICB Sublibraries for In Vivo Immune Screens

**Figure 1:**
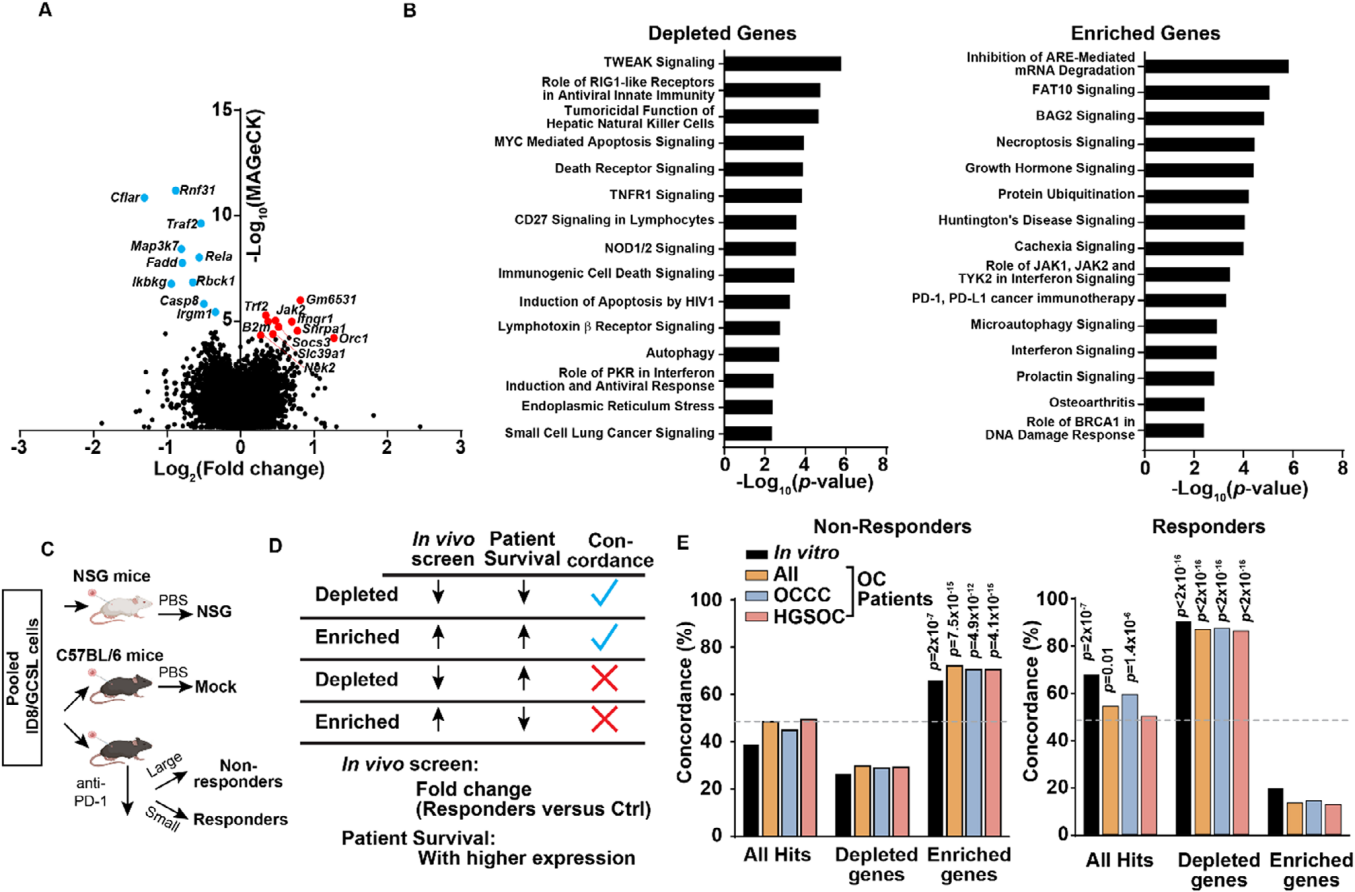
CRISPR immune screens identify ovarian cancer (OC)‐intrinsic factors controlling responses to immune checkpoint blockade (ICB) therapy. A) A volcano plot illustrating top candidates of tumor‐intrinsic factors that are either depleted or enriched in in vitro CRISPR immune screens at an effector‐to‐target (ET) ratio of 0.2:1. Gene‐level MAGeCK scores and changes in gRNA distribution between ID8/GC cells treated with or without T cells were calculated. The Log_2_ fold change of the second‐best gRNA for each gene was selected for data representation. The top 10 genes with significantly enriched or depleted gRNAs in the T‐cell‐treated group (|Log_2_(Fold change)| > 0.25, *p* < 0.05) were highlighted as red and blue dots, respectively. *p*‐values were calculated using a negative binomial model, and two one‐sided *p*‐values assessed whether gRNAs were positively or negatively selected. B) Ingenuity Pathway Analysis (IPA) of identified tumor‐intrinsic factors mediating T‐cell killing. The top 15 pathways associated with depleted genes (left panel) and enriched genes (right panel) with statistical significance (*p* < 0.05) were shown. C) A schematic diagram of in vivo CRISPR immune screens. Pooled gRNA‐expressing ID8/GCSL cells were subcutaneously inoculated into either NSG mice or C57BL/6 mice at 1x10^7^ cells per mouse. Five days post‐inoculation, C57BL/6 mice were randomized into PBS (Mock) or anti‐PD‐1 treatment groups. Tumors were harvested after 6 courses of corresponding treatments (every two days). In the anti‐PD‐1 treatment group, mice were categorized as responders or nonresponders based on the median tumor size at the time of tumor collection. D) List of criteria for concordance analysis of data obtained from in vivo CRISPR immune screens and transcriptomic data of baseline tumor samples from the phase II trial (NCT03026062) of durvalumab and tremelimumab treatments in recurrent or refractory ovarian cancer at MD Anderson Cancer Center. E) Concordance rates between the performance of gRNAs in each category of in vivo immune screens and the corresponding results of in vitro immune screens or patient data analysis. Results from the nonresponder group (left) and the responder group (right) of in vivo immune screens were plotted. Exact *p*‐values for statistically significant findings were noted.

To create a gRNA library for in vivo screens of OC‐associated immune regulators, we first performed a genome‐wide immune screen to determine which candidates can modulate tumor sensitivity to in vitro T cell‐mediated killing, as previously described.^[^ ^5^ ^]^ In the in vitro OC screen, we used a Cas9 and gp100‐expressing ID8 cell line (ID8/GC), which can be recognized by gp100‐specific T cells derived from Pmel mice (Figure S1A, Supporting Information). The in vitro OC screen conditions were optimized to achieve a 50‐80% T cell killing rate (Figure S1B). Based on these results, we co‐cultured pooled gRNA library‐expressing ID8/GC cells with Pmel T cells at effector/target (ET) ratios of 0.2:1 and 1:1, resulting in 64% and 82% cytotoxicity, respectively (Figure S1C, Supporting Information). The abundance of each type of gRNA‐expressing cells in surviving tumor cells was evaluated by next‐generation sequencing. Our results showed that gRNA distributions among replicates were highly correlated (Figure S1D, Supporting Information). Depletion of genes essential for proliferation and survival was observed in tumor samples after 7‐day expansion, confirming the success of gRNA‐based gene editing (Figure S1E, Supporting Information). By comparing with tumor samples without T cell treatment, we identified a set of depleted and enriched candidates in T cell‐treated tumor samples, with 71 depleted and 75 enriched candidates appearing in treated tumor samples at both ET ratios (**Figure** **1**A, S1F,G, Supporting Information Tables S1 and S2, Supporting Information). Multiple signaling pathways with known immune functions were enriched among the candidates from the ET ratio of 0.2:1 group, such as the immunological cell death signaling pathway for depleted genes and the interferon signaling pathway for enriched genes (Figure 1B), providing confirmation of validity for candidates identified in this in vitro OC screen.

To increase the clinical relevance of selected candidates, we integrated in vitro OC screen results with the results from clinical correlation analysis, and calculated in vitro scores and patient scores for individual genes as described in Figure S2 (Supporting Information). The differences in gene expression between ICB responders and nonresponders in 16 published ICB‐cancer cohorts^[^ ^27^, ^28^, ^29^, ^30^, ^31^, ^32^, ^33^, ^34^, ^35^, ^36^, ^37^, ^38^, ^39^, ^40^, ^41^ ^]^ were used to calculate patient scores (Table S3, Supporting Information). The merged scores were further generated by adding in vitro scores and paired patient scores. Genes with the lowest 400 merged scores (depleted genes) and the highest 100 merged scores (enriched genes) were selected to generate gene‐specific gRNAs for the construction of the ICB sub‐library (Figure S3A and Table S4, Supporting Information). Additionally, the ICB sub‐library includes gRNAs targeting 125 depleted genes and 38 enriched genes identified by our previous in vitro immune screens.^[^ ^5^ ^]^ Besides gRNAs used for functional interrogation, gRNAs targeting essential genes that control cell proliferation and survival^[^ ^42^ ^]^ serve as positive controls to evaluate the success of genetic perturbation, and nontargetable gRNAs are included as negative controls. A total of 3527 gRNAs from four categories based on their targets were included in the ICB sub‐library for in vivo immune screens (Figure S3B, Supporting Information).

### 2.2 In Vivo Immune Screen Results Show a High Concordance with Clinical Outcome of ICB‐Treated OC Patients

We transduced ID8/GC cells with the ICB sub‐library, selected gRNA‐transduced ID8/GC cells (ID8/GCSL) using puromycin, and expanded ID8/GCSL cells in vitro for 7 days. We observed that a proportion of gRNAs in the essential group, but not in the rest three groups (others), were depleted in pooled ID8/GCSL cells after 7 days of in vitro expansion (Figure S3C, Supporting Information**),** suggesting a successful gene‐specific perturbation. Next, pooled ID8/GCSL cells were subcutaneously inoculated into either immunodeficient mice (NSG) or immunocompetent mice (C57BL/6). Tumor‐bearing mice were treated with either PBS (the Mock group) or anti‐PD‐1 (Figure 1C). C57BL/6 mice receiving anti‐PD‐1 treatment were stratified into nonresponders and responders according to whether their tumor sizes were above or below the median value, respectively. Mice in the responder group exhibited smaller tumor sizes than those in the mock group (Figure S3D, Supporting Information). When we analyzed the gRNA distribution patterns in different groups, we consistently observed dramatic depletion of gRNAs of essential targets in all groups of mice when compared with negative control gRNAs (Figure S3E, Supporting Information). The depletion of gRNAs targeting genes‐of‐interest (GOIs) was only shown in the responder group (Figure S3E, Supporting Information). Given that 74% of gRNAs in the ICB sub‐library target candidates contributing to ICB resistance, and those gRNAs are expected to be depleted in tumors responding to ICB treatment, the in vivo ICB screen results align well with our expectations.

Next, we evaluated whether the performance of each candidate in the in vivo ICB screen is consistent with the association between gene expression and ICB response. We characterized transcriptome profiles of pretreatment tumor samples from OC patients, including both HGSOC and clear cell OC (OCCC), enrolled in the phase II trial (NCT03026062) of durvalumab and tremelimumab at MD Anderson Cancer Center (MDACC‐OC cohort). Then, we determined the association between gene expression and patient survival in this cohort. The concordance between the in vivo ICB screen and the in vitro genome‐wide immune screen or patient survival was defined as described in Figure 1D. The concordance percentages between in vitro and in vivo screens serve as the maximal expected values. When compared with ICB‐treated OC patient survival, high concordance percentages of depleted hits (immune resistant) and enriched hits (immune sensitizing) were achieved in the responder group and the nonresponder group, respectively (Figure 1E). Furthermore, the concordance percentages between in vivo screens and patient survival are largely comparable in HGSOC and OCCC patients (Figure 1E). Collectively, our in vivo ICB screen and translational correlation identify a set of OC‐intrinsic immune factors with the potential to control antitumor immune responses.

### 2.3 METTL5 is a Key OC‐Intrinsic Factor Contributing to Immune Resistance

**Figure 2:**
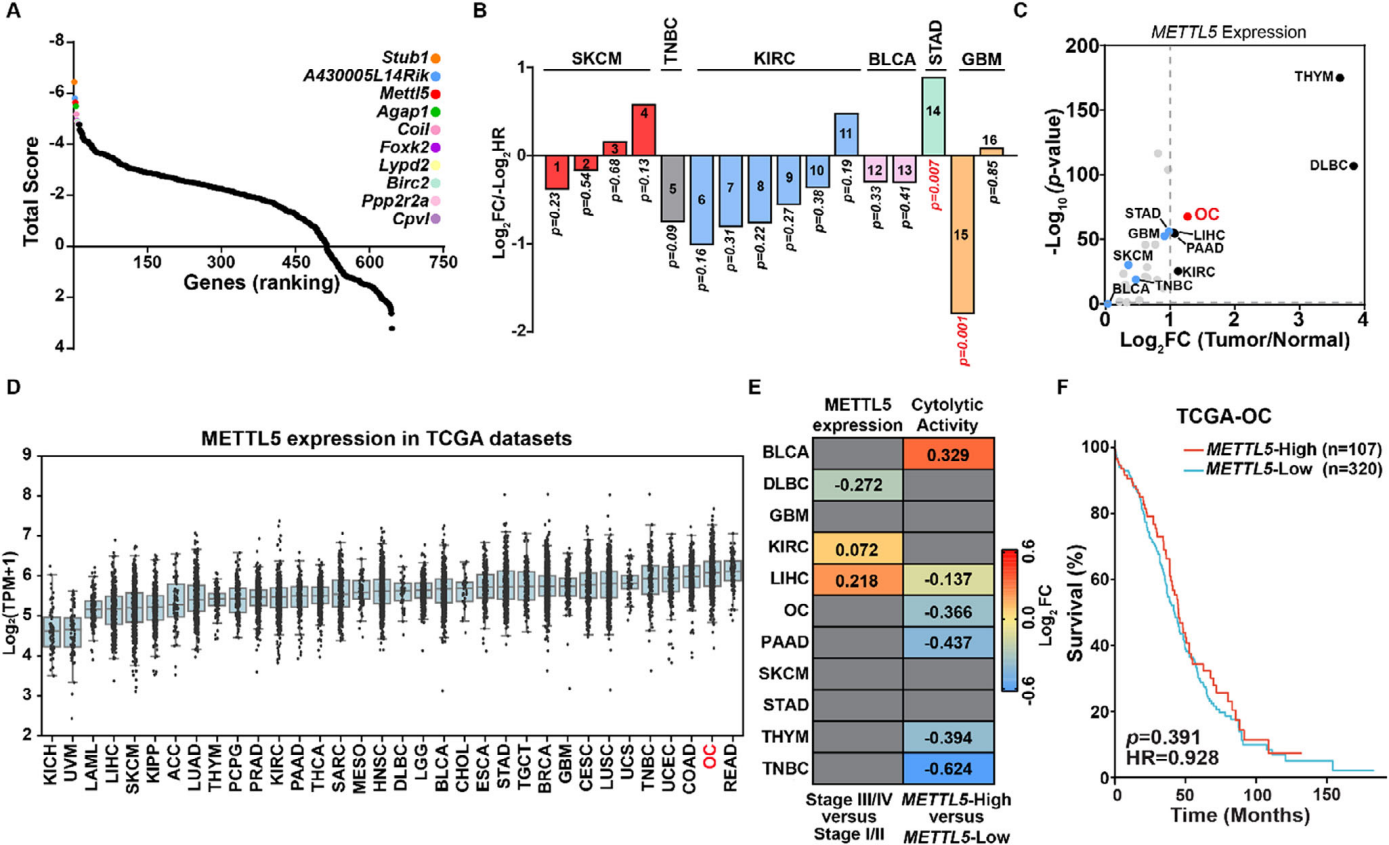
METTL5 is identified as one of the key OC‐intrinsic factors modulating T cell‐mediated antitumor immunity. A) Functional evaluation of OC‐intrinsic immune factor candidates. Total scores of each candidate were calculated by integrating results from in vivo CRISPR immune screens and computed patient scores from 16 publicly available ICB‐treated cohorts. Negative total scores indicate corresponding genes potentially promoting immune resistance (depleted genes). The top 10 genes with the lowest total scores are highlighted. B) Analysis of *METTL5* expression levels in cancer patients receiving ICB therapy, comparing those who responded to treatment with those who did not. The mRNA levels of *METTL5* in tumors were extracted from previously reported datasets. In cohort #15, patients were stratified by *METTL5* expression, and the Log_2_ Hazard Ratio (HR) for *METTL5*‐high was calculated. In the other cohorts, patients were categorized as responders and nonresponders based on clinical outcomes in response to ICB. Log_2_FC in *METTL5* expression between responders and nonresponders, along with corresponding *p‐*values was determined. Statistically significant *p*‐values are marked in red. C) Comparison of *METTL5* expression levels in tumor versus normal tissues. The mRNA levels of *METTL5* in tumors and normal tissues were extracted from publicly available TCGA and GTEx datasets, respectively. FCs of (Tumor versus Normal tissues) and associated *p*‐values were calculated for each cancer type. Cancer types (except OC) with significantly upregulated *METTL5* (Log_2_FC> 1 and *p <* 0.05) are represented by black dots, while cancer types with available ICB‐treated patient datasets are labeled with blue dots, and OC is labeled with a red dot. D) Comparison of *METTL5* expression levels across different cancer types. The mRNA levels of *METTL5* in tumors were extracted from publicly available TCGA datasets. A boxplot of *METTL5* expression across various tumor types was illustrated by Log_2_ (TPM+1). E) Associations between *METTL5* expression with cancer stage and cytolytic activity. Patients enrolled in TCGA datasets were grouped by cancer stage (III/IV versus I/II) or METTL5 expression (high versus low). Cytolytic activity scores were calculated for *METTL5*‐high and *METTL5*‐low groups. Log_2_FC values that reached statistical significance were visualized in a heatmap, while nonsignificant comparisons were indicated in gray. F) Kaplan–Maier curves comparing overall survival of *METTL5*‐High and *METTL5*‐Low OC patients. Abbreviations: ACC, Adrenocortical carcinoma; BLCA, Bladder urothelial carcinoma; BRCA, Breast invasive carcinoma; CESC, Cervical and endocervical cancers; CHOL, Cholangiocarcinoma; COAD, Colon adenocarcinoma; DLBC, Lymphoid Neoplasm Diffuse Large B‐cell Lymphoma; ESCA, Esophageal carcinoma; GBM, Glioblastoma multiforme; HNSC, Head and Neck squamous cell carcinoma; KICH, Kidney Chromophobe; KIRC, Kidney renal clear cell carcinoma; KIRP, Kidney renal papillary cell carcinoma; LAML, Acute Myeloid Leukemia; LGG, Brain Lower Grade Glioma; LIHC, Liver hepatocellular carcinoma; LUAD, Lung adenocarcinoma; LUSC, Lung squamous cell carcinoma; MESO, Mesothelioma; OC, Ovarian serous cystadenocarcinoma; PAAD, Pancreatic adenocarcinoma; PCPG, Pheochromocytoma and Paraganglioma; PRAD, Prostate adenocarcinoma; READ, Rectum adenocarcinoma; SARC, Sarcoma; SKCM, Skin Cutaneous Melanoma; STAD, Stomach adenocarcinoma; TGCT, Testicular Germ Cell Tumors; THCA, Thyroid carcinoma; THYM, Thymoma; TNBC, Triple negative breast cancer; UCEC, Uterine Corpus Endometrial Carcinoma; UCS, Uterine Carcinosarcoma; UVM, Uveal Melanoma.

To better rank identified immunotherapy modulating candidates, we calculated a total score for each target and ranked them accordingly. Interestingly, we re‐discovered *STUB1*, which encodes an E3 ubiquitin ligase, as a key immune regulator with the lowest total score (**Figure** **2**A and Table S5, Supporting Information). Recent research has shown that increased *STUB1* expression can facilitate cancer immune evasion and lead to resistance against immunotherapy.^[^ ^43^, ^44^ ^]^ Given that the role of *METTL5*, the third highest‐ranking gene, in antitumor immunity remains largely unexplored, we selected it for further studies (Figure 2A). Eleven out of sixteen published ICB cohorts showed downregulated *METTL5* expression in patients with clinical benefit, with statistically significant downregulation observed in one cohort of glioblastoma patients (Figure 2B). Furthermore, upregulated *METTL5* expressions were observed in OC, large B‐cell Lymphoma (DLBC), thymoma (THYM), liver hepatocellular carcinoma (LIHC), pancreatic adenocarcinoma (PAAD), and kidney renal clear cell carcinoma (KIRC) compared to corresponding normal tissues (Figure 2C). Pan‐cancer TCGA analysis revealed that OC exhibits the second‐highest *METTL5* expression among 34 types of cancers (Figure 2D). *METTL5* expression levels in stage III/IV tumors, including OC, are largely comparable with those in stage I/II tumors (Figure 2E and Figure S4A, Supporting Information**)**. In OC patients, there is no significant difference in overall survival between patients with high *METTL5* expression and those with low *METTL5* expression (Figure 2F **)**. Given that the majority of patients in the pan‐cancer TCGA datasets have not received immunotherapy, our data suggest that upregulated *METTL5* expression does not impact the prognosis of OC patients in the absence of immunotherapy.

However, tumors with high *METTL5* expression exhibit reduced cytolytic activity score (an indicator of the magnitude of antitumor immune activity) in OC, LIHC, PAAD, THYM, and triple‐negative breast cancer (TNBC) (Figure 2E and Figure S4B, Supporting Information).

**Figure 3:**
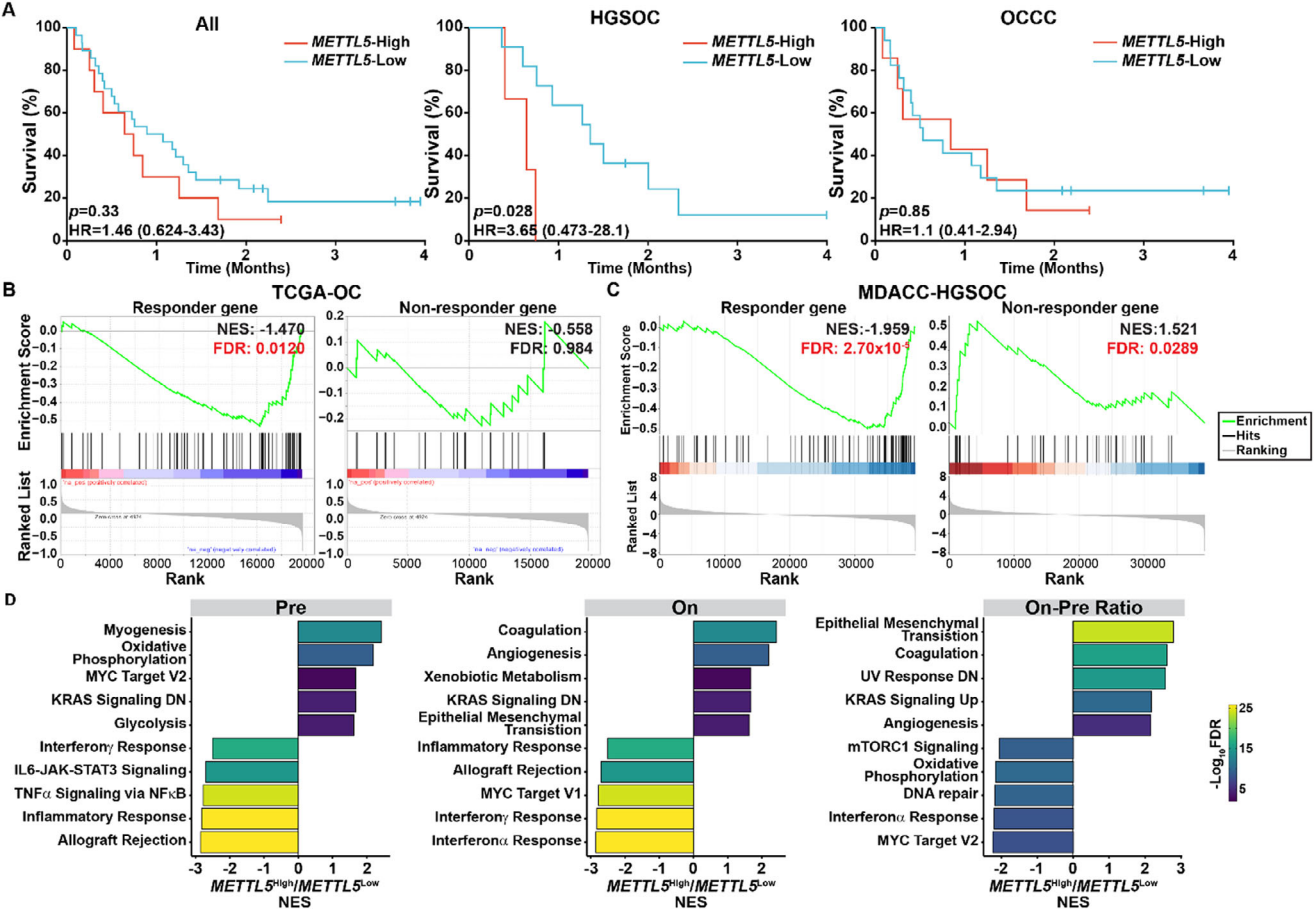
Increased *METTL5* expression correlates with poor responses to ICB treatment in OC patients. A) Correlation between *METTL5* expression and overall survival in OC patients treated with ICB. Transcriptomic and clinical data from the MDACC‐OC cohort were analyzed. Patients were stratified in *METTL5*‐High and *METTL5*‐Low groups based on the upper quartile values of *METTL5* in baseline tumor samples. Kaplan‐Meier survival curves were plotted for all patients, as well as subgroups with high‐grade serous ovarian cancer (HGSOC) and ovarian clear cell carcinoma (OCCC). Hazard ratios (HRs) for *METTL5* upregulation and their associated *p*‐values were reported. B,C) Gene set enrichment analysis (GSEA) of ICB response‐associated gene sets in OC samples with differential *METTL5* expressions. Transcriptomic data from baseline triple‐negative breast cancer (TNBC) tumors in the TONIC cohort were used to identify differentially expressed genes (DEGs) between responders and nonresponders. The top 100 upregulated genes in responders and nonresponders formed the responder and nonresponder gene sets, respectively. Patients in the TCGA‐OC cohort B) and the MDACC‐HGSOC cohort C) were stratified into *METTL5*‐High and *METTL5*‐Low groups based on *METTL5* expression in baseline tumor samples. Gene expression differences between these groups were analyzed, and GSEA was conducted to assess the association of *METTL5* upregulation with poor ICB responses. Normalized enrichment scores (NES) and false discovery rates (FDR) were calculated, with statistically significant FDR values (FDR < 0.05) highlighted in red. D) Transcriptomic changes associated with *METTL5* expression during the course of ICB treatment. Baseline tumor samples (“Pre”) and on‐treatment samples (“On”) were collected from patients in the MDACC‐HGSOC cohort prior to treatment and immediately before the initiation of the third treatment cycle, respectively. Patients were stratified into *METTL5*‐High and *METTL5*‐Low groups based on *METTL5* expression in baseline tumors. DEGs were identified based on fold changes (|Log_2_FC| > 1) and adjusted *p*‐values (< 0.25). These DEGs were used for GSEA to identify hallmark pathways associated with *METTL5* expression. The top five significantly enriched pathways (FDR < 0.01) in each direction were reported, based on NES values.

More importantly, we stratified patients in the MDACC‐OC cohort based on *METTL5* expression in baseline (pre‐ICB) tumor samples, and compared the overall survival between *METTL5*‐High and *METTL5*‐Low patients. Among ICB‐treated HGSOC patients, *METTL5*‐High patients showed significantly shorter survival time when compared to *METTL5*‐Low patients (**Figure** **3**A). In contrast, there was no significant difference in overall survival among ICB‐treated OCCC patients (Figure 3A). Our data, combined with the analysis of published ICB cohorts, suggests that the correlation between high *METTL5* expression and an immune‐resistant phenotype varies across different cancer types. Notably, upregulation of *METTL5* is prominent in OC compared to most other cancer types, and high *METTL5* expression is significantly associated with immunotherapy resistance in HGSOC. Then, we investigated the association between *METTL5* expression and molecular characteristics of tumors responding to ICB. Considering that the infiltration of immune cells within tumors is linked to immunotherapy outcomes, we examined the relationship between *METTL5* expression and key immune cell markers in tumor samples from both datasets, *METTL5* expression shows no significant association with the expressions of key immune cell markers for T cell, cytotoxic T cell (CTL), B cell and NK cells at tumor sites (Figure S4C, Supporting Information). To further investigate the contribution of tumor‐intrinsic METTL5 to immune resistance beyond regulation of immune cell recruitment, we utilized data from a published ICB‐cohort^[^ ^30^ ^]^ from TNBC tumors, which are similar to OC at the molecular level.^[^ ^45^ ^]^ Upregulated differentially expressed genes (DEGs) identified in ICB responders and nonresponders in the TNBC cohort were used to define the responder gene set and the nonresponder gene set, respectively (Table S6, Supporting Information). The changes in transcriptome profiles between *METTL5*‐High and *METTL5*‐Low HGSOC tumors in two independent cohorts (TCGA‐OC and MDACC‐HGSOC) were analyzed by Gene Set Enrichment Analysis (GSEA) using the responder and nonresponder gene sets. In both cohorts, upregulated DEGs in *METTL5*‐High tumors show a significantly negative enrichment in the responder gene set, suggesting that METTL5 may downregulate expression of transcripts associated with ICB response (Figure 3B,C). Consistently, GSEA using the canonical pathway gene sets revealed that multiple immunostimulatory pathways, such as those related to IFN/TNF responses and inflammatory responses, were significantly enriched in downregulated DEGs in *METTL5*‐High, pretreatment tumors in the MDACC‐HGSOC cohort (Figure 3D). Interestingly, the expression levels of IFN‐related genes in on‐treatment samples and their ratios of on‐treatment and pretreatment remain lower in *METTL5*‐High tumors than in *METTL5*‐Low tumors (Figure 3D and Figure S4D, Supporting Information). Given that impaired IFN pathways have been implicated in predicting poor ICB clinical response,^[^ ^46^, ^47^ ^]^ these results provide additional clinical evidence to support METTL5 acts as an OC‐intrinsic immunosuppressive factor.

### 2.4 METTL5 Contributes to Immune Resistance by Regulating the Ferroptosis Pathway

**Figure 4:**
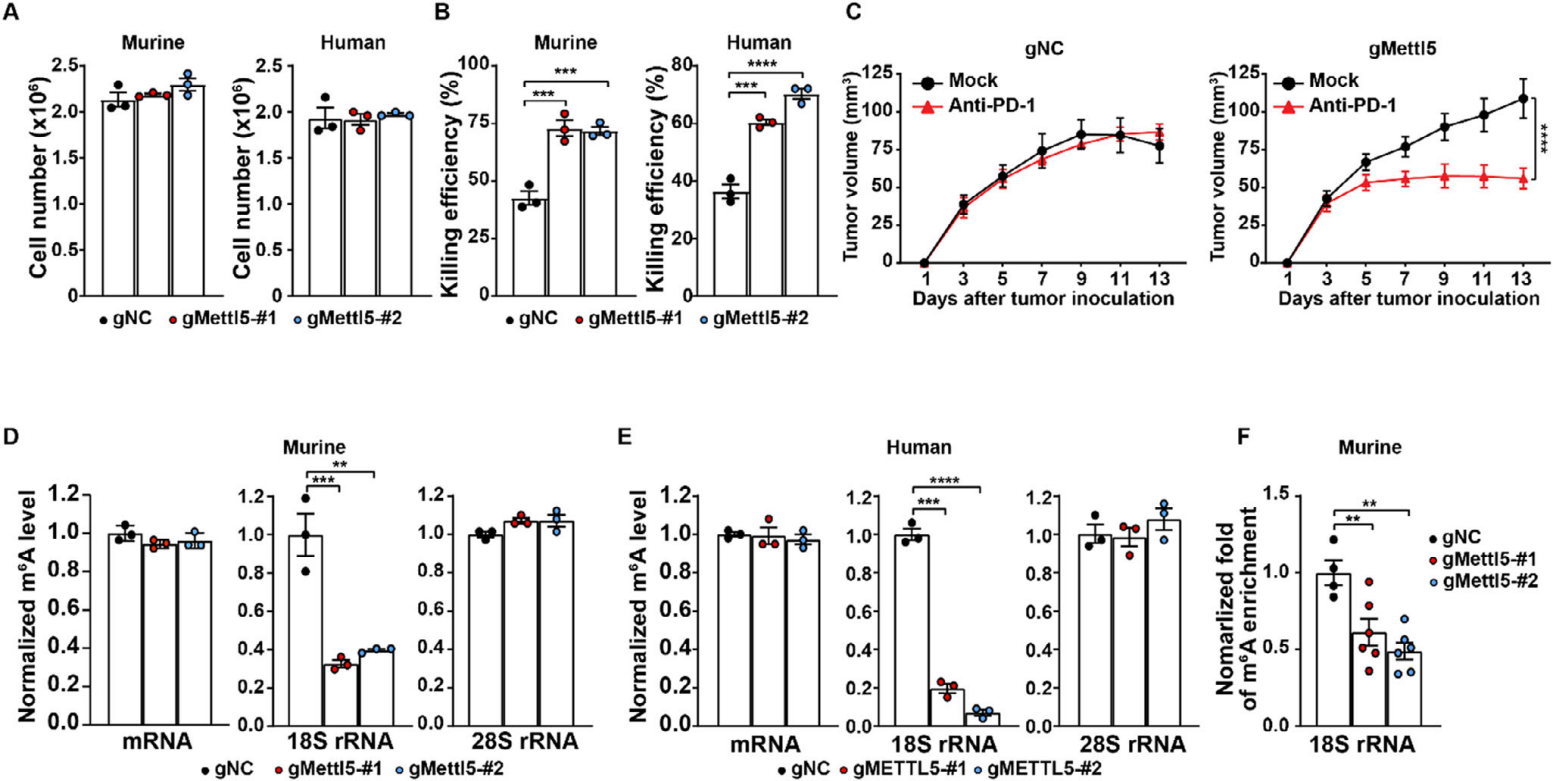
*METTL5* inhibition enhances T cell‐mediated killing and reduces m^6^A methylation of rRNAs in ovarian cancer cells. A) in vitro growth of OC cells with genetic depletion of *METTL5*. Murine OC (ID8/GC) and human OC (SKVO3/C) lines were genetically modified to express either nontargetable gRNAs (gNC) or *METTL5*‐specific gRNAs (gMettl5). Equal numbers of genetically modified OC cells were seeded, and cell numbers were counted after 48‐h of in vitro culture. Data were analyzed using one‐way ANOVA followed by Dunnett's post hoc test and were presented as mean values ± SEM (*n* = 3). B) Sensitivity of gMettl5 tumors to T cell‐mediated killing. Equal numbers of murine and human OC cells were co‐cultured overnight with tumor‐reactive Pmel T cells or hB7H3‐CAR‐T cells, respectively. The numbers of live tumor cells were counted, and killing efficiency was calculated. Relative changes in cell numbers were normalized to the groups without T cell treatment. Data were analyzed using one‐way ANOVA followed by Dunnett's post hoc test and were presented as mean values ± SEM (*n* = 3). C) Enhanced in vivo efficacy of anti‐PD‐1 treatment with *METTL5* genetic depletion. Modified ID8/GC cells were inoculated into C57BL/6 mice. Three days after tumor inoculation, mice with measurable tumors were randomized and treated intraperitoneally with either PBS or anti‐PD‐1 (100 µg per dose) every other day. The tumor size was measured every 2 days. Data were analyzed using two‐way ANOVA followed by the multiple comparison test and were presented as mean values ± SEM (*n* = 5). D,E) Reduction in m^6^A levels of 18S rRNA upon *METTL5* genetic depletion in murine ID8/GC cells D) and human SKVO3/C cells E). LC‐MS analysis quantified the levels of modified and unmodified adenosine in different RNA types. Data were analyzed using one‐way ANOVA followed by Dunnett's post hoc test and were presented as mean values ± SEM (*n* = 3). F) Enrichment of m^6^A‐modified 18S rRNAs in ID8/GC cells with or without *Mettl5* knockout. RNAs from gMettl5 and gNC cells were immunoprecipitated using an anti‐m^6^A antibody. Real‐time PCR was used to determine the levels of 18S rRNA. Enrichment was normalized to input samples. Data were analyzed using one‐way ANOVA followed by Dunnett's post hoc test and were presented as mean values ± SEM (*n* = 4 or 6). Representative data from at least two independent experiments were shown. **, *p* < 0.01; ***, *p* < 0.001; ****, *p* < 0.0001.

We sought to validate the immunosuppressive role of METTL5 and reveal its underlying mechanisms. We knocked out *METTL5* in human OC (Cas9‐expressing SKOV3) and murine OC (ID8/GC) cells (Figure S5A, Supporting Information), and found that *METTL5* knockout (KO) does not affect in vitro proliferation of OC cells (**Figure** **4**A). However, both *METTL5‐*KO human and murine tumor cells display increased susceptibility to T cell‐mediated killing when compared with corresponding control cells (Figure 4B). However, *Mettl5* KO in murine OC cells did not reduce IFNγ production by tumor‐reactive CD8⁺ T cells after tumor stimulation, suggesting that loss of *Mettl5* minimally affects immune synapse formation between CD8⁺ T cells and tumors (Figure S5B, Supporting Information). While the growth of ID8/GC tumors expressing *Mettl5*‐specific gRNA (gMettl5) in immunodeficient mice is comparable with control tumors (Figure S5C, Supporting Information), gMettl5 tumors are more sensitive to in vivo anti‐PD‐1 treatment in syngeneic murine models (Figure 4C). To determine whether the anti‐PD‐1 effect in gMettl5 tumors depends on CD4⁺ or CD8⁺ T cells, we selectively depleted one of T cell subsets before anti‐PD‐1 treatment as previously described.^[^ ^48^ ^]^ Our results showed that depletion of CD8⁺ T cells, but not CD4⁺ T cells, markedly diminished the antitumor efficacy of anti‐PD‐1 therapy in *Mettl5*‐KO tumors (Figure S5D–F, Supporting Information). However, infiltration of lymphocytes in *Mettl5*‐KO tumors is largely comparable with that in control tumors, regardless of receiving anti‐PD‐1 treatment (Figure S6, Supporting Information).

When we evaluated the effects of *METTL5* KO on m^6^A RNA levels by mass spectrometry, we found that the m^6^A methylation levels in 18S rRNA, but not in mRNA and 28S rRNA, were reduced in murine and human *METTL5‐*KO cells (Figure 4D,E). Furthermore, we performed real‐time PCR to analyze RNA samples immunoprecipitated by anti‐m^6^A antibody. We observed decreased methylation level of 18S rRNA in *METTL5‐*KO cells (Figure 4F). These results suggest that 18S rRNA m^6^A methylation is involved in the immunosuppressive role of METTL5 in OC.

**Figure 5:**
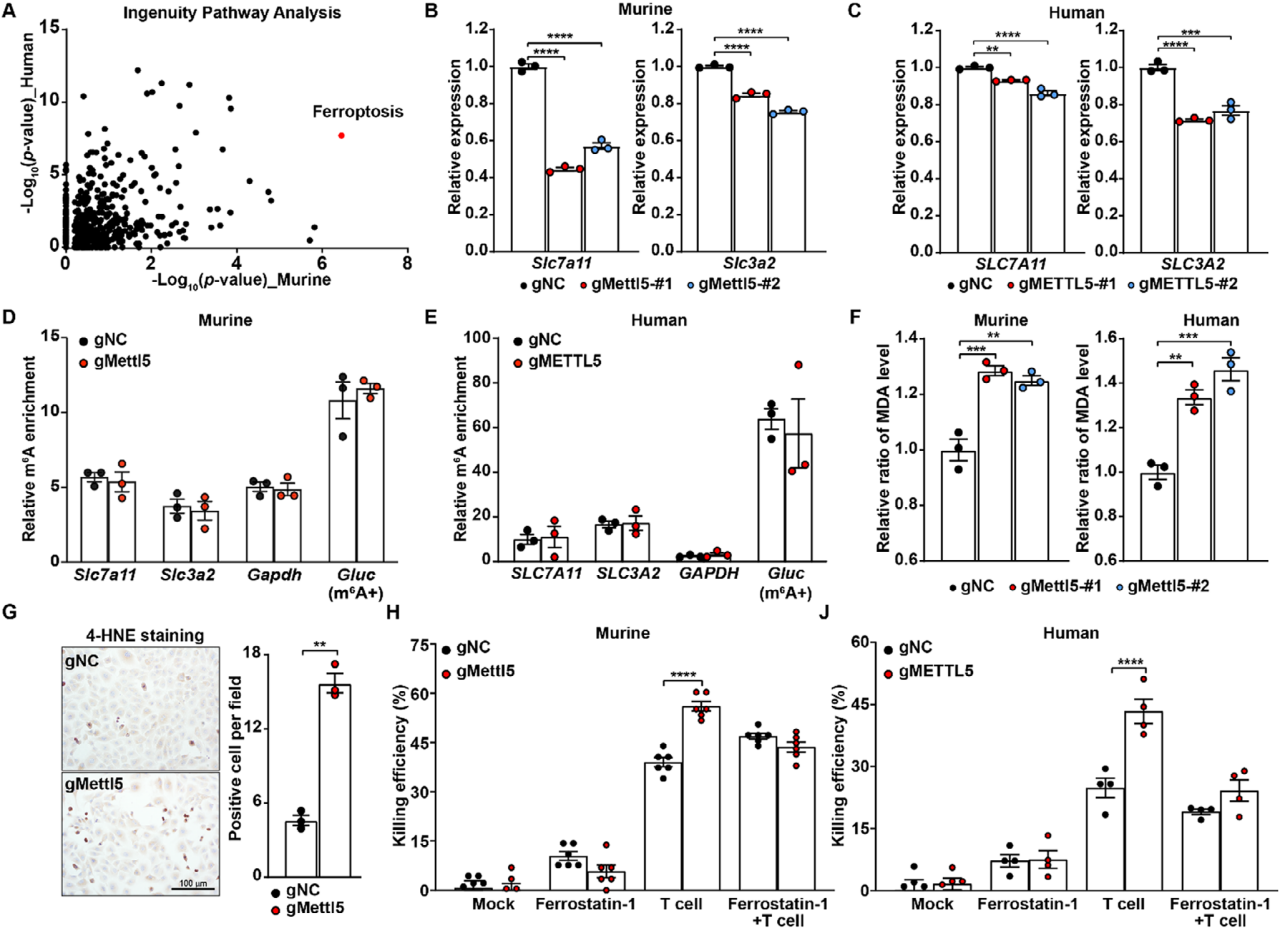
Genetic depletion of *METTL5* enhances T cell‐induced tumor ferroptosis by downregulating *SLC7A11* and *SLC3A2* expression. A) IPA of DEGs in human and murine ovarian cancer cell lines with *METTL5* KO compared to the negative controls. DEGs were defined as genes with |Log_2_FC| > 0.25 and *p* < 0.05. Results of the canonical pathways with statistically significant enrichment were plotted. The “Ferroptosis signaling pathway” is highlighted in red. B,C) Transcription level changes in *SLC7A11* and *SLC3A2* following *METTL5* knockout. Real‐time PCR was used to determine transcription levels of *SLC7A11* and *SLC3A2* in murine B) and human C) ovarian cancer cells. Data were analyzed using one‐way ANOVA followed by Dunnett's post hoc test and were presented as mean values ± SEM (*n* = 3). D,E) m^6^A levels of *SLC7A11* and *SLC3A2* mRNA in tumor cell lines with *METTL5* genetic depletion. Anti‐m^6^A IP‐qPCR was performed to determine the m^6^A levels of selected genes (*SLC7A11, SLC3A2, GAPDH*) in murine D) and human E) *METTL5*‐KO and control cell lines. A Gaussia luciferase (Gluc) RNA served as the m^6^A modification control. Data were analyzed using one‐way ANOVA followed by Dunnett's post hoc test and were presented as mean values ± SEM (*n* = 3). F) Lipid peroxidation level changes after *METTL5* KO. Malondialdehyde (MDA) levels were measured to assess lipid peroxidation in murine (left) and human (right) ovarian cancer cells with *METTL5* KO compared to the negative controls. Data were analyzed using one‐way ANOVA followed by Dunnett's post hoc test and were presented as mean values ± SEM (*n* = 3). G) Immunochemistry staining of 4‐HNE. ID8/GC cells with or without *Mettl5* KO were processed for 4‐HNE staining. Positive cells were quantified from at least three independent microscopic fields per sample. Data were analyzed using Student's *t*‐test and were presented as mean ± SEM (*n* = 3). H–J) Tumor sensitivity to T cell‐mediated killing with or without ferroptosis inhibition. Murine H) and human J) ovarian cancer cells with *METTL5* knockout or the negative controls were pretreated with DMSO (mock) or 2 × 10^−6^ m Ferrostatin‐1 for 24 h. An equal number of pretreated tumor cells were seeded and then co‐cultured with tumor‐reactive T cells. After overnight coculture, the number of live tumor cells was counted, and the killing efficiency was calculated. Data were analyzed using one‐way ANOVA followed by Dunnett's post hoc test and were presented as mean values ± SEM (*n* = 4–6). Representative data from at least two independent experiments were shown. **p < 0.01; ***, *p* < 0.001; **** *p* < 0.0001.

To further explore immunoregulatory pathways controlled by METTL5 in OC, we analyzed mRNA expression levels in murine and human OC cells with and without *METTL5* KO. We confirmed that *METTL5‐*KO cells are well separated from corresponding controls based on their transcriptomic profiles (Figure S7A, Supporting Information). Although there are limited numbers of DEGs overlapping between murine and human OC cells (Figure S7B, Supporting Information), Ingenuity Pathways Analysis using DEGs in *METTL5*‐KO cells revealed that *METTL5* KO consistently alters expression levels of genes encoding ferroptosis components (**Figure** **5**A and Table S7, Supporting Information). Among the identified DEGs in the ferroptosis pathway (Figure S7C, Supporting Information), we validated reduced *SLC7A11* and *SLC3A2* expression by *METTL5* KO in both murine and human OC cells (Figure 5B,C). However, we didn't observe any changes in the m^6^A methylation levels of *SLC7A11* and *SLC3A2* mRNA transcripts in *METTL5*‐KO cells (Figure 5D,E). Furthermore, we measured levels of malondialdehyde (MDA), a biomarker of ferroptosis,^[^ ^49^ ^]^ and found that genetic depletion of *METTL5* significantly increased MDA levels in OC cells (Figure 5F). Additionally, we performed immunostaining for 4‐hydroxynonenal (4‐HNE), another established marker of lipid peroxidation, on genetically modified OC cell lines and related tumor tissues. Our results demonstrate that *Mettl5* knockdown significantly increased 4‐HNE levels in both in vitro and in vivo samples (Figure 5G and Figure S7D, Supporting Information). By pretreating OC cells with Ferrostatin‐1, a ferroptosis inhibitor, *METTL5*‐KO OC cells showed similar sensitivity to killing induced by paired tumor‐reactive T cells as control tumor cells (Figure 5H,J). On the other hand, *METTL5*‐KO cells that received mock pretreatment remained more sensitive to T cell killing than controls (Figure 5H,J). These results support that inhibition of the ferroptosis pathway can reverse the immune phenotype of *METTL5*‐KO cells.

### 2.5 METTL5 Suppresses Ferroptosis Activity by Increasing ATF4 Translation

**Figure 6:**
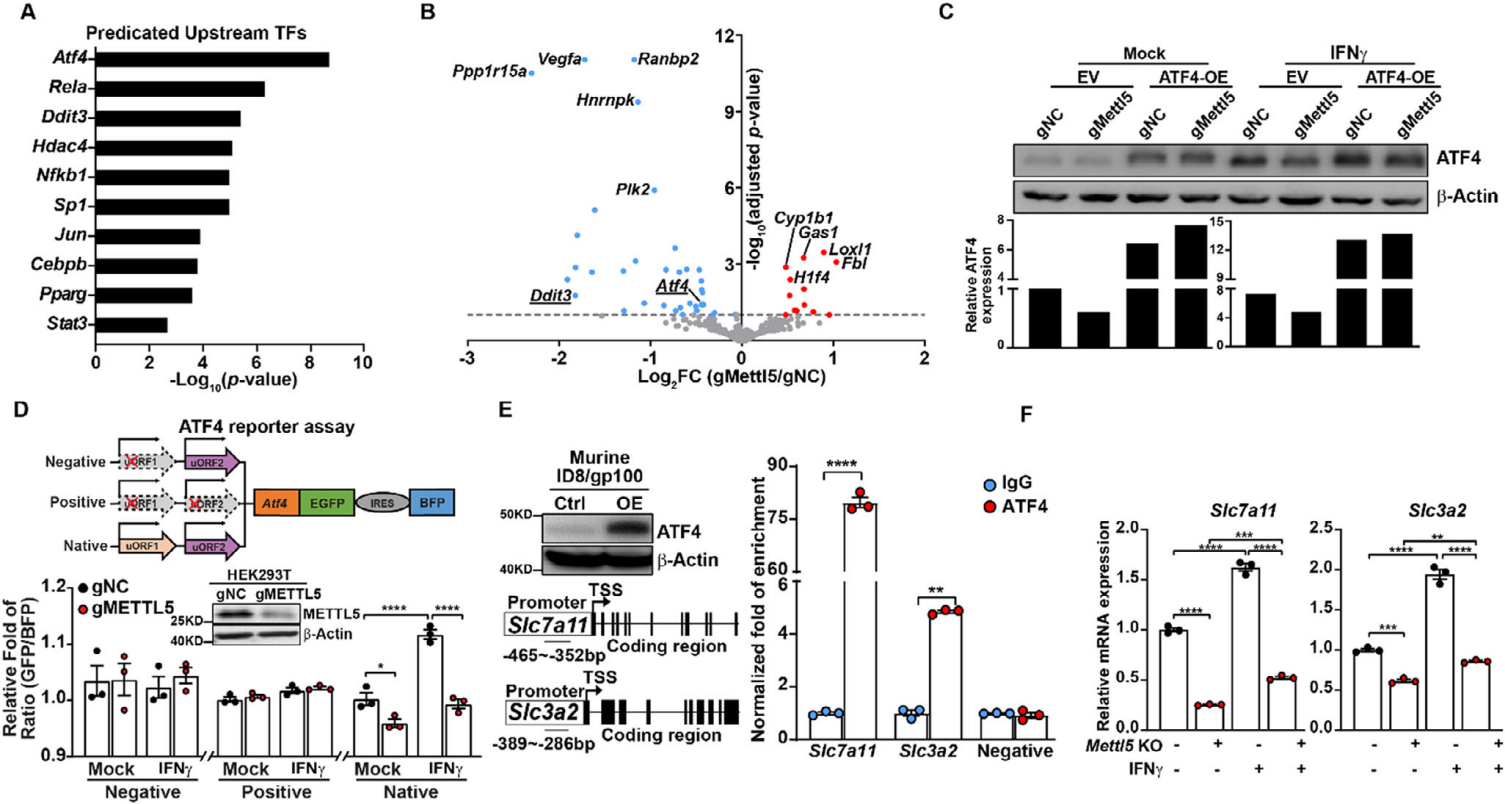
METTL5 positively regulates ATF4 translation in OC cells. A) Prediction of upstream transcription factors. Enrichment analysis of upstream TFs was conducted for transcripts downregulated in two *METTL5‐*KO and negative control ID8/GC cell lines. The top 10 significantly enriched upstream TFs were identified based on *p*‐values. B) Active ribosome profiling of *METTL5‐*KO and the negative control ID8/GC cells. RiboLace assays were performed to identify genes with altered active ribosome binding. Genes with significantly reduced ribosome binding are labeled in blue, and those with increased ribosome binding are labeled in red. C) The levels of ATF4 protein expression with and without IFNγ treatment. Genetically modified ID8/GC cells were treated with 5 ng mL^−1^ of IFNγ for 24 h. ATF4 and β‐actin protein levels were measured by western blot, and the ratio of ATF4 to β‐actin intensity was calculated. Data were presented as mean values. D) Changes in ATF4 translational efficiency in response to *METTL5* knockout. HEK293T cells with *METTL5* KO and control cells were transfected with reporter plasmids containing either native or mutated upstream open reading frames (uORFs) of *Atf4*. Cells were treated with 5 ng mL^−1^ of IFNγ for 24 h, and means of fluorescence intensity (MFIs) for GFP and BFP were measured. The ratio of MFIs between GFP and BFP was calculated to determine ATF4 translational efficiency. Data were analyzed using one‐way ANOVA followed by Dunnett's post hoc test and were presented as mean values ± SEM (*n* = 3). E) Chromatin immunoprecipitation (ChIP) assay for ATF4 binding to *Slc7a11* and *Slc3a2* promoter regions in murine ovarian cancer cells. Western blot analysis confirmed ATF4 protein expression (upper panel). A schematic diagram illustrates primer design for ATF4 binding sites (lower panel). ChIP‐qPCR was used to detect ATF4 occupancy at major peak regions within the *Slc7a11* and *Slc3a2* promoter regions in ID8/gp100 cells ectopically expressing ATF4 (OE). Relative abundance was normalized to inputs. Data were analyzed using one‐way ANOVA followed by Dunnett's post hoc test and were presented as mean values ± SEM (*n* = 3). F) Transcription level changes of *Slc7a11* and *Slc3a2* in *METTL5* KO and negative control cell lines. Cells were treated with 5 ng mL^−1^ of IFNγ for 24 h and followed by real‐time PCR analysis to determine *Slc7a11* (left) and *Slc3a2* (right) transcription levels. Data were analyzed using one‐way ANOVA followed by Dunnett's post hoc test and were presented as mean values ± SEM (n = 3). Representative data from at least two independent experiments were shown. **p < 0.01; ***, *p* < 0.001; **** *p* < 0.0001.

Our results suggest that METTL5 does not directly modify m^6^A methylation of *SLC7A11* and *SLC3A2* transcripts, but instead controls 18S rRNA methylation. Thus, we hypothesized that the immunological roles of METTL5 could be mediated by its impact on the translation of transcription factors (TFs) regulating the transcription of *SLC7A11* and *SLC3A2*. To investigate this, transcription factor enrichment analysis was performed on DEGs identified in *Mettl5*‐KO murine OC cells, revealing a set of potential TFs that regulate the expression of genes impacted by *Mettl5* knockout. Among these, *Atf4*, *Ddit3*, and *Cebpb*, critical regulators of Integrated Stress Response (ISR), emerged as potential targets affected by *Mettl5* knockout (**Figure** **6**A). Next, translational efficiency (TE) of genes in murine OC cells with or without *Mettl5* KO was assessed by a RiboLace assay, which provides active ribosome profiles.^[^ ^50^ ^]^ By integrating the RiboLace data with transcriptomic profiles, we identified 1241 transcripts with upregulated TE and 1112 with downregulated TE, indicating significant translational reprogramming driven by *Mettl5* genetic depletion (Figure S8A, Supporting Information). The most enriched pathway among downregulated transcripts was the pathway of “Response of EIF2AK1 (HRI) to heme deficiency” which includes *Atf4*, *Ddit3*, and *Cebpb* (Figure S8B,C, Supporting Information). These results confirmed that *Mettl5* KO significantly reduced the translations of TFs involved in ISR in OC cells (Figure 6B and Figure S8C, Supporting Information). To examine the role of METTL5‐dependent m^6^A modification at position 1832 in 18S rRNA, structural analysis was conducted using Cryo‐EM (PDB: 4ug0). The local environment of m^6^A1832 revealed π‐π stacking between C1833, m^6^A1832, and A1831, along with a unique base pairing between m^6^A1832 and Cm 1703, featuring a single hydrogen bond (Figure S9A, Supporting Information). This suggests that the lack of methylation at A1832 could disrupt these interactions, thereby impacting the local structure. The m^6^A1832 site is situated within helix 44 (h44) of the 18S rRNA, a critical region for mRNA binding and decoding (Figure S9B, Supporting Information). Therefore, disturbances in this area could impair the assembly of the 40S ribosomal subunit, leading to aberrant ribosome scanning (Figure S9C, Supporting Information).

To further characterize the features shared among genes under the regulation of METTL5, we compared the changes in translation efficiency in response to *Mettl5* KO among transcripts with varying Kozak sequence strengths, which directly influence the ribosome scanning process, as previously classified.^[^ ^51^ ^]^ An increased proportion of transcripts with strong Kozak sequences showed reduced translation in gMettl5 cells (Figure S10A, Supporting Information). Notably, among these transcripts with reduced translation, transcripts regulating “Cellular responses to stress (GO: 0033554)” had the highest prevalence of strong Kozak sequences, suggesting a potential mechanism underlying their translational repression (Figure S10A, Supporting Information). Particularly, both *Atf4* and *Ddit3* contained multiple strong Kozak sequences, and *Atf4*’s two upstream open reading frames (uORFs) made it particularly sensitive to ribosome scanning defects caused by *Mettl5* depletion (Figure S10B,C, Supporting Information). These findings suggest that METTL5‐mediated m^6^A modifications at the 1832 site are critical for proper ribosomal assembly, ribosome scanning, and *Atf4* translation.

Given that ATF4 has been implicated in regulating *SLC7A11* and *SLC3A2* expression in both tumor and immune cells,^[^ ^52^, ^53^ ^]^ we focused on ATF4 for further mechanistic studies. Both western blot analyses and ATF4 native reporter assays consistently showed that *METTL5* depletion reduces *ATF4* translation both in the absence and presence of IFNγ (Figure 6C,D). However, when we carried out the reporter assay using two controls—one negative control reporter with a mutated uORF1 that continuously suppressed EGFP translation, and one positive control reporter with mutations in both uORF1 and uORF2 that allowed EGFP translation to remain consistently active—we found that *METTL5* knockout did not influence EGFP translation in either case regardless of IFNγ treatment (Figure 6D). These results further validate that the METTL5 regulates ATF4 translation by altering ribosome scanning. Since IFNγ is one of the major immune stimulants that induce tumor cell stress, our results suggest that METTL5 continues to regulate *ATF4* translation in OC cells even under the immune attack. Furthermore, we evaluated the interaction between ATF4 and the promoter regions of *Slc7a11* and *Slc3a2* in murine OC cells by chromatin immunoprecipitation analysis. Compared with the promoter region of an irrelevant gene region, significantly enriched binding of ATF4 in the promoter regions of *Slc7a11* and *Slc3a2* can be observed (Figure 6E). In the presence of IFNγ, depletion of *Mettl5* continues to downregulate expression of *Slc7a11* and *Slc3a2* in ID8 cells (Figure 6F).

### 2.6 Overexpression of *ATF4* Reverses the Immune‐Sensitive Phenotype of *METTL5*‐KO cells

**Figure 7:**
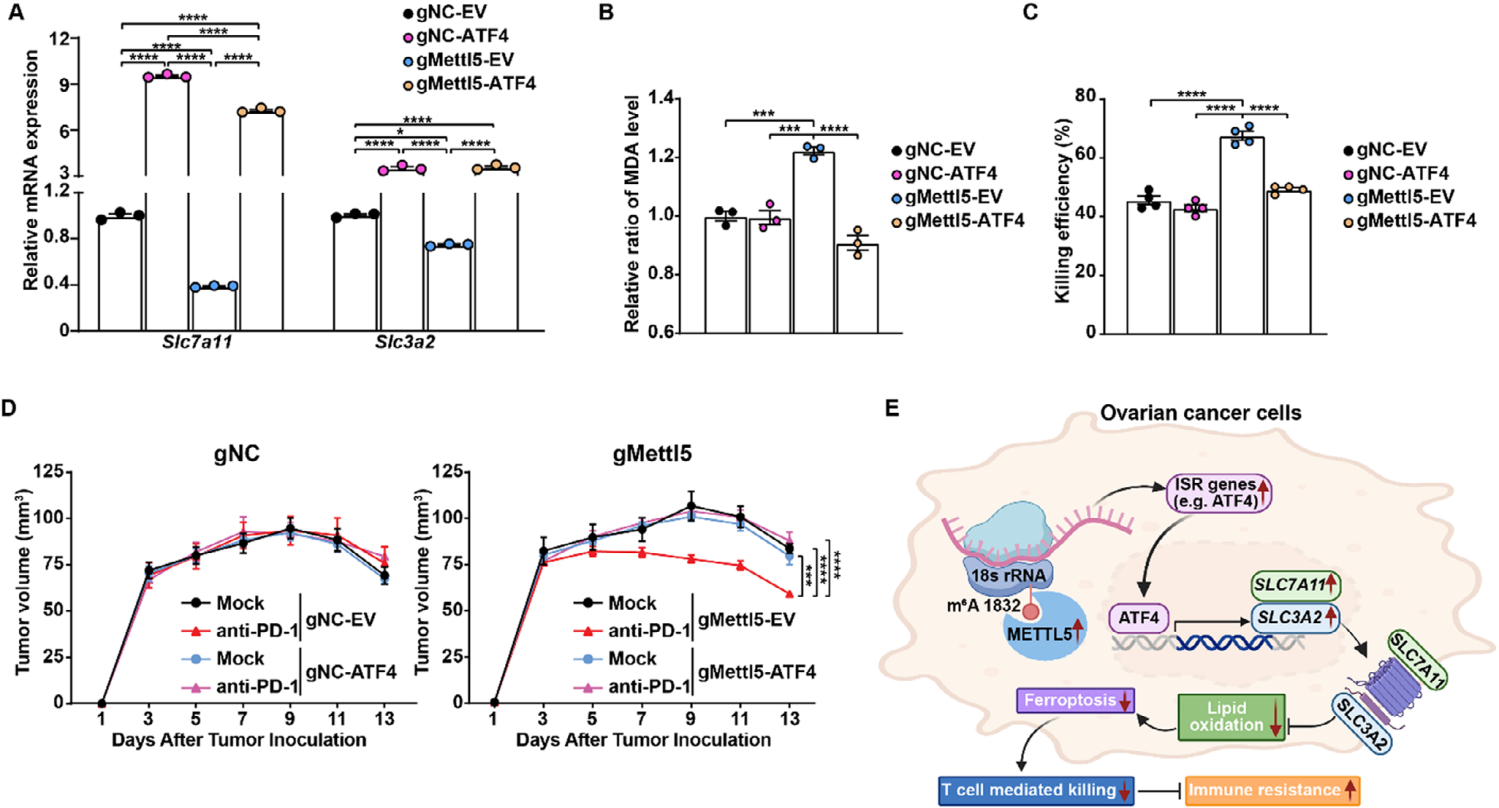
Overexpression of *Atf4* reduces ferroptosis and abolishes enhanced anti‐PD‐1 sensitivity in *METTL5‐*KO OC cells. A) Transcriptional changes of *Slc7a11* and *Slc3a2* after reintroducing *Atf4* expression in murine OC cells. Real‐time PCR was used to quantify *Slc7a11* and *Slc3a2* transcription levels in ID8/GC‐gNC and gMettl5 cells without and with *Atf4* overexpression, respectively. Data were analyzed using one‐way ANOVA followed by Dunnett's post hoc test and were presented as mean values ± SEM (*n* = 3). B) Lipid peroxidation level changes after reintroducing *Atf4* expression in murine OC cells. Lipid peroxidation levels were assessed by measuring MDA levels in gNC and gMettl5 cells without and with *Atf4* overexpression, respectively. Data were analyzed using one‐way ANOVA followed by Dunnett's post hoc test and were presented as mean values ± SEM (*n* = 3). C) Tumor sensitivity to T cell‐mediated killing after reintroducing *Atf4* expression in murine OC cells. An equal number of genetically modified murine OC cells were co‐cultured with tumor‐reactive Pmel T cells. After overnight co‐culture, the number of live tumor cells was counted, and killing efficiency was calculated. Data were analyzed using one‐way ANOVA followed by Dunnett's post hoc test and were presented as mean values ± SEM (*n* = 4). (D) *Atf4* overexpression abolishes enhanced anti‐PD‐1 sensitivity induced by *Mettl5* depletion. ID8/GC‐gNC cells (left panel) and –gMettl5 cells (right panel) were inoculated into C57BL/6 mice. Three days after tumor inoculation, mice with measurable tumors were randomized and treated intraperitoneally with either PBS or anti‐PD‐1 (100 µg per dose). Data were analyzed using two‐way ANOVA followed by multiple comparison test and were presented as mean values ± SEM (*n* = 5). E) Schematic representation of the immunoregulatory role of METTL5 in ovarian cancer cells. The schema was created with BioRender.com. Representative data from at least two independent experiments were shown. ***p < 0.001; ****p < 0.0001.

To functionally explore the contribution of ATF4 to the immune phenotype observed in *METTL5*‐KO cells, we overexpressed *Atf4* in gNC and gMettl5 ID8/GC cells to generate gNC‐ATF4 and gMettl5‐ATF4 cell lines, respectively. gNC and gMettl5 ID8/GC cells transduced with the empty vector (EV) were used as corresponding controls (gNC‐EV and gMettl5‐EV). Overexpressing *Atf4* increased expression of *Slc7a11* and *Slc3a2* in murine OC cells with and without *METTL5* KO (**Figure** **7**A). In addition, overexpressing *Atf4* also suppresses lipid peroxidation in *METTL5*‐KO ID8/GC cells (Figure 7B). Although overexpressing *Atf4* has a limited impact on tumor sensitivity to in vitro T cell killing, gMettl5 ID8/GC cells with overexpressed *Atf4* display reduced T cell‐induced cell death when compared to gMettl5‐EV cells (Figure 7C). Consistently, overexpressing *Atf4* in gNC tumors has a limited impact on the in vivo antitumor effect of anti‐PD‐1 (Figure 7D). However, overexpressing *Atf4* completely abolished anti‐PD‐1 efficacy in mice bearing gMettl5 ID8/GC tumors (Figure 7D).

Collectively, our unbiased approaches using genetic screens and clinical data mining reveal that METTL5 functions as an OC‐intrinsic immune regulator. As shown in Figure 7E, *METTL5* expression is commonly upregulated in OC cells. By modulating the 18S rRNA m^6^A levels, increased METTL5 activity affects ribosome scanning and enhances translations of transcripts with strong Kozak sequences, particularly those related to cell stress responses. Among these transcripts, upregulated ATF4 expression enhances the transcription of *SLC7A11* and *SLC3A2*, which import cystine for antioxidant defense. Ultimately, hyperactivation of METTL5 results in immune resistance of OC cells by suppressing ferroptosis activity. Overall, our data indicate that the axis of METTL5‐ATF4‐SLC7A11/SLC3A2 plays a vital role in promoting immune resistance in ovarian cancer models and patients, and targeting METTL5 could improve the effectiveness of immunotherapy in OC patients.

## 3 Discussion

We report our effort to integrate the powers of genetic screens and correlation analysis of clinical datasets to functionally interrogate the immunological roles of molecules expressed in ovarian tumors. This effort leads to the discovery of a set of OC‐intrinsic factors with the potential to control T cell‐mediated antitumor immune responses. Among them, we unveil an unreported immunological role of METTL5. Although *METTL5* expression does not correlate with overall survival and disease stages in cancer patients, HGSOC patients with upregulated *METTL5* display unfavorable clinical outcomes in response to ICB treatment. Mechanistic studies showed that the axis of METTL5‐ATF4‐SLC7A11/SLC3A2 modulates tumor sensitivity to T cell‐mediated antitumor immune activity in ovarian cancers. These findings not only outline the landscape of immunosuppressive mechanisms in OC, but also pinpoint the potential of targeting METTL5 to overcome immune resistance in OC patients.

The METTL family includes more than 30 members containing methyltransferase activity to a wide range of substrates, including ribosomal RNA (rRNA), mRNA, and lncRNA.^[^ ^54^ ^]^ As RNA methylation regulates RNA maturation, nuclear export, transcript splicing, translation, stability, and degradation, the METTL family members are widely involved in cellular events critical for cancer development and metastasis.^[^ ^55^ ^]^ TCGA Pan‐Cancer analyses showed that upregulation of several METTLs, including METTL1, 2A, 2B, 5, and 6, inversely correlates with overall survival of cancer patients.^[^ ^56^ ^]^ In addition, METTL3 and its binding partner METTL14 are two members with established roles in cancer biology. The complex of METTL3/METTL14 is responsible for around 95% of m^6^A methylation of mRNAs.^[^ ^54^ ^]^ METTL3/METTL14 can act as oncogenic proteins by maintaining the stability of chromosomes and telomeres in cancer cells and promoting uncontrolled proliferation.^[^ ^54^ ^]^ METTL3/METTL14 also enhances the activation of p53 signaling in response to DNA damage by stabilizing p53 protein.^[^ ^57^ ^]^ Via this mechanism, METTL3/METTL14 exhibits tumor suppressor activity in some contexts. However, the roles of other METTL family proteins in cancers remain poorly characterized.

METTL5 is the m^6^A methyltransferase that modifies adenosine 1832 of 18S rRNAs, and this function requires its binding to TRMT112.^[^ ^24^, ^58^ ^]^ *METTL5*‐KO mice are viable but display reduced body size and body fat as shown in two independent studies.^[^ ^23^, ^58^ ^]^ *Mettl5* KO downregulates the expression of genes involved in lipid biosynthesis and storage.^[^ ^58^ ^]^ Although there is no obvious cardiac abnormality in *METTL5‐*KO mice, cardiac‐specific *Mettl5* KO was reported to promote stress‐induced cardiac remodeling.^[^ ^59^ ^]^ Regarding cancer biology, the involvement of METTL5 is mainly studied in HCC. It was reported as a diagnostic and prognostic biomarker in HCC patients.^[^ ^54^, ^60^ ^]^ Upregulation of METTL5 in HCC cells promotes c‐Myc stability and activates the glycolysis pathway, resulting in HCC progression.^[^ ^61^ ^]^ METTL5 depletion was found to decrease the in vitro proliferation of HepG2 cells derived from a human HCC patient.^[^ ^58^ ^]^ Aside from HCC, METTL5 depletion was found to suppress translation of transcripts enriched in the TGFβ pathway which contributes to the progression of intrahepatic cholangiocarcinoma.^[^ ^62^ ^]^ Upregulation of *METTL5* is also correlated with advanced disease in patients with nasopharyngeal carcinoma and kidney cancer, and *METTL5* KO in these types of cancer lines exerts oncogenic functions by regulating cell cycle and apoptosis.^[^ ^63^, ^64^ ^]^ Supporting these observations, our analysis of TCGA data shows that most cancer types display elevated *METTL5* expression. Although upregulated *METTL5* is observed in OC patients, its expression has no relationship with cancer stage or overall survival. When we knocked out *METTL5* in both human and murine OC lines (SKOV3 and ID8), the in vitro and in vivo growth rates of these cells were largely comparable with control cells. *METTL5* depletion also has no significant impact on the in vitro growth of HCT116, a p53‐positive colon carcinoma line.^[^ ^65^ ^]^ Together, these findings suggest that the correlation of *METTL5* expression and cancer progression could be variable and dependent on cancer types, which is similar to other METTLs with multifaceted functions.^[^ ^54^ ^]^

Interestingly, our unbiased genetic screens divulge the link between METTL5 and immune resistance in OC. *METTL5* expression is inversely correlated with an antitumor immune signature observed in OC and TNBC patients receiving ICB treatment. Mechanistic studies demonstrate that the immunological role of METTL5 is dependent on the regulation of ATF4. *METTL5* depletion was found to decrease the translational efficiency of ATF4 by altering the levels of m^6^A‐modified 18S rRNA in both human and murine OC cells. Although the absence of *Mettl5* in mouse embryonic stem cells and several human nasopharyngeal carcinoma cell lines was reported to result in a decrease in the global translation rate,^[^ ^23^, ^63^ ^]^ our ribosome profiling results reveal that translational changes associated with *METTL5* knockout are more pronounced in transcripts containing strong Kozak sequences when compared to those lacking them. Similar to the report demonstrating that *METTL5* regulates stress responses in *C. elegans*,^[^ ^66^ ^]^ our data indicate that *METTL5* knockout significantly reduces the translation efficiency of cell stress‐related genes with strong Kozak sequences. Structural analysis further indicates that modifications to m^6^A can lead to irregular assembly of the 40S ribosomal subunit, particularly affecting the h44 helix region. These disruptions are predicted to result in aberrant ribosome scanning, with mRNAs harboring strong Kozak sequences and upstream open reading frames (uORFs) being the most affected. ATF4 exemplifies this sensitivity, as its transcript contains a strong Kozak sequence and multiple uORFs, rendering it highly susceptible to disruptions in ribosome scanning. Thus, *METTL5* depletion can trigger abnormal ribosome scanning by skipping the uORF1 of *ATF4*, a positive‐acting translation element. Therefore, reduced m^6^A modification of 18S rRNA exhibits a significant suppressive effect on ATF4 levels in OC cells with *METTL5* depletion. Similar regulatory mechanisms of ATF4 by METTL5 were also reported in melanoma cell lines.^[^ ^67^ ^]^

Furthermore, *METTL5* was shown to positively control PD‐L1 expression, the ligand of a T cell inhibitory receptor, in human HCC lines.^[^ ^68^ ^]^ Besides PD‐L1, our clinical data demonstrated that expression levels of multiple IFN‐related genes are lower in *METTL5*‐High tumors from OC patients. However, we did not observe any significantly reduced expression in PD‐L1 and IFN‐related genes in OC lines upon *METTL5* depletion, suggesting that these effects might not be directly controlled by METTL5. Whereas our multiomic analysis suggests that the axis of METTL5‐ATF4 dramatically influences the ferroptosis pathway in OC cells. Unlike apoptosis and necrosis, ferroptosis, triggered by lipid peroxidation,^[^ ^69^ ^]^ is an iron‐dependent form of cell death. Activation of ferroptosis has been implicated in overcoming resistance to chemotherapy, radiotherapy, and immunotherapy in cancer patients.^[^ ^70^ ^]^ HGSOC tumors also show increased iron retention and are susceptible to Erastin, a ferroptosis inducer.^[^ ^71^ ^]^ Recent studies indicate that tumor‐reactive CD8^+^ T cells can induce not only apoptosis but also ferroptosis. IFNγ produced by CD8^+^ T cells increases the activity of lipid peroxidation in tumor cells.^[^ ^25^ ^]^ In addition, IFNγ upregulates *ACSL4* expression via STAT1 and IRF1 signaling and reprograms tumor fatty acid metabolism.^[^ ^26^ ^]^ Through these two nonoverlapping mechanisms, tumor‐reactive CD8^+^ T cells control tumor growth by inducing ferroptosis. Our data demonstrate that inhibition of METTL5 sensitizes OC to T cell‐mediated ferroptosis by downregulating *SLC7A11* and *SLC3A2*. Ferroptosis inhibition and overexpression of ATF4 completely reverse the phenotype of *METTL5* KO cells in response to T cell killing.

Finally, we observed significant positive correlations between the expressions of *METTL5* and *DDIT3*, a surrogate marker for ATF4 activity, in both TCGA‐OC and MDACC‐HGSOC datasets (Figure S11A, Supporting Information). However, our in vitro genome‐wide immune screen results showed that knockout of *Atf4*, *Ddit3*, *Slc7a11*, or *Slc3a2* alone did not significantly enhance tumor cell sensitivity to T cell–mediated killing (Figure S11B, Supporting Information). Unlike *METTL5*, DEGs associated with upregulated expression of *DDIT3*, a gene regulated by ATF4, fail to consistently show significant enrichment within either the ICB‐responder or nonresponder gene sets (Fig S11C). These findings suggest that disruption of single downstream effectors, either ATF4 or individual ferroptosis‐related molecules, might not be sufficient to overcome immune resistance, which is observed in OC cells expressing high *METTL5*.

Due to the restricted availability of murine syngeneic ovarian cancer models, our in vivo experiments primarily utilized the ID8 line. We are actively working to establish additional syngeneic and humanized ovarian cancer models with better representation of the genetic landscape of OCs to further determine whether oncogenic alterations, such as *TP53* mutation, influence the METTL5/ATF4/Ferroptosis axis in tumor immune evasion. Moreover, future studies are needed to reveal whether additional ISR genes can mediate the immunological role of METTL5.

Taken together, we identified METTL5 as one of the most important OC‐resistant factors through unbiased in vitro and in vivo screens. This discovery was further validated using a unique OC‐ICB patient cohort. The m^6^A‐modified mRNA levels in *METTL5*‐KO OC lines are comparable with those in control lines. Instead, our multiomic analyses indicate that METTL5 modulates immune resistance via ATF4, revealing a previously unrecognized pathway linking rRNA methylation to tumor immune evasion. By elucidating the immunological role of METTL5 through its rRNA methylation function in OC, our findings provide mechanistic insights that could inform the development of new immuno‐oncology combinations. These insights warrant further studies to evaluate the potential of METTL5 as a prognostic biomarker and therapeutic target for ICB‐based therapies in ovarian cancer patients.

## 4 Experimental Section

### Cell Lines and Mice

The parental and genetically modified ID8 (#SCC145, Millipore‐Sigma, Burlington, MA) lines were cultured in Dulbecco's modified Eagle's medium (DMEM; #11965118, ThermoFisher Scientific, Waltham, MA) supplemented with 5% heat‐inactivated fetal bovine serum (FBS; #S11150, R&D System, Minneapolis, MN), 5 µg mL^−1^ of insulin, transferrin and sodium selenite (1×ITS, #I3146, Millipore‐Sigma) and 100 µg mL^−1^ of Normocin (#ant‐nr‐1, InvivoGen, San Diego, CA). Human embryonic kidney (HEK) 293T and human ovarian cancer cell line SKOV3 were obtained from the American Type Culture Collection (#CRL‐3216 and #HTB‐77, ATCC, Bethesda, MD) and cultured in DMEM supplemented with 10% heat‐inactivated FBS and 100 µg mL^−1^ of Normocin. All cell lines with stable expression of gRNAs generated in this study were cultured under the treatment of 2 µg/ml of puromycin (#A1113803, Gibco, Carlsbad, CA) with the culture media described above. Cells grew at 37 °C with 5% CO_2_. All cell lines were authenticated by short tandem repeat fingerprinting or the expression of tagged markers used for genetic modification. The mycoplasma detection kit (#13100‐01, SouthernBiotech, Birmingham, AL) was used to routinely monitor for mycoplasma contamination of cultured cells. The maximum length of time of in vitro cell culture between thawing and use in the described experiments is two weeks.

Pmel‐1 TCR/Thy1.1 (Pmel) transgenic mice, C57BL/6 mice (556NCIC57BL, Charles River Laboratories, Wilmington, MA) and NOD.Cg‐*Prkdc* ^scid^ *Il2rg* ^1Wjl^/SzJ (NSG) (#005557, Jackson Laboratories, Bar Harbor, ME) mice were maintained in a specific pathogen‐free barrier facility at the University of Houston. All animal experiments were approved by the University of Houston Institutional Animal Care and Use Committee (IACUC; PROTO201800058, “The combination therapy in cancer treatment”).

### Generation of Tumor‐Reactive T Cells

Murine gp100‐specific T cells from Pmel mice and human B7H3 (hB7H3)‐chimeric antigen receptor (CAR) T cells were used to target gp100‐expressing ID8 and SKOV3 cells in in vitro killing assays, respectively. For gp100‐specific T cells, splenocytes were isolated from Pmel mice and cultured in complete RPMI1640 media (10% FBS, 20 mm HEPES, 1 mm sodium pyruvate, 50 × 10^−6^ m 2‐mercaptoethanol, 2 × 10^−3^ m l‐glutamine and 100 µg mL^−1^ of Normocin) supplied with 500 IU mL^−1^ recombinant human interleukin‐2 (rhIL‐2; Prometheus Laboratories, NDC Code 65483‐116‐07, San Diego, CA) and 0.3 µg mL^−1^ anti‐mouse CD3 (#555273, BD Biosciences, San Jose, CA) for 24 h. After stimulation, cells were maintained in the culture medium with 500 IU mL^−1^ IL‐2 for at least 6 days. After 6 to 10‐day culture, Pmel T cells were used in the described experiments. hB7H3‐CAR T cells was a generous gift from Dr. Yong Lu (Houston Methodist Research Institute). To generate hB7H3‐CAR T cells, CD3^+^ T cells were isolated from peripheral blood mononuclear cells (PBMCs) from healthy donors, and stimulated with anti‐CD3/CD28 Dynabeads (311161D, ThermoFisher Scientific) in the presence of 200 IU mL^−1^ rhIL‐2. After 24‐h stimulation, T cells were transduced with retrovirus expressing hB7H3‐(SS1)‐hBBZ‐CAR in the presence of 10 µg mL^−1^ protamine sulfate (#PHR2610, Millipore‐Sigma) as previously described,^[^ ^72^ ^]^ then expanded in the presence of 200 U mL^−1^ rhIL‐2 for an additional 7–8 days before scheduled experiments. Transduction rates in generating hB7H3‐CAR T cells were > 85%.

### Genetical Modification of Tumor Cell Lines

Lentivirus‐based gene delivery was used to either genetically knockout (KO) gene‐of‐interests (GOIs) or ectopically express GOIs. To generate lentiviral supernatants, HEK293T cells were seeded 16 h before transfection and transfected with the lentiviral vectors encoding different gRNAs or GOIs, along with lentiviral packaging plasmids, pCMV‐VSV‐G and psPAX2 (#8454 and #12260, Addgene, Watertown, MA) by the jetPRIME transfection reagent (#101000046, VWR, Radnor, PA) according to the manufacturer's protocol. Viral supernatants were collected 72 h post‐transfection and filtered by 0.45 µm Polyvinylidene fluoride (PVDF) Syringe Filter Unit (#SLHV033NK, Millipore‐Sigma) to remove cell debris. Lentivirus at designated titers was used to infect cells in the presence of 8µg/ml hexadimethrine bromide (#107689, Sigma–Aldrich, St Louis, MO).

To generate ID8 cells expressing gp100 (tagged by IL‐4R), the ID8 cell line was transduced with lentivirus expressing gp100 as previously described.^[^ ^73^ ^]^ The gp100‐expressing ID8 cell line was generated by staining and sorting IL‐4R^+^ (#552178, BD Biosciences) cells at 72 h after lentivirus transduction. To generate ID8 and SKOV3 cells expressing Cas9 tagged by GFP for gene editing, ID8 and SKOV3 cell lines were transduced lentivirus expressing lentiCas9‐EGFP (#63952, Addgene). The Cas9 expressing ID8‐gp100 (ID8/GC) line and the Cas9 expressing SKOV3 (SKOV3/C) line were generated by sorting GFP^+^ cells at 72 h post‐lentivirus transduction. To genetically suppress the expression of GOIs in cells, lentiviral gRNA‐expressing vectors were constructed. Fully synthesized double‐strand (ds) DNA fragments (Twist Bioscience, San Francisco, CA) encoding gene‐specific gRNAs were inserted into the pKLV2‐U6gRNA5(BbsI)‐PGKpuro2ABFP‐W (pKLV2; #67974, Addgene) as previously described.^[^ ^5^ ^]^ The forward sequences of protospacer sequences of gRNAs were listed in Table S8 (Supporting Information). ID8/GC and SKOV3/C cells were transduced with gRNA‐expressing vectors and selected by 2 µg/ml of puromycin to generate stable KO cell lines. Cells transduced with the viral vector encoding a nontargetable gRNA (NC) were generated and served as control cells. To ectopically express GOIs in cells, dsDNA fragments encoding FLAG‐tagged mouse open reading frames (ORFs) of *Atf4* (NM_0 09716.3) were inserted into the lentiviral vector, pLVX‐EF1α‐IRES‐mCherry (#631987, Takara Bio, San Jose, CA). ID8/GC cells were transduced with lentiviral ORF vectors. Stable cell lines were generated by sorting mCherry^+^ cells at 72 h post‐lentivirus transduction. Cells transduced with the corresponding empty vector were generated and served as control cells.

### In Vitro Genome‐Wide Immune Screen in ID8/GC Cells

The mouse genome‐wide knockout CRISPR Library version 2, consisting of 90230 gRNAs targeted 18424 genes was purchased from Addgene.^[^ ^74^ ^]^ Viral supernatants were generated as described previously. 1.5× 10^8^ of ID8/GC cells were transduced with pooled library lentivirus at low multiplicity of infection (MOI; ≈0.15‐0.2) and cultured with growth medium in the presence of 2 µg/ml puromycin to select gRNA‐transduced cells. One week after selection, pooled gRNA‐transduced tumor cells were washed and seeded in cell culture dishes for overnight. For T cell‐treated groups, cultured Pmel T cells were added at an effector: target (ET) ratio of 0.2:1 and 1:1 for 16 h. For non‐T cell treated groups, an equal amount of T cell growth medium was added. After treatment, nonadhesive cells, including T cells and dead tumor cells were removed by repeated washes using prewarmed PBS. Adherent cells were harvested by using 0.25% Trypsin‐EDTA (315050065, ThermoFisher Scientific) and washed three times using PBS. Additionally, tumor cells after 48‐h puromycin selection were collected to determine the coverage of the genome‐wide library in transduced cells before the functional immune screen. At least three replicates were performed for all treatment groups, and each sample includes at least 3 × 10^7^ of transduced cells for genomic DNA isolation using Blood & Cell Culture DNA MAXI Kit (#13323, Qiagen, Germantown, MD) as described by the manufacturer's protocol.

### Generating In Vivo ICB Sub‐Library

The oligo array containing gRNAs for the in vivo sub‐library was fully synthesized by GenScript Biotech Corporation (GenTitan Oligo Pools, Piscataway, NJ). Synthesized oligo array was eluted with nuclease‐free water, and 50 ng of oligos was amplified by the KAPA HiFi Hotstart (#07958935001, Roche, Pleasanton, CA) at 56 °C for annealing temperature using primers listed in Table S9 (Supporting Information). The PCR products were purified by using NucleoSpin Gel and PCR Clean‐up Kit (#740 609, Takara), then ligated with pKLV2 vector via the Gibson assembly according to the manufactory instruction. Gibson products were purified and transformed into Endura electrocompetent cells (#60242‐2, Lucigen, Middleton, WI) via a Bio‐Rad Micropulsar Electroporator. Following electroporation, electrocompetent cells were plated onto 245 mm Square BioAssay Dishes (#07‐200‐600, Corning Incorporated, Kennebunk, ME) with agar containing 100 µg mL^−1^ of carbenicillin (#C2130, Teknova, Hollister, CA) and incubated overnight at 32 °C. After 16 h of growth, all electrocompetent cells were collected from the plate for plasmid isolation using NucleoBond Xtra Midi Kits (3740420.1, Takara).

### In Vivo Immune Screen by Using ICB Sub‐Library

After construction of the library, ID8/GC cells were transduced to express the pooled in vivo sub‐library. Briefly, to generate lentiviral supernatants, HEK293T cells were seeded and transfected with 9 µg of lentiviral vectors encoding gRNAs library, along with lentiviral packaging plasmids (3 µg of pCMV‐VSV‐G and 4.5 µg of psPAX2) by the jetPRIME transfection reagent according to the manufacturer's protocol. Viral supernatants were collected 72 h after transfection and filtered through 0.45 µm Syringe Filter Unit to remove cell debris. 1.2 × 10^7^ ID8/GC cells were transduced with the pooled lentiviral supernatant at a low MOI (≈0.15–0.2) and cultured with complete growth media for 48 h. Then, transduced ID8/GC cells were selected by 2 µg mL^−1^ of puromycin for 2 days. Additionally, 3.0 × 10^7^ per sample of sub‐library expressing ID8/GC (ID8/GCSL) were collected and served as reference samples.

After 7 days of puromycin selection and expansion, ID8/GCSL cells were collected and subcutaneously inoculated into C57BL/6 and NSG mice at 1.0 × 10^7^ cells per injection into the posterior‐lateral flank of the mice. Five days after tumor inoculation, tumor‐bearing C57BL/6 mice were randomized into two groups to receive anti‐PD‐1 (clone: 29F.1A12; BE0146, BioxCell, Lebanon, NH) treatment or control (*n* = 5–10). Intraperitoneal injections of anti‐PD‐1 at 100 µg per dose, every other day, were used to treat the mice in anti‐PD‐1 group. NSG mice bearing pooled tumor cells served as a control to eliminate the genes with essential roles in controlling in vivo tumor development. Additionally, tumor size was monitored every other day by recording perpendicular diameters. At 10‐day treatment, tumors were collected to extract genomic DNA as described above.

### Next Generation Sequencing (NSG) for Samples From In Vitro and In Vivo Screens

The gRNA region in genomic DNA samples from in vitro and in vivo screens was amplified by two rounds of nested PCR reactions using KAPA HIFI HotStart as described previously.^[^ ^5^ ^]^ PCR products were then purified utilizing NucleoSpin Gel and PCR Clean‐up Kit, followed by a second round of amplification using barcoded primers. The quality and concentration of PCR products were determined by the Qubit ssDNA high sensitivity assay kit (#Q10212, Thermofisher Scientific) and the 2100 Bioanalyzer High Sensitivity DNA Kit (#5067‐46262100, Agilent Technologies, Santa Clara, CA), respectively. The frequency of each gRNA in the collected genomic samples was evaluated by NGS, utilizing the Illumina NextSeq550 sequencer at MD Anderson Cancer Center Advanced Technology Genomics Core.

### ICB Clinical Datasets

Clinical data from the phase II trial (NCT03026062), an investigator‐initiated trial to evaluate the effectiveness of durvalumab (anti‐PD‐L1) and tremelimumab (anti‐CTLA‐4) treatments in recurrent or refractory ovarian cancer at MD Anderson Cancer Center, were used in the concordance analysis. Detailed information on patient enrollment criteria, treatment schedule, and trial design was previously reported.^[^ ^75^, ^76^ ^]^ The study was conducted in accordance with the Declaration of Helsinki. The content of the informed consent forms was discussed with the subjects at the time of study entry, and all subjects gave their verbal and written consent to participate. The baseline tumor samples before treatment and the on‐treat samples from biopsied tumors immediately collected prior to the initiation of cycle 3 of treatment were used for the reported transcriptomic profiling analysis. MDACC Institutional Review Board approved the research protocols for this investigation.

### Cytotoxicity Assays

A set of genetically modified ID8/GC cell lines (1.5 × 10^5^ cells per well) was plated into 6‐well plates. The next day, 7‐day cultured Pmel T cells were co‐incubated with ID8/GC‐derived cells at an ET ratio of 0.2:1 overnight. Similarly, a set of genetically modified SKOV3/C cell lines (2 × 10^5^ cells per well) was plated into 6‐well plates. The next day, the 6‐day cultured hB7H3‐CAR‐T cells were co‐incubated with SKOV3/C‐derived cells at an ET ratio of 1:1 overnight. Then, dead cells and T cells were gently washed with prewarmed PBS twice. Adherent cells were harvested by using 0.25% Trypsin‐EDTA and stained with Trypan blue (#15250061, ThermoFisher Scientific) for manual cell counting. The killing efficiency was calculated as (1‐ number of live cells in T‐cell‐treated group/ number of live cells in the control group) ×100%.

### Assessment of Cytokine Production by T Cells

Equal numbers of cultured Pmel‐1 T cells were co‐cultured with murine OC cells with or without *Mettl5* expression in a 96‐well plate at an effector‐to‐target (ET) ratio of 1:1 for 24 h. The IFNγ concentrations in conditioned media were measured using the Mouse IFNγ Quantikine ELISA Kit (#MIF00‐1, R&D Systems) according to the manufacturer's instructions. All assays were performed in three biological replicates.

### Lipid Peroxidation Measurement

The lipid peroxidation levels in parental and genetically modified cells were determined by measuring malondialdehyde (MDA) using the Lipid Peroxidation assay kit (#ab118970, Abcam, Waltham, MA) according to the manufacturer's instructions. The MDA level was quantified fluorometrically at the wavelengths of Ex/Em = 532/553 nm and normalized by the corresponding protein concentration.

The lipid peroxidation levels in in vitro cultured tumor cells and in vivo tumor tissues were determined by measuring 4‐hydroxynonenal (4‐HNE) levels. For culture cells, tumor cells were seeded in Nunc Lab‐Tek II Chamber Slide System (#154 526, ThermoFisher Scientific) and fixed by Bouin's Fixative Solution (#1120‐1, Ricca Chemical Company, Arlington, TX) for 30 min at room temperature and then decolorized in 70% ethanol solution for 30 min. For tumor tissues, the tumor samples were dissected and postfixed in Bouin's Fixative Solution at 4 °C overnight and then decolorated in 70% ethanol solution for 5 days. After fixation, tumor samples were processed for paraffin sections (5 µm). Paraffin‐embedded sections (5 µm) were dewaxed in xylene and rehydrated in different concentrations of ethanol. Then, the slides were processed for antigen retrieval with 10 mM citrate buffer (pH 6.0) in a Lab Vision PT module (ThermoFisher Scientific) at 97 °C for 12 min. All slides were treated in a buffer composed of 50% (vol/vol) methanol and 0.3% (vol/vol) H_2_O_2_ for 30 min to quench endogenous peroxidase, and then were incubated with 3% (vol/vol) BSA in 1× PBS for 30 min to block unspecific binding. Sections were then incubated with anti‐4‐HNE (1:100; #ab46545, Abcam) at 4 °C overnight. After washing with PBS, slides were incubated with biotin‐labeled Goat anti‐Rabbit IgG (H+L) Secondary Antibody (1:200, #65‐6140, ThermoFisher Scientific) for 1 h at room temperature. Then, slides were incubated with VECTASTAIN ABC‐HRP reagent (PK‐4000, Vector lab, Newark, CA) for 1 h at room temperature and developed by 3, 3‐diaminobenzidine tetrahydrochloride as the chromogen. After counterstaining with hematoxylin, cells were dehydrated and mounted. The Keyence microscope (BZ‐X810, Itasca, IL) was used to take the images and count the cell numbers.

### Total RNA, Polyadenylated RNA and ribosome RNA Isolation

Total RNA was isolated from cells using TRIzol (#15596026, Invitrogen, Waltham, MA) according to the manufacturer's instructions. Polyadenylated RNA was isolated from total RNA by using the Dynabeads mRNA DIRECT Purification Kit (#61012, Thermofisher Scientific) according to the manufacturer instructions. rRNAs were isolated from the total RNA by running electrophoresis with 1.5% UltraPure Agarose in 1x RNase‐free TBE buffer for 30 min at 120 V. Related rRNA bands were isolated by using QIAquick Gel Extraction Kit (#28704, Qiagen).

### Liquid Chromatography–Mass Spectrometry (LC‐MS) for m^6^A Detection

RNA samples were prepared as previously described for LC‐MS analysis.^[^ ^58^ ^]^ Briefly, 30 ng of RNA was digested in 20 µL of P1 buffer (25 × 10^−3^ m NaCl, 2.5 × 10^−3^ m ZnCl_2_) containing 1 µL of Endonuclease P1 for 2 h at 42 °C. Subsequently, 1 µl of FastAP (#EF0651, Thermofisher Scientific) and 2.5 µL of 10x FastAP buffer were added to each sample and then incubated at 37 °C for 4 h. Samples were then diluted with an equal volume of water and filtered through a 0.2 µm PVDF filter (#SLGV004SL, Millipore–Sigma). 5 µL of each filtered sample was separated by reverse‐phase ultraperformance liquid chromatography on a ZORBAX Eclipse XDB‐C18 Rapid Resolution HT 2.1 × 50 mm, 1.8 µm column (Agilent) on an Agilent Technologies 1290 Infinity II liquid chromatography system, followed by mass spectrometry on a Sciex Triple Quad 6500 triple‐quadrupole mass spectrometer in positive electrospray ionization mode. Nucleosides were quantified using nucleoside‐to‐base transitions of 282.101 > 150.100 (m^6^A), 267.966 > 136.000 (A), and 284.004 > 152.100 (G). Standard curves were generated by injecting known concentrations of the corresponding pure nucleosides in the same run, and the percentage of modified to unmodified nucleosides was calculated based on calibrated concentrations.

### Immunoprecipitation of m^6^A RNAs

1 µg of total RNA from each sample had been used for the m^6^A immunoprecipitation. 5% of RNAs from each sample were served as input, and the residual RNAs were immunoprecipitated by using the EpiMark N6‐methyladenosine Enrichment Kit (#E1610S, New England Biolabs, Ipswich, MA) according to the manufacturer's instructions. Briefly, 1 µl of m^6^A antibody (provided in the above‐mentioned kit) per sample was coupled to prewashed Protein G Magnetic Beads (#S1430S, New England Biolabs) in the reaction buffer (150 mM NaCl, 10 mM Tris‐HCl pH 7.5, 0.1% NP‐40 in nuclease‐free water) with orbital rotation for 30 min at 4 °C. Then, beads were washed and incubated with fragmented RNAs in the reaction buffer for 3 h. Then, beads were washed with the reaction buffer twice, and sequentially washed with low‐salt reaction buffer (50 × 10^−3^ m NaCl, 10 × 10^−3^ m Tris–HCl pH 7.5, 0.1% NP‐40 in nuclease‐free water) for three times and high‐salt reaction buffer (500 × 10^−3^ m NaCl, 10 × 10^−3^ m Tris–HCl pH 7.5, 0.1% NP‐40 in nuclease‐free water) for three times, then resuspended in 30 µl of the RLT buffer (#79216, Qiagen) at room temperature. Eluates were purified using the RNA Clean & Concentrator‐5 kit (#R1013, Zymo Research, Irvine, CA). cDNA synthesis was performed within 20 µL reactions containing 4 µL Maxima H Minus cDNA Synthesis Master Mix (#M1661, ThermoFisher Scientific) at 25 °C for 10 min, 50 °C for 15 min, and 85 °C for 5 min. Synthesized cDNA was diluted into 100 µL using nuclease‐free water and used for real‐time PCR analysis. The real‐time PCR reactions were set up with 4.5 µL of diluted cDNA, 5 µL of FastStart Essential DNA Green Master mix (#06402712001, Roche), and 0.5 µl each of 10 × 10^−6^ forward and reverse primers listed in the Table S9. Real‐time PCR assays were performed on a LightCycler 96 (Roche) with the following settings: 95 °C, 300 s (preincubation); 95 °C 20 s, 60 °C 20 s, 72 °C 20 s (three‐step amplification). Analysis was performed with LightCycler 96 SW1.1 software (Roche).

### Profiling of Active Ribosomes

The profiling of active ribosomes was performed and measured by RiboLace as previously described.^[^ ^50^ ^]^ Briefly, tumor cells cultured at ≈80% confluent condition in T150 flasks were treated with 100 µg mL^−1^ of cycloheximide in the antibiotic‐free medium for 7 min at 37 °C. Treated cells were gently washed with prechilled PBS containing 100 µg mL^−1^ of cycloheximide twice, and cell pellets were collected by centrifugation at 950g for 5 min at 4 °C. Then, active ribosomes were isolated by using the RiboLace Starter Kit (RL00S‐04, Immagina BioTechnology, Pergine Valsugana, Italy) according to the manufacturer's instructions. Extracted RNAs were detected by running with Novex TBE‐Urea sample buffer (#LC6876, Invitrogen) on a 10% TBE‐Urea gel (#EC6875BOX, Invitrogen) in RNase‐free TBE buffer (#15581044, Invitrogen) together with the oligo marker provided in the kit for 1 h at 180 V. Then, the gel was stained with RNase‐free TBE buffer containing SYBR gold dye for 15 min and visualized by using the ChemiDoc Imaging System (Bio‐rad, Hercules, CA). Ribosome‐protected mRNA fragments (RPF) between ≈26 and 34 nucleotides were excised and purified by the small‐RNA PAGE recovery kit (#ZR1070, Zymo Research) according to the manufacturer's instructions. 22.5 µL RPF RNAs were end‐repaired by incubating with the buffer containing 3 µL T4 PNK (3M0201S, New England Biolabs), 3 µL PNK buffer (#B0201S, New England Biolabs), 1.5 µL RNase inhibitor (#AM2696, Invitrogen) at 37 °C for 60 min. Then, 1.5 µL 10 × 10^−3^ m ATP (#P0756S, New England Biolabs) and 1.5 µL T4 PNK were added to each reaction mixture, and an additional 60‐min incubation at 37 °C was performed. After the step of end repairing, samples were used to prepare libraries for sequencing by using the NEBNext Small RNA Library Prep Kit (#E7330S, New England Biolabs) according to the manufacturer's instructions. An Illumina NovaSeqX at The University of Chicago Genomics Facility was used to sequence processed samples under the setting of 100 bp single‐end reads.

### Fluorescent Reporter Assay to Evaluate Translation Efficiency of ATF4

Vectors of pSMALB‐ATF4.12 (Negative Control, #155033, Addgene), pSMALB‐ATF4.14 (Positive Control, #155034, Addgene), and pSMALB‐ATF4.5 (Native Reporter, #155032, Addgene), generous gifts from John Dick & Peter van Galen's lab, were used as the fluorescent reporters to evaluate ATF4 translation as previously described.^[^ ^77^ ^]^ Briefly, *METTL5*‐KO and control HEK293T cells were generated using lentiCRISPR V2 vector (#52961, Addgene) encoding *METTL5*‐specific gRNA or control gRNA, respectively. 5×10^5^ of *METTL5*‐KO and control HEK293T cells were seeded into one well of a 6‐well plate. After 24 h, 1 µg of the related reporter vector was transfected into a *METTL5*‐KO and control cells according to the manufacturer's protocol. For the groups with IFNγ treatment, transfected cells were treated with 5ng/ml of human IFN‐γ recombinant protein overnight. Fluorescent intensity in HEK293T cells was detected by using LSRFortessa X‐20 (BD Bioscience), and the mean value of calculated fluorescent intensity was calculated using BD FACSDiva Software.

### Chromatin Immunoprecipitation (ChIP)

To evaluate the occupancy of ATF4 at the promoter regions of *Slc7a11* and *Slc3a2*, tumor cells were harvested after crosslinking with 1% formaldehyde (#F8775, Sigma‐Aldrich). ChIP assay was performed using the ChIP Assay Kit (#17–295, Millipore‐Sigma) according to the manufacturer's protocol. Briefly, nuclei were isolated, and sonicated into ≈150 bp DNA fragments by using the Bioruptor Pico sonicator (Diagenode, Denville, NJ) with 14 cycles of sonication at the program of a 30 s‐on and 30 s‐off per cycle. Protein–DNA complexes were immunoprecipitated with either anti‐ATF4 (D4B8, 11 815, Cell Signaling Technology, 1:100) or normal rabbit IgG (12‐370, Millipore‐Sigma 1, 1:100), which served as the negative control. The protein‐DNA complexes were incubated with corresponding antibodies overnight at 4 °C. The complexes were then incubated with Pierce Protein A/G UltraLink Resin (#53133, ThermoFisher Scientific) for 1 h at 4 °C. Bound DNAs were eluted, reverse crosslinked, and purified using the PCR purification kit (#28106, Qiagen). 1% purified DNA fragments were used as the input template, real‐time PCR reactions were performed to determine the amount of bound DNAs by using SsoAdvanced Universal SYBR Green Supermix (#1725274, Bio‐Rad), and run on an ABI7500 (Applied Biosystems) according to the manufacturer's protocol. The primers used in the ChIP analysis were listed in the Table S9 (Supporting Information). The relative abundance was calculated relative to the input.

### Gene Expression Determined by Quantitative Reverse Transcription PCR (qRT‐PCR)

To determine GOI expression levels in tumor cells, total RNAs were isolated by using Direct‐zol RNA Miniprep Plus (#R2072, Zymo Research) according to the manufacturer's instructions. cDNA synthesis was performed using total RNA (500 ng) with random primers and iScript Reverse Transcription Supermix (#1708840, Bio‐Rad). qRT‐PCR was used to determine the levels of GOI mRNA. Triplicated PCR reactions by using SsoAdvanced Universal SYBR Green Supermix were run on an ABI 7500 (Applied Biosystems) according to the manufacturer's protocol. The gene expression level was normalized with the related genes, and the average 2^‐ΔΔCt^ value was calculated correspondingly. The fold changes relative to respective controls were determined. The primary information is provided in Table S9 (Supporting Information).

### Immunoblot Analysis

To verify the expression of GOIs, proteins were extracted from lysed cells using RIPA Lysis and Extraction Buffer (#89900; ThermoFisher Scientific), and the concentrations of protein samples were quantified with the Pierce BCA Protein Assay Kit (#23225, ThermoFisher Scientific). The western blot analysis was performed to determine the expression of GOI at the protein level. The intensity of specific protein bands was detected using the Immobilon Western Chemiluminescent HRP Substrate and visualized with the ChemiDoc Imaging System (Bio‐Rad). The intensity of specific bands was quantified by using Image Lab software (V5.2.1). The antibody targeting β‐actin (8H10D10, 33700) and ATF4 (D4B8, #11815) was purchased from the Cell Signaling Technology (Danvers, MA), human METTL5 (#16791‐1‐AP) was purchased from Proteintech Group (Rosemont, IL), mouse METTL5 (#A9217) was purchased from ABclonal (Woburn, MA) and the monoclonal ANTI‐FLAG antibody (M2, #F3165) was purchased from MilliporeSigma. HRP‐conjugated secondary antibodies anti‐rabbit IgG (#7047) and anti‐mouse IgG (#7076) were purchased from Cell Signaling Technology.

### In Vivo Murine Models

To evaluate the effect of *Mettl5* KO on in vivo tumor growth and sensitivity of tumor to anti‐PD‐1 treatment, 1 × 10^7^ of genetically modified ID8 cells in 100 µL of PBS were mixed with 100 µL of Cultrex Basement Membrane Extract (#3432‐010‐01, R&D systems) and then subcutaneously injected into one NSG or C57BL/6 mouse. Tumor‐bearing mice from the same expression line were randomized into two groups to receive anti‐PD‐1 or mock treatment. For the mice treated with anti‐PD‐1, three days after tumor inoculation, tumor‐bearing mice were intraperitoneally administered with anti‐PD‐1 (29F.1A12, #BE0273, BioxCell, Lebanon, NH) at 100 µg per dose, every other day. CD4⁺ and CD8⁺ T cell depletions were performed by using anti‐mouse CD4 (GK1.5, #BE0003, BioxCell) or CD8α (2.43, #BE0061, BioxCell) specific mAbs, intravenously injected (200 µg per mouse) on days –2 and 0 of tumor inoculation. Intraperitoneal injections were repeated every 6 days thereafter during the experiment to maintain CD4⁺ and CD8⁺ cell depletion as previously described.^[^ ^48^ ^]^Tumor sizes in all experimental mice were monitored by measuring the perpendicular diameters of tumors every other day. Tumor volume is calculated by ½ × length × width.^[^ ^2^ ^]^ All experiments were carried out in a blinded, randomized fashion.

### Immune Profiling of Tissue Samples

For flow cytometry analyses, paired spleen and tumor samples were harvested from tumor‐bearing mice after 13 days of tumor inoculation from ID8 tumor–bearing mice. Spleens and tumors were excised and placed in RPMI1640 medium with 10% FBS on ice until processing. Spleen tissues from experimental mice were mechanically disrupted into single‐cell suspensions, and Ammonium‐Chloride‐Potassium (ACK) lysis (#A1049201, ThermoFisher Scientific) was performed to remove red blood cells. Fresh tumor samples were processed into single‐cell suspensions for flow cytometry analysis. To prepare a single‐cell suspension, partial tumor samples were incubated in a triple‐enzyme solution: collagenase type IV (#C‐5138, Millipore‐Sigma), deoxyribonuclease type IV (#D‐5025, Millipore‐Sigma), and hyaluronidase type V (#H‐6254, Millipore‐Sigma) for 60 min at 37 °C, and then were mechanically disrupted into single‐cell suspensions. Single‐cell suspensions of splenocytes and isolated tumor cells were then pelleted, gently washed with PBS, and filtered through a 40 µm cell strainer (#352340, Corning Incorporated). Samples were incubated with BD Pharmingen Purified Rat Anti‐Mouse CD16/CD32 (#553142, BD Bioscience) for 15 min at room temperature and then incubated with a cocktail of antibodies targeting surface markers at 4 °C for 30 min. Cells were then fixed and permeabilized using either the Foxp3/transcription factor staining buffer set (#00–5523‐00, ThermoFisher Scientific) or the BD fixation/permeabilization solution kit (#554714, BD Biosciences) according to the manufacturer's protocols, and were incubated with a cocktail of antibodies against intracellular markers. The antibodies used for staining include anti‐CD4‐eFluor 450 (RM4‐5, #75–0042, TONBO Biosciences, San Diego, CA), anti‐CD8‐PE/Cy7 (53‐6.7, #60–0081, TONBO Biosciences, San Diego, CA), anti‐CD19‐APC (1D3, #20‐0193, TONBO Biosciences), anti‐CD25‐PerCP (PC61.5, #65–0251, TONBO Biosciences), anti‐Foxp3‐PE (FJK‐16s, #12–5773‐82, ThermoFisher Scientific), anit‐NK1.1‐APC (PK136, #550627, BD Biosciences), anti‐CD11b‐eFluor 450 (M1/70, #48–0112–82, eBioscience, San Diego, CA), anti‐CD11c‐APC (N418, #20–0114‐U100, TONBO Biosciences), anti‐F4/80‐PE (BM8.1, #50–4801, TONBO Biosciences), anti‐Ly6G‐PE/Cy7 (1A8, #60–1276, TONBO Biosciences), anti‐Ly6C‐PerCP‐Cy5.5 (HK1.4, #45‐5932‐80, eBioscience) and anti‐Ki67‐FITC (SolA15, #11–5698–82,eBioscience). An LSRFortessa X‐20 (BD Biosciences) cytometers were used for acquisition. Lymphocytes were gated by the combination of forward scatter and side scatter. CD8^+^ T cells were defined by CD8^+^. CD4^+^ T cells were defined by CD4^+^. B cells were defined by CD19^+^. Tregs were defined by CD4^+^ FoxP3^+^ CD25^+^. NK cells were defined by NK1.1^+^. Dendritic cells were defined by CD11b^−^ CD11c^+^. Macrophages were defined by CD11b^+^ F4/80^+^. Neutrophils were defined by CD11b^+^ Ly6G^+^. Immature Myeloid‐derived suppressor cells (MDSC) were defined by CD11b^+^ Ly6G^Low^ Ly6C^High^, and mature MDSCs were defined by CD11b^+^ Ly6G^Low^ Ly6C^Low^. Ki67 served as a marker for cell proliferation.

### Bioinformatics Analysis—In Vitro Genome‐Wide Immune Screen Analysis

The MAGeCK (v0.5.9.4) count module was used to calculate the read count of individual gRNAs in different samples with the following parameters: “‐l Mouse_v2_CRISPR.library –norm‐method total –sample‐label Ctrl, ET02, ET1 ‐n Mouse_v2_CRISPR_191205.count.txt –fastq files.fq.” MAGeCK test module was then applied with parameters “‐k Mouse_v2_CRISPR_191205.count.txt ‐c Ctrl, ET02, ET1 –norm‐method total –keep‐tmp ‐n Mouse_v2_CRISPR_191205 _ET_Ctrl –gene‐lfc‐method secondbest, ” to identify the genes whose gRNAs were significantly differentially presented between the control and T cell treatment groups at the cutoff using |Log_2_(fold‐change)| > 0.25 and *p* < 0.05. To identify tumor intrinsic immune factors by in vitro immune screens, genes were scored based on the performance of their corresponding gRNAs, ranging from 0 to 4. The in vitro score criteria were listed in the Figure S2A (Supporting Information).

### Integrative Analysis of In Vitro Screens and Transcriptomic Profiles of ICB Treated Patients for Generation of ICB Sublibrary

Transcriptomic data from 16 publicly available ICB‐treated cohorts, which are listed in the Table S3, Supporting Information, were extracted. To determine the association between gene expression and clinical outcome, either the Log_2_ (Fold change of gene expression) between responders and nonresponders or ‐ Log_2_ (Hazard Ratio of each gene) were used to calculate the weighted average across all cohorts as detailed in Figure S2B (Supporting Information). To keep both metrics on the same scale, the patient score was then normalized so that the absolute value of the 90^th^ percentile of scores was equal to 2. To integrate these clinical results with the previously mentioned genome‐wide in vitro immune screen results, merged scores for each gene were defined as the sum of the patient score and the in vitro score. The in vivo library was selected as genes having either the top 100 positive merged scores (enriched genes) or the top 400 negative merged scores (depleted genes), along with an additional 30 essential targets, 163 manually curated genes of interest, and 100 safe targets gRNAs served as the negative control.

### In Vivo Screen Analysis

For the results from the in vivo immune screen, the sequencing reads were mapped to the ICB sub‐library using bowtie (1.2.2) with parameters “–best –strata ‐a –norc ‐m 1 ‐5 6 –S.” Samtools (1.9) was used to calculate the read count of each gRNA. The fold change and statistical significance of genes among the between nonresponder and mock or responder and mock groups were calculated using a modification of the DrugZ algorithm.^[^ ^78^ ^]^ As the in vivo library was skewed towards depleted genes, two modifications to account for this asymmetry: i) normalization was performed relative to safe‐targeting gRNAs, and ii) final fold changes were not z‐normalized.

To integrate in vivo immune screen results with previous in vitro immune screen and patient data analysis, total scores of individual genes were defined as the sum of normalized merged scores and either normalized responder DrugZ score (if merged score < 0) or nonresponder DrugZ score (if merged score > 0), all to where the absolute value of the 90th percentile was 2.

### Concordance Analysis

Transcriptomic data and clinical data obtained from patients enrolled in the phase II trial (NCT03026062) were used in conjunction with our in vivo immune screen results to determine if results from in vivo screens and clinical results were concordant. Association of clinical benefit with each gene was assessed by overall survival, comparing patients with the upper quartile of expression versus the three lowest quartiles. We defined a gene as concordant if it was lower expression was associated with better survival and its corresponding gRNAs targeting the gene were depleted in the in vivo CRISPR immune screen, or if its higher expression was associated with better survival and gRNAs targeting the gene were enriched in the in vivo CRISPR immune screen. A binomial test was used to evaluate if the concordance is different than 50%.

### TCGA Dataset Analysis

RNA‐sequencing (RNA‐seq) data and clinical information in The Cancer Genome Atlas (TCGA, https://tcga‐data.nci.nih.gov) were extracted. The TCGA cohorts used for the reported analysis include Adrenocortical carcinoma (ACC); Bladder urothelial carcinoma (BLCA); Breast invasive carcinoma (BRCA); Cervical and endocervical cancers (CESC); Cholangiocarcinoma (CHOL); Colon adenocarcinoma (COAD); Lymphoid Neoplasm Diffuse Large B‐cell Lymphoma (DLBC); Esophageal carcinoma (ESCA); Glioblastoma multiforme (GBM); Head and Neck squamous cell carcinoma (HNSC); Kidney Chromophobe (KICH); Kidney renal clear cell carcinoma (KIRC); Kidney renal papillary cell carcinoma (KIRP); Acute Myeloid Leukemia (LAML); Brain Lower Grade Glioma (LGG); Liver hepatocellular carcinoma (LIHC); Lung adenocarcinoma (LUAD); Lung squamous cell carcinoma (LUSC); Mesothelioma (MESO); Ovarian cancer (OC); Pancreatic adenocarcinoma (PAAD); Pheochromocytoma and Paraganglioma (PCPG); Prostate adenocarcinoma (PRAD); Rectum adenocarcinoma (READ); Sarcoma (SARC); Skin Cutaneous Melanoma (SKCM); Stomach adenocarcinoma (STAD); Testicular Germ Cell Tumors (TGCT); Thyroid carcinoma (THCA); Thymoma (THYM); Uterine Corpus Endometrial Carcinoma (UCEC); Uterine Carcinosarcoma (UCS); Uveal Melanoma (UVM). In addition, triple‐negative breast cancer (TNBC) patients were stratified from the TCGA‐BRCA cohort. The recomputed RNA expression was obtained from the UCSC Toil RNA‐seq Recompute Compendium.^[^ ^79^ ^]^ The gene‐level of TPM values were Log_2_‐transformed with pseudo‐count 1 for further analysis. The intratumoral cytolytic activity score was calculated by taking the geometric mean of *GZMA* and *PRF1* TPM values. Patients in TCGA database were stratified into two groups based on the expression of *METTL5* (High means patients with the top 25% expression and low means the remaining 75% patients), and the overall survival (OS) between these two subsets of patients was analyzed.

### Comparison of Transcriptomic Profiles of Patients with Different *METTL5* Expressions

To further determine the association between high *METTL5* expression and immune‐resistant phenotype in ovarian cancer patients, we first generated two sets of gene signatures using the TONIC cohort. Based on the Log_2_FC (Responder‐Non‐responder) values of each gene, the top 100 upregulated and top 100 downregulated genes were selected to generate the responder and nonresponder gene sets, respectively. We further compared the transcriptomic changes in *METTL5*‐High (top 25%) versus *METTL5*‐low (remaining 75%) patients from the TCGA ovarian cancer (TCGA‐OV) cohort and the cohort of high‐grade serous ovarian cancer patients enrolled in the above‐mentioned trial of NCT03026062 (MDACC‐HGSOC). GSEA (4.2.1) was performed to evaluate whether transcriptomic changes associated with *METTL5* are enriched in the responder and nonresponder gene sets. Moreover, transcriptomic profiles of patients in the MDACC‐HGSOC cohort before ICB treatment (Pre), after ICB treatment (On), and their relative comparison (On‐Pre Ratio) were determined. GSEA by using hallmark gene sets was performed to determine the pathways differentially expressed between *METTL5*‐High and *METTL5*‐low patients. For each gene set, GSEA reports an enrichment score (ES) reflecting the degree to which a gene set is overrepresented at the top or the bottom of a list of genes ranked by correlation of the gene with the phenotype. GSEA walks down the ranked gene list, increasing the running‐sum statistic when a gene is in the gene set and decreasing it when it is not. The magnitude of the increment depends on the correlation coefficients. The ES is the maximum/minimum running sum encountered. Genes leading to the ES are considered core enrichment. To account for the sizes of gene sets, GSEA uses the Normalized Enrichment Score (NES), defined as the quotient of ES and the average ES of permuted cell type labels. A positive NES indicates enrichment of the gene sets in *METTL5*‐High patients, and a negative NES indicates enrichment of the gene sets in *METTL5*‐Low patients. A *p*‐value was also derived from the permutation. Benjamini‐Hochberg method was used to obtain adjusted *p*‐values (FDR).

### Comparison of Transcriptomic Profiles between Cells With and Without *METTL5* KO

For the transcriptome analysis of *METTL5*‐KO and negative control gRNA‐transduced ID8/GC and SKOV3‐Cas9 cell lines, RNAs were extracted from cells in triplicate using a Direct‐zol RNA Miniprep Plus kit (#R2071, Zymo Research). RNA‐seq libraries were prepared and subjected to paired‐end sequencing by Novogene. The raw sequencing reads were quantified using kallisto and then mapped to the mouse genome and transcriptome HiSAT2 2.1.0 for ID8‐derived data and human genome and transcriptome GRCh38 for SKOV3‐derived data as transcripts per million (TPM) for differential expression analysis. The normalization and differential gene expression were performed by edgeR [3.24.3]. Principal component analysis (PCA) was performed to determine the correlation between biological replicates within each individual KO line. Differentially expressed genes (DEGs) were defined by the ones whose expression level was significantly up‐ or downregulated in the KO lines when compared to the gNC control (|Log2 (fold‐change)| > 0.25 and *p* < 0.05).

QIAGEN Ingenuity Pathway Analysis (Version: 107193442) was used to select, annotate, and visualize genes by function and pathway. The DEGs with a cut‐off of *p*‐value < 0.05 and |Log_2_FC| > 0.25 were selected for the IPA analysis. IPA calculates a *p*‐value for each gene set using a Right‐Tailed Fisher's Exact Test to reflect the likelihood of the gene set and the DEGs being random. IPA analysis identified those canonical pathways differentially expressed (*p* < 0.05) between comparison groups.

### The Relationship of Translation Efficiency and the Strength of Kozak Sequence in mRNAs

To determine the impact of uORFs on mRNA translation efficiency in cells with and without METTL5, translation efficiencies (TE) were analyzed based on the strength of the Kozak sequence in various transcripts. TE were calculated with the change of translation obtained from RiboLace and the change of gene expression acquired from RNA‐seq. Specifically, TE = Log_2_(FC_RiboLace(_ *_METTL5_*_‐KO versus NC)_/FC_RNAseq(_ *_METTL5_*_‐KO versus NC)_). Kozak sequences in different transcripts from the Ribo‐uORF database were categorized as Weak, Moderate, or Strong as previously described.^[^ ^80^ ^]^ Based on the corresponding Kozak sequences, mRNA transcripts obtained from RiboLace and RNA‐seq analysis were assigned into four categories: “No” (no or only weak Kozak sequence), “Moderate” (at least one moderate Kozak sequence), “Strong” (one strong Kozak sequence), and “Supreme” (multiple strong Kozak sequences). Comparisons of translation efficiencies among different categories of mRNA transcripts were performed.

### Prediction of ATF4 Binding Sites on *Slc7a11* and *Slc3a2* Promoter Region

The PROMO virtual laboratory was used to identify the putative transcription factor binding sites (TFBS) of ATF4 on murine *Slc7a11* and *Slc3a2* genes as previously described.^[^ ^81^ ^]^ The *in silicon* validation of these binding sites within the promoter regions of the *Slc7a11* and *Slc3a2* genes was subsequently confirmed through the analysis of reported ChIP‐seq data (GEO access number GSE179958).^[^ ^82^ ^]^ The ATF4 binding sites identified by PROMO, overlapping with results from both antibody‐based ChIP‐seq analyses, were used for real‐time PCR primer design.

### Statistical Analysis

Data are presented as mean ± SEM. The sample sizes for each experiment, ranging from 3 to 6, were determined by the exploratory scope of the study and practical feasibility. Specific details are provided within the figure legends. Preprocessing of RNA‐seq data was indicated in the “Bioinformatics analysis” sub‐section. No data points are excluded as outliers. Assessments of differences in continuous measurements between the two groups were made using two‐sample t‐tests. Multiple group comparisons were performed by Analysis of Variance (ANOVA) with repeated measures. Chi‐squared tests were used to determine the statistical difference among categorical variables. The Kaplan‐Meier method and log‐rank test were used to compare survival between groups. All statistical analyses, with the exception of those performed for genetic screens, were conducted using two‐tailed tests. Graph generation statistical analyses were performed using the GraphPad Prism software program (GraphPad 9.0.2), Tableau software program (Tableau 8.2), and R software programming language (version 3.1.0). A *p*‐value of less than 0.05 is considered to be statistically significant. *p < 0.05; **p < 0.01; ***p < 0.001; ****p < 0.0001.

## Conflict of Interest

AA. Jazaeri reports personal consulting fees from Gerson Lehrman Group, Guidepoint, and paid advisory activities (last 2 years) for Iovance advisory board meeting, NuProbe, Simcere, PACT Pharma, Genentech‐Roche, Eisai, Agenus, and Macrogenics, Theolytics outside submitted work. He also reports grant funding to the institution for clinical trials from AstraZeneca, Bristol Myers Squibb (BMS), Iovance, Aravive, Pfizer, Immatics US, Eli Lilly, Merck, Macrogenics, and stock/stock options from Avenge Bio outside submitted work. C. He is a scientific founder, a member of the scientific advisory board, and equity holder of Aferna Bio, Inc. and Ellis Bio Inc., a scientific cofounder and equity holder of Accent Therapeutics, Inc., and a member of the scientific advisory board of Rona Therapeutics and Element Biosciences. M. Kok reports funding to the institute from BMS, Roche/Genentech, AstraZeneca (AZ), and an advisory role/speaker fee for Alderaan, BMS, Domain Therapeutics, Gilead, Roche, Merck Sharp & Dohme, and Daiichi Sankyo, outside the submitted work. No potential conflicts of interest were disclosed by other authors.

## Author Contributions

J.H., C.‐W.J., and N.A.E. contributed equally to this work. C H., A.J., and W.P. conceptualized and designed the study. J.H., C.J., N.E., L.S., N.Z., S.C., A.G., X.L., W.W., A.A., C.D., D.B., A.G., M.F., H.L., S.L., A.J., and W.P. acquired data (provided required animals, cells, patient samples, clinical information, etc.). C.J., Y.W., Y.W., M.D., T.Z., L.Z., M.K., L.W., Q.F., Y.C., D.M., N.S., and W.P. performed the analysis and interpretation of data (statistical analysis and bioinformatics analysis). J.H., C.J., N.E., X.M., D.M., N.S., C.H., A.J., and W.P. wrote and/or revised the manuscript. C.H., A.J., and W.P. supervised the study.

## Supporting information



Supporting Information

## Data Availability

The in vitro and in vivo immune screen data have been deposited and are publicly available at Gene Expression Omnibus (GEO) with the ID: GSE267517. The RNA‐seq data have been deposited and are publicly available at GEO with the ID: GSE267542. Any additional information required to reanalyze the data reported in this work paper is available from the lead contact upon request.
