# Supplementary material for: Tumor Intrinsic METTL5 Modulates ATF4 Translation to Prevent T Cell‐Induced Ferroptosis in Ovarian Cancer
Source: Adv Sci (Weinh). 2025 Oct 3;12(46):e07718. doi: 10.1002/advs.202507718 (PMC12697795; doi:10.1002/advs.202507718)
Supplement: Supplementary file 1 — Supporting Information [file ADVS-12-e07718-s001.pdf]

Supplementary information for

Tumor intrinsic METTL5 modulates ATF4 translation to prevent T cell-induced ferroptosis in ovarian cancer

Jiakai Hou[1,#], Cheng-Wei Ju[2,3,4,#], Nicholas A. Egan[1,#], Yanjun Wei[5], Yunfei Wang[6], Minghao Dang[7], Tianyi Zhou[1], Leilei Shi[8], Ningbo Zheng[1], Si Chen[1], Ashley M. Guerrero[1], Xiaofang Liang[1], Wanfu Wu[1], Areej Akhtar[1], Chitra Dhiman[1], Debanwita Roy Burman[1], Andro E. Gerges[1], Mason D. Flores[1], Han Li[2,3], Li-Sheng Zhang[2], Marleen Kok[9,10], Xiaobo Mao[11], Linghua Wang[12], Qin Feng[1], Yiwen Chen[5,13], Sanghoon Lee[14], Daniel J. McGrail[15,18], Nidhi Sahni[5,8,18], Chuan He[2,3,16,17,18,*], Amir A. Jazaeri[14,18,*], and Weiyi Peng[1,18,*]

[1]Department of Biology and Biochemistry, University of Houston, Houston, TX, USA.

[2]Department of Chemistry, The University of Chicago, Chicago, IL, USA.

[3]Howard Hughes Medical Institute, The University of Chicago, Chicago, IL, USA.

[4]Pritzker School of Molecular Engineering, The University of Chicago, Chicago, IL, USA.

[5]Department of Bioinformatics and Computational Biology, The University of Texas MD Anderson Cancer Center, Houston, TX, USA.

[6]Clinical Science Lab, H. Lee Moffitt Cancer Center & Research Institute, Tampa, FL, USA.

[7]Department of Lymphoma and Myeloma, The University of Texas MD Anderson Cancer Center, Houston, TX, USA.

[8]Department of Epigenetics and Molecular Carcinogenesis, The University of Texas MD Anderson Cancer Center, Houston, TX, USA.

[9]Division of Tumor Biology & Immunology, The Netherlands Cancer Institute, Amsterdam, The Netherlands.

[10]Department of Medical Oncology, The Netherlands Cancer Institute, Amsterdam, The Netherlands.

[11]Neuroregeneration and Stem Cell Programs, Institute for Cell Engineering, Department of Neurology, Johns Hopkins University School of Medicine, Baltimore, MD, USA.

[12]Department of Genomic Medicine, The University of Texas MD Anderson Cancer Center, Houston, TX, USA.

[13]Quantitative Sciences Program, The University of Texas MD Anderson Cancer Center, UT Health Graduate School of Biomedical Sciences, Houston, TX, USA.

[14]Department of Gynecologic Oncology and Reproductive Medicine, Division of Surgery, The University of Texas MD Anderson Cancer Center, Houston, TX, USA.

[15]Center for Immunotherapy and Precision Immuno-Oncology, Cleveland Clinic, Cleveland, OH, USA.

[16]Department of Biochemistry and Molecular Biology, The University of Chicago, Chicago, IL, USA

[17]Institute for Biophysical Dynamics, The University of Chicago, Chicago, IL, USA

[18]Co-senior authors.

[#]These authors contributed equally.

***Correspondence:** chuanhe@uchicago.edu (H.C.), aajazaeri@mdanderson.org (A.J.), wpeng2@central.uh.edu (W.P.)

Lead Contact: Weiyi Peng, University of Houston, 3455 Cullen Blvd., Suite 342 Houston, TX 77204-5001, Phone: 713-743-6941, E-mail: wpeng2@Central.uh.edu

Supplemental information includes

- **Supplementary Figure S1-11**
- **Supplementary Table S1-9**

Supplementary Figure S1

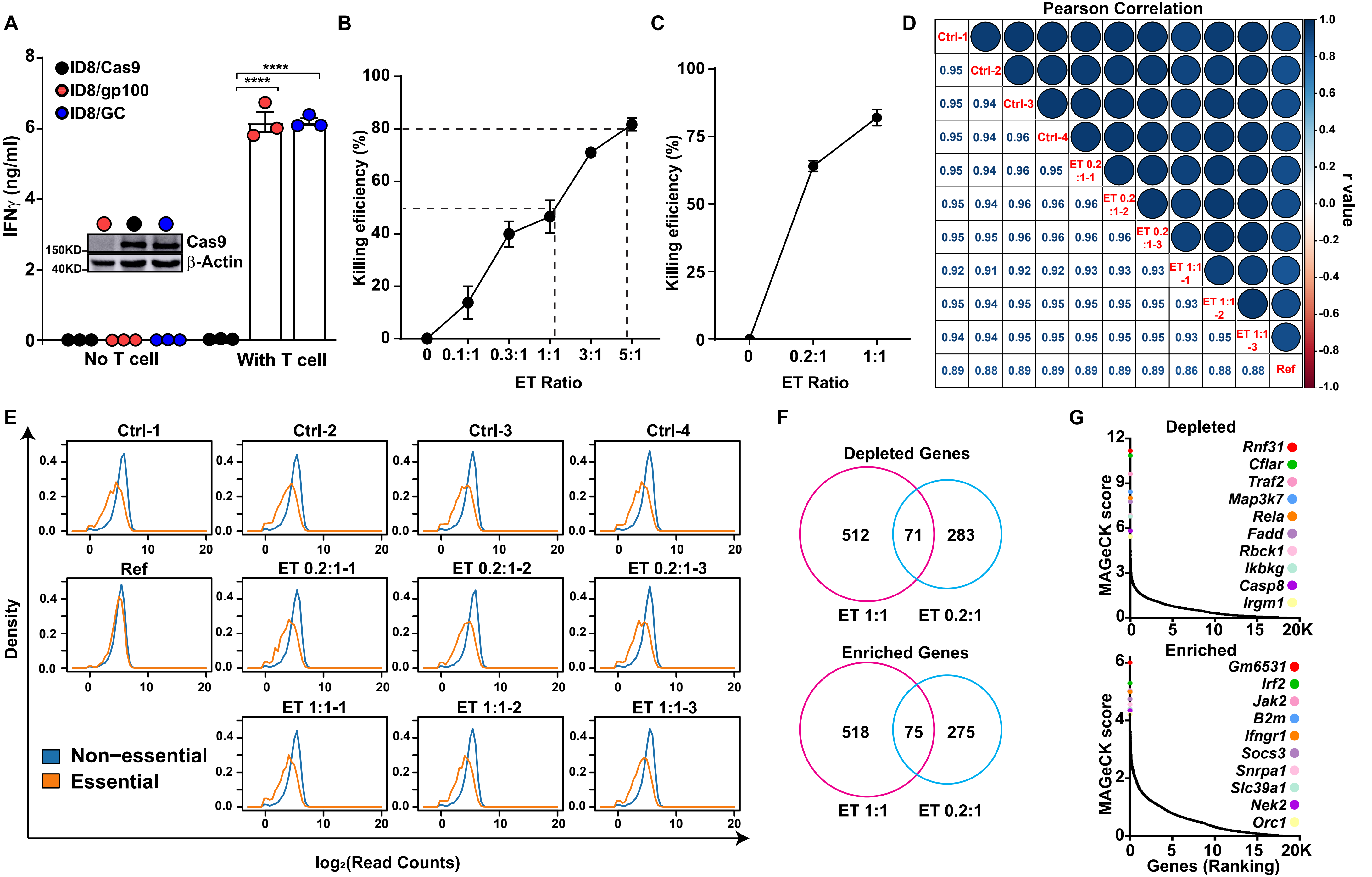

Supplementary Figure S1. Discovery of tumor-intrinsic factors regulating ovarian cancer cell sensitivity to T cell-mediated killing via *in vitro* genome-wide immune screen. **(A)** Expression of gp100 and Cas9 in ID8 derived cell lines. Expression of FLAG tagged Cas9 among ID8 derived cell lines was confirmed by western blot. Expression of gp100 was assessed by Pmel T cell recognition. IFNγ levels in the conditioned media resulting from the co-culture of ID8 cells with Pmel T cells were quantified using ELISA. Data were analyzed using one-way ANOVA followed by Dunnett's post hoc test and were presented as mean values ± SEM (n = 3). **(B)** *In vitro* killing efficiency of ID8/GC by Pmel T cells. ID8/GC cells were co-cultured with Pmel T cells at indicated ET ratios overnight. The cell viability was measured and calculated by using trypan blue staining. Data were presented as mean values ± SEM (n = 3). **(C)** Killing efficiency of ID8/GCSL cells observed in *in vitro* genome-wide immune screens. Pooled gRNA library-expressing ID8/GC cells were co-cultured with Pmel T cells overnight at ET ratios of 0.2:1 and 1:1. Cell viability was measured and calculated by using trypan blue staining. Data were presented as mean values ± SEM (n = 3). **(D)** Correlation of gRNA abundance across experimental samples. Pairwise Pearson correlation analyses were performed, and r values were calculated and tabulated. **(E)** Distribution of gRNAs targeting essential and non-essential genes among the control samples (upper panel), the reference sample (middle panel), the ET 0.2:1 samples (middle panel) and the ET 1:1 samples (bottom panel). **(F)** Venn diagram of overlapping candidate genes identified from *in vitro* genome-wide immune screens under different ET ratios. The number of genes with significantly depleted (upper panel) or enriched (lower panel) gRNAs ($|Log_2(\text{fold change})| > 0.25$, $p < 0.05$) at ET ratios of 0.2:1 (blue) and/or 1:1 (red) is shown. **(g)** MAGeCK scores of candidate genes identified from *in vitro* genome-wide immune screens at ET ratio of 0.2:1. Based on MAGeCK scores, the top 10 depleted (upper panel) and enriched (lower panel) genes are highlighted.

A

- **For enriched genes**

Score	Definition
4	FDR<0.25 for both screen conditions
3	*p*<0.05 for both screen conditions
2	*p*<0.05 for one condition *p*<0.25 for the other
1	*p*<0.25 at both screen conditions

- **For depleted genes**

Score	Definition
-4	FDR<0.25 for both screen conditions
-3	*p*<0.05 for both screen conditions
-2	*p*<0.05 for one condition *p*<0.25 for the other
-1	*p*<0.25 at both screen conditions

B

Type	#	Cohort Name	Weight(%)	
SKCM	1	Riaz	6.25	2.08
	2	Gide (Mono)		1.04
	3	Hugo		2.08
	4	Gide (Dual)		1.04
TNBC	5	TONIC	25.00	25.00
KIRC	6	CheckMate025	25.00	3.57
	7	Ascierto		3.57
	8	Miao Archival		3.57
	9	Miao NCT0135872		3.57
	10	IMmotion150		7.14
	11	CheckMate010		3.57
BLCA	12	IMvigor210	12.50	6.25
	13	Snyder		6.25
STAD	14	Kim	12.50	12.50
GBM	15	Cloughesy	18.75	9.38
	16	Zhao		9.38
			Total	100

Supplementary Figure S2. Calculations of *in vitro* score and patient scores. (A) *In vitro* scores were determined based on results from *in vitro* genome-wide immune screen results. **(B)** Patient scores were derived from data collected in the reported ICB cohorts. Either the Log_2(Fold change of gene expression) between responders and non-responders or $-Log_2$(Hazard Ratio of each gene) were used to calculate. These values were then used to compute a weighted average across the ICB cohorts.

A

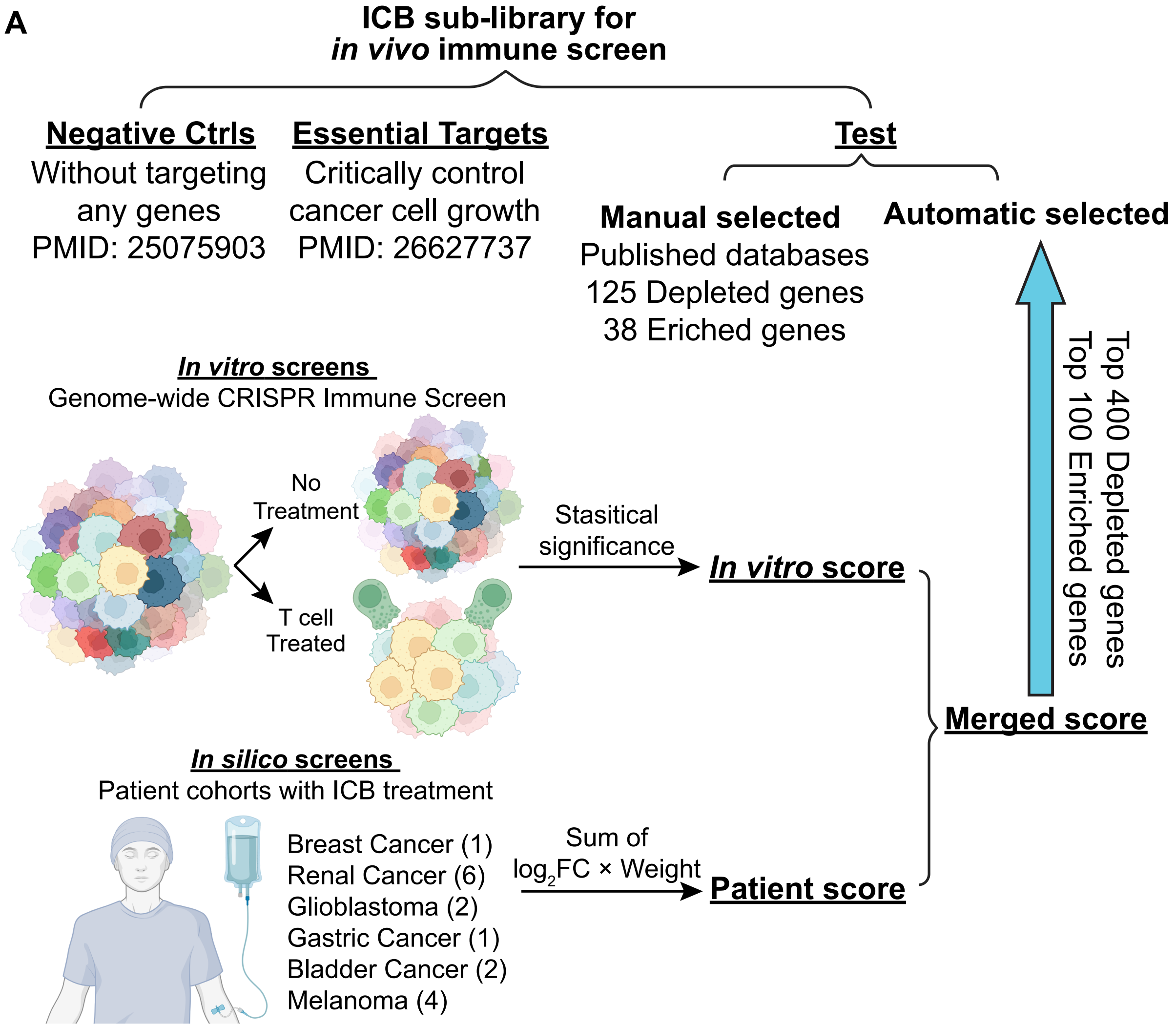

B

ICB sub-library for *in vivo* immune screen

		Percentage of gRNAs	Number of genes (gRNAs)
	Negative Ctrls	3%	N/A (100)
	Essential Targets	4%	30 (147)
GOIs	Depleted genes	74%	525 (2596)
	Enriched genes	19%	138 (684)
	Total	100%	693 (3527)

C D

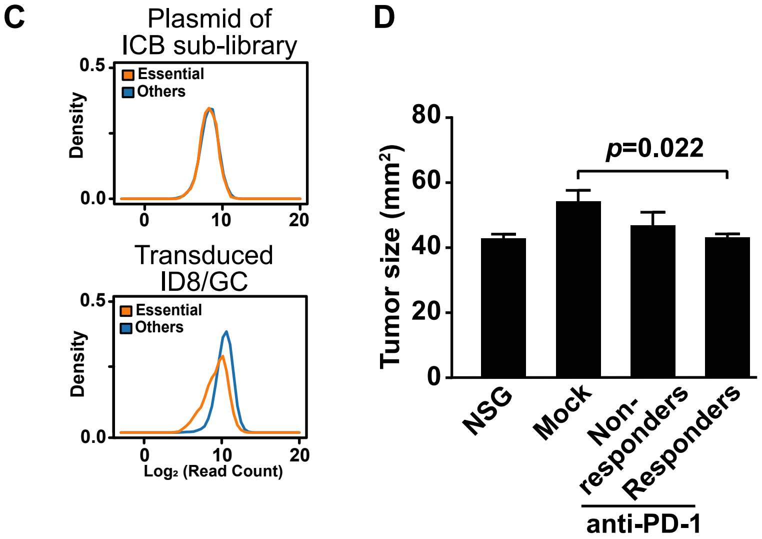

E

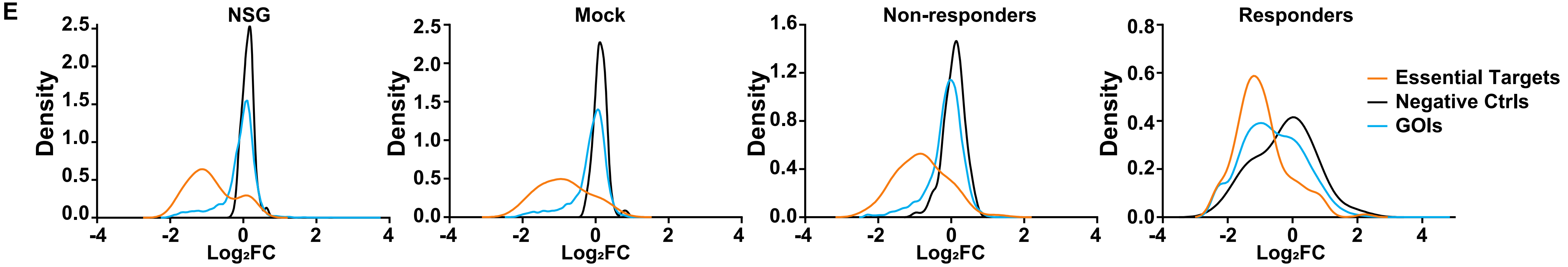

Supplementary Figure S3. Establishment of ICB sub-library for *in vivo* ICB screen. (A) Workflow diagram depicting the integrative strategy for candidate selection. Selection criteria included the *in vitro* score from the *in vitro* genome-wide immune screens, patient score derived from reported ICB cohorts, other reported candidates obtained from previous publications, and quality controls. The numbers of genes and gRNAs included in each category of the ICB sub-library for *in vivo* ICB screen are listed. **(B)** Components of the murine ICB sub-library used for *in vivo* ICB screens. gRNAs targeting OC immune factors (gene-of-interest, GOIs), essential genes (Essential Targets), and non-targetable controls (Negative Ctrls) were included. Each gene was represented by 2-5 distinct gRNAs. Numbers of gRNAs and targeted genes for each category are listed. **(C)** Density distribution of gRNAs in the essential category (Essential) versus in the remaining categories (Others). Distributions are shown for pooled vectors encoding the constructed ICB sub-library library (upper panel) and pooled tumor cells transduced with the gRNA library after 7-day expansion under puromycin selection (bottom panel). **(D)** Tumor sizes in tumor-bearing mice for each group. Measurements were recorded on the day for tumor samples collection (15 days after tumor inoculation). Data were presented as mean values ± SEM. Statistical comparisons between anti-PD-1-treated and mock groups are provided, with exact *p*-values indicating significance (n = 5). **(E)** Changes of gRNA distribution across experimental groups. Density and Log_2 fold change (FC) of gRNAs were plotted.

Supplementary Figure S4

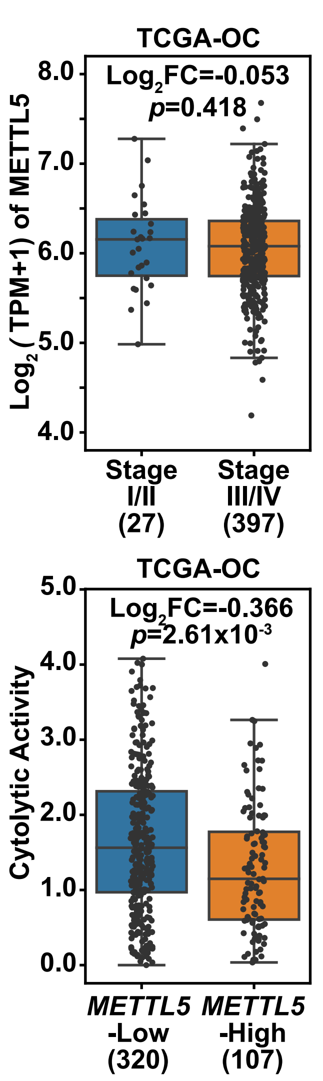

C

	TCGA-OC	MDACC-HGSOC
T cells	-0.0555	0.0244
CTLs	-0.0775	-0.114
B cells	-0.0954	-0.131
NK cells	-0.106	-0.173

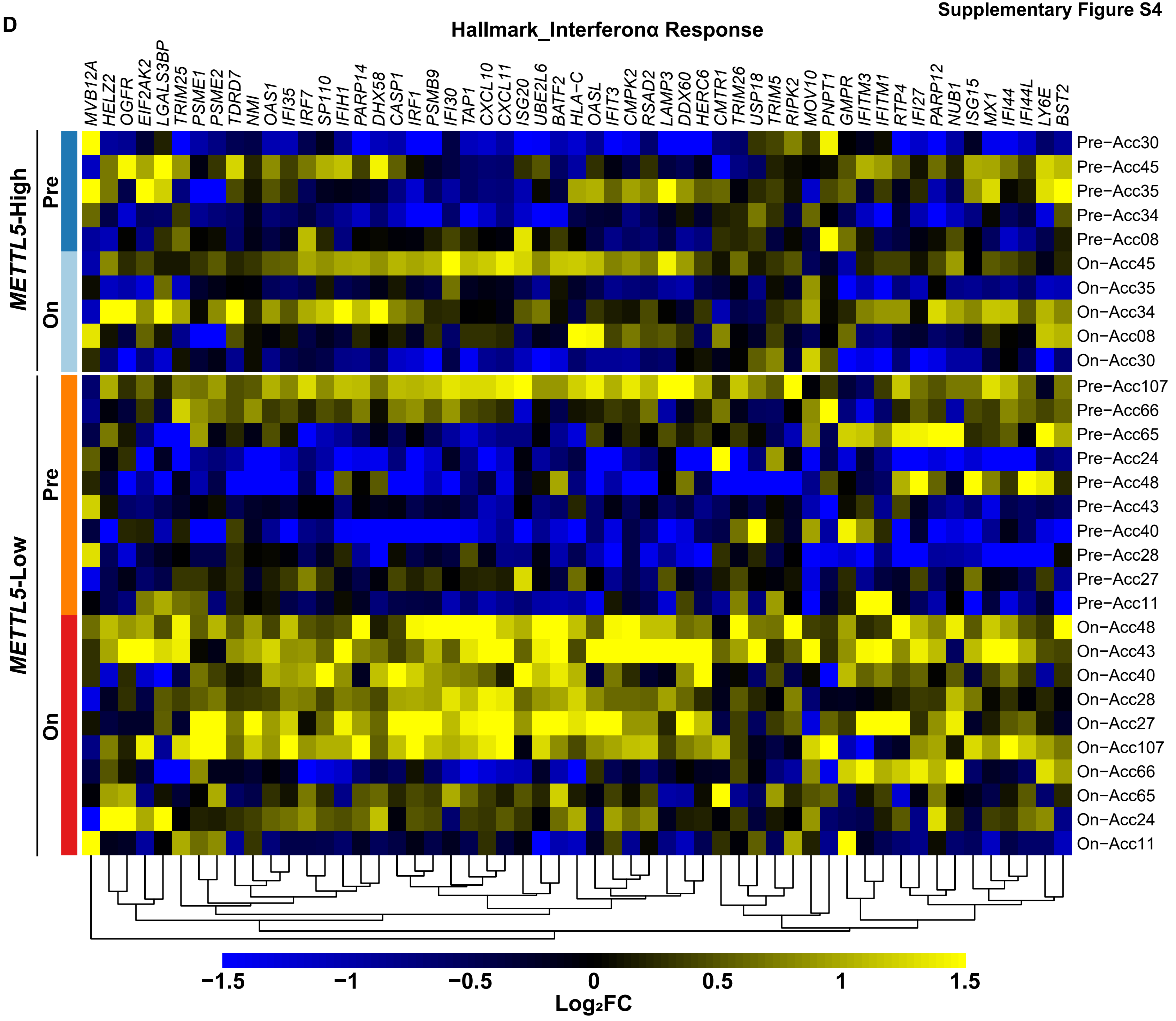

Supplementary Figure S4. ***METTL5*** **expression negatively correlates with patient response to ICB. (A)** Boxplot illustrating *METTL5* expression levels in OC patients across different diseases stages. Data were presented as median values ± 1.5×Interquartile Range (IQR). Statistical comparisons between Stage I/II and Stage III/IV are provided, with exact *p*-values indicating significance. **(B)** Boxplot comparing cytolytic activity scores between *METTL5*-High and *METTL5*-Low OC patients. Data were presented as median values ± 1.5×IQR. Statistical comparisons between *METTL5*-High and *METTL5*-Low OC patients are provided, with exact *p*-values indicating significance. **(C)** Correlations between immune signature scores and *METTL5* expression. For each comparison, r values are shown within the respective immune cells. None of the observed correlations reached statistical significance. **(D)** Patients in the MDACC-HGSOC cohort were stratified into *METTL5*-High and *METTL5*-Low groups based on baseline tumor *METTL5* expression. Transcriptomic profiles between *METTL5*-High and *METTL5*-Low groups were compared, and DEGs in the IFNα response pathway were identified. Relative expression levels of selected genes in baseline (Pre) and on-treatment (On) tumors are illustrated in a heatmap.

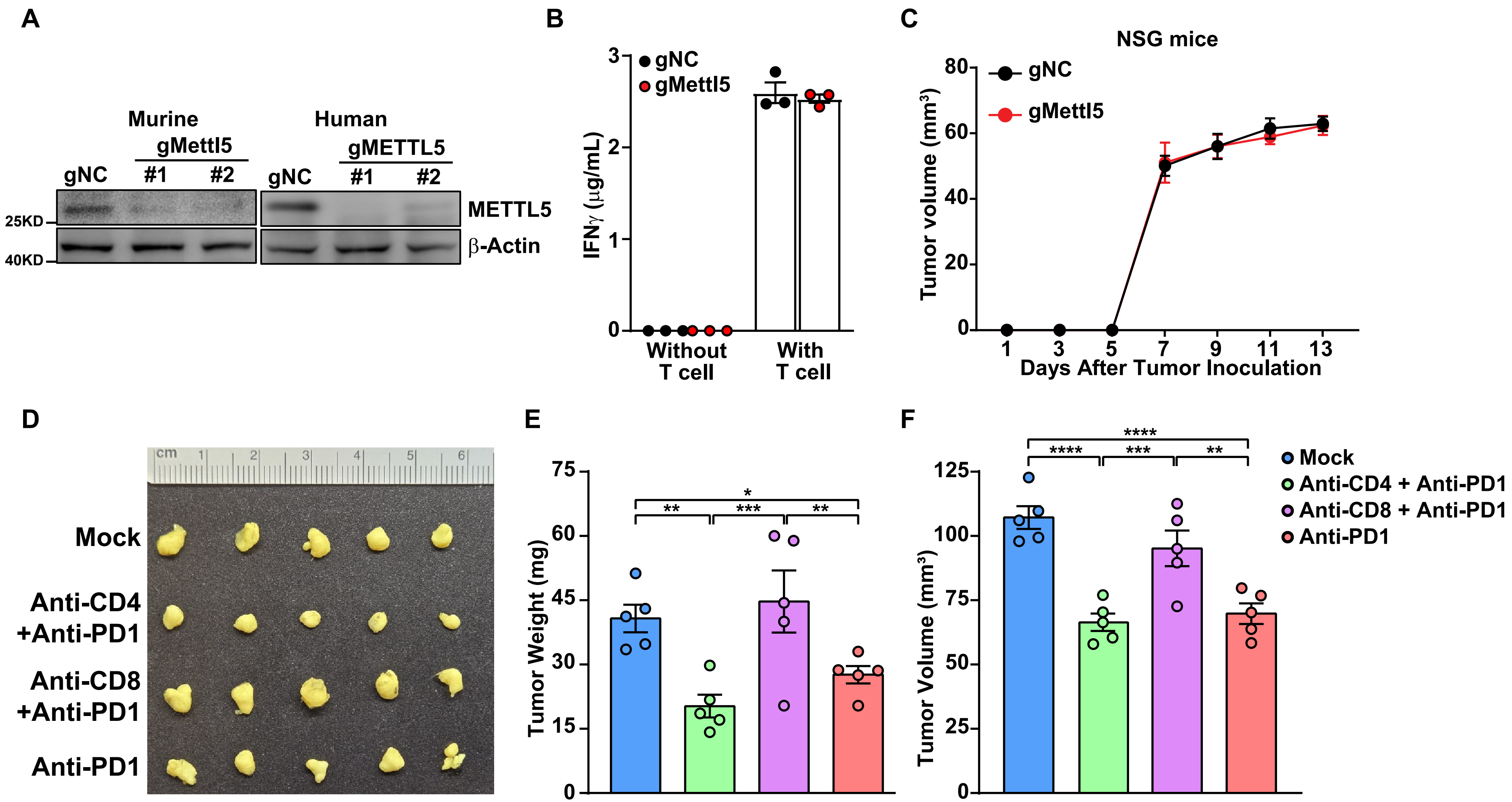
A
Murine
gMettl5
gNC
#1
#2
Human
gMETTL5
gNC
#1
#2
25KD
40KD
METTL5
β-Actin
B
gNC
gMettl5
IFNγ (μg/mL)
Without T cell
With T cell
C
NSG mice
gNC
gMettl5
Tumor volume (mm³)
Days After Tumor Inoculation
D
cm
Mock
Anti-CD4 +Anti-PD1
Anti-CD8 +Anti-PD1
Anti-PD1
E
Tumor Weight (mg)
F
Tumor Volume (mm³)
Mock
Anti-CD4 + Anti-PD1
Anti-CD8 + Anti-PD1
Anti-PD1

Supplementary Figure S5. Molecular and phenotypic changes of *METTL5* KO tumors. (A) Validation of *METTL5* genetic depletion in both murine and human ovarian cancer cell lines. *METTL5*-KO (gMettl5) ID8/GC and SKOV3/C cell lines were generated by using two independent gRNAs. Cells expressing non-targetable gRNA (gNC) were served as controls. The knockout efficiency of *METTL5* was confirmed via western blot. **(B)** Cytokine production of tumor-reactive T cells after stimulation of *Mettl5*-KO and control tumor cells. Equal numbers of Pmel T cells were co-cultured with *Mettl5*-KO and control ID8/GC cells for 24 hours. Conditioned media were collected, and IFNγ concentrations were quantified using the ELISA. Data are presented as mean ± SEM (n = 3), and statistical analysis was performed using one-way ANOVA followed by Dunnett's post hoc test. **(C)** Genetic depletion of *METTL5* does not affect tumor growth in NSG mice. *METTL5*-KO and control ID8/GC cells were inoculated into NSG mice. Tumor size was measured every two days starting with five days post-inoculation. Data were analyzed using two-way ANOVA followed by the multiple comparison test and were presented as mean values ± SEM (n = 4). **(D-F)** Anti-PD1 effect in *Mettl5*-KO tumors is primarily dependent on $CD8^+$ T cell. *Mettl5*-KO ID8/GC cells were subcutaneously inoculated into C57BL/6 mice. Three days after tumor inoculation, mice were treated intraperitoneally with either PBS or anti–PD-1 antibody (100 μg per dose) every other day. $CD4^+$and $CD8^+$ T cell depletions were performed by using anti-mouse CD4 or CD8α specific mAbs, intravenously injected (200 μg per dose). 10 days after the initiation of anti–PD-1 treatment, tumors were collected and imagined **(D)**. Tumor weights **(E)** and volumes **(F)** were recorded (n = 5). Data were presented as mean values ± SEM and analyzed using one-way ANOVA followed by Dunnett's post hoc test (n = 5). $*p < 0.05$, $**p < 0.01$, $***\ p < 0.01$ and $****p < 0.0001$.

Supplementary Figure S6

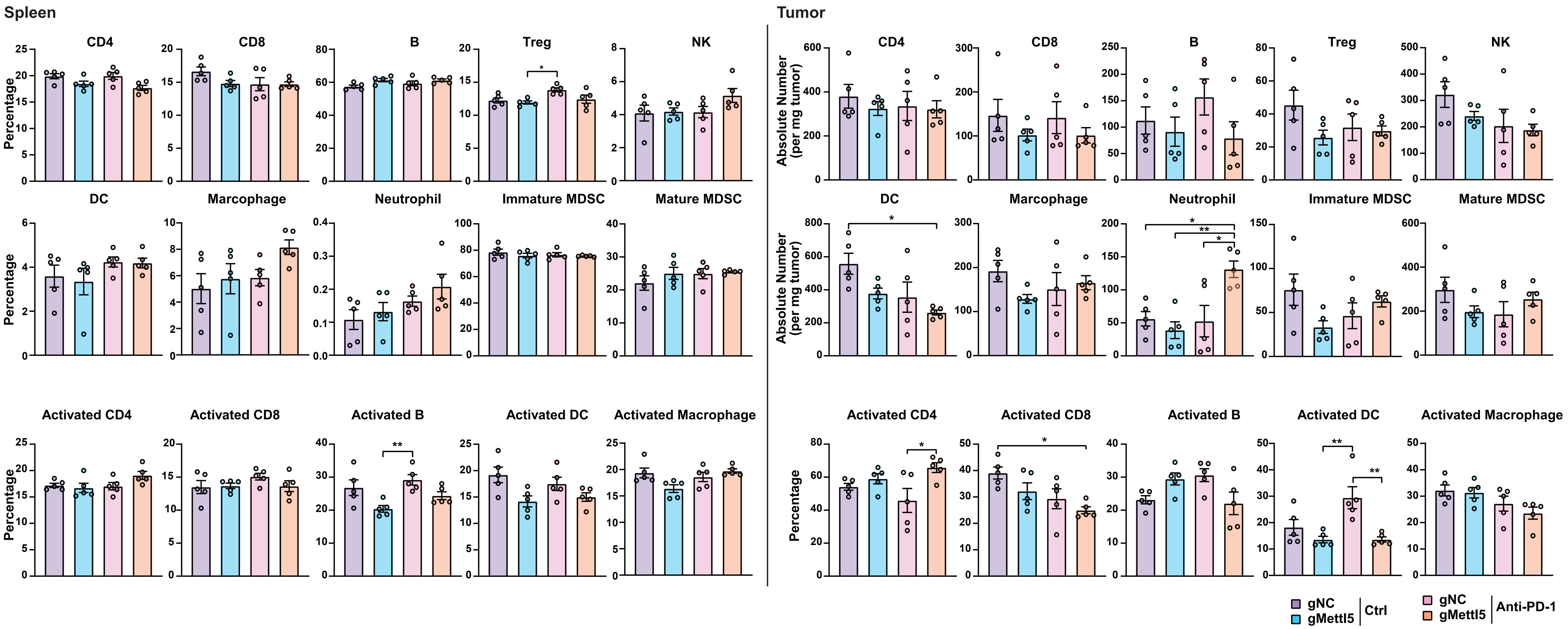

Supplemental Figure S6: Immune profiling of mice bearing METTL5 KO or control tumors. Abundancy and proliferation of immune cells in paired spleen and tumor tissue samples from OC bearing mice receiving anti-PD1 treatment. ID8/GC transduced with *Mettl5*-specific gRNAs (gMettl5) were inoculated into C57BL/6 mice. Mice-bearing tumors expressing non-targetable gRNA (gNC) were used as negative control. Three days after tumor inoculation, mice with measurable tumors were randomized and treated intraperitoneally with either PBS or anti-PD-1 (100 μg per dose) every other day. Spleen (Left panel) and tumor tissues (Right panel) were collected on day 10 after anti-PD1 treatments for flow cytometry analysis (n=5 per group). Data were analyzed using one-way ANOVA followed by Dunnett's post hoc test and were presented as mean values ± SEM (n = 5). *$p < 0.05$, **$p < 0.01$.

Supplementary Figure S7

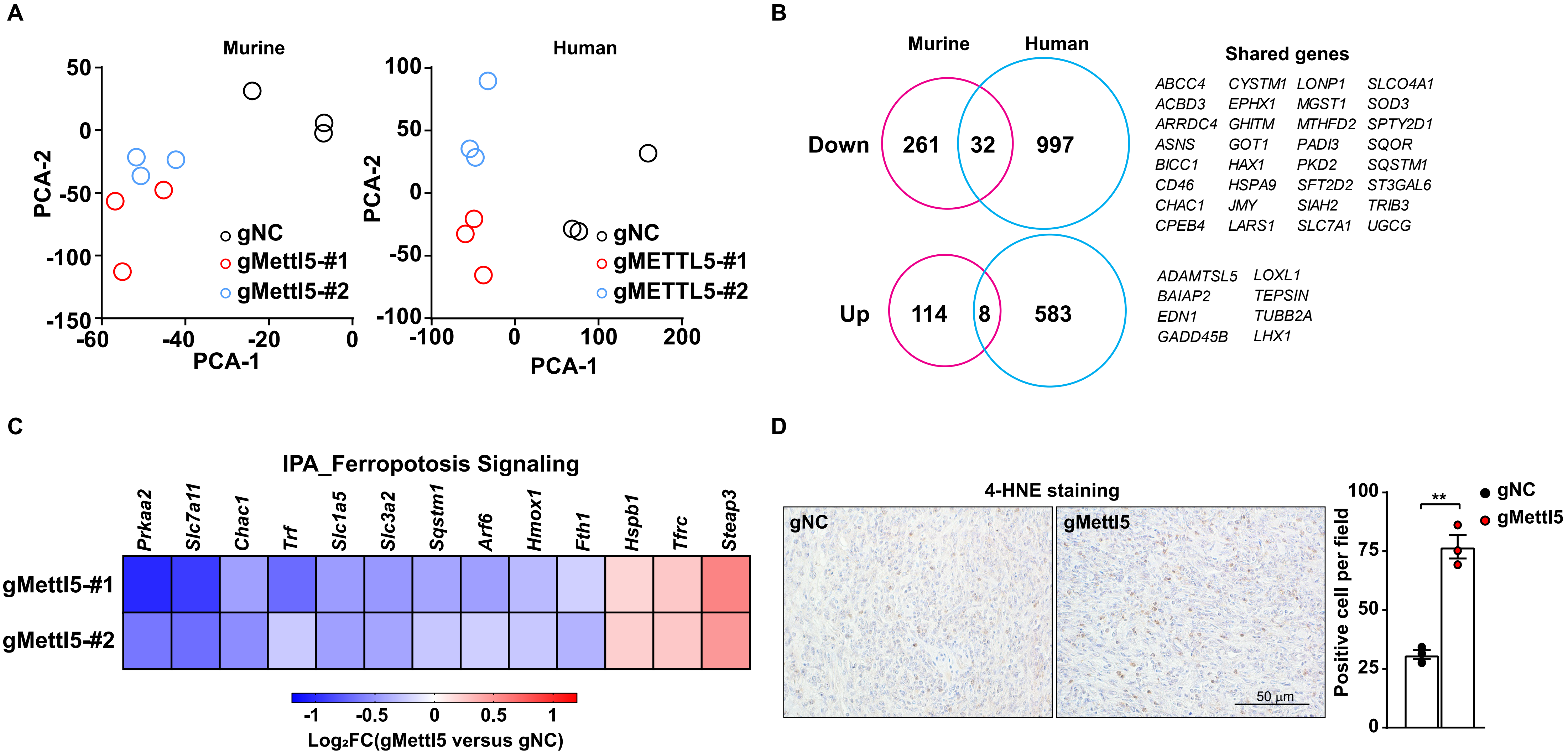

Supplementary Figure S7. ***METTL5*** **knockout alters lipid peroxidation and ferroptosis. (A)** Principal component analysis (PCA) was performed to reveal distinct transcriptomic profiles for *METTL5*-KO and control ID8/GC (left) and SKOV3/C (right) cells. **(B)** Venn diagrams illustrating the overlap of DEGs between murine and human OCs. DEGs were identified with a cutoff of $|Log_2(\text{Fold Change})| > 0.25$ and $p < 0.05$. Numbers of significantly downregulated (upper) and upregulated (lower) DEGs for ID8/GC (red) and SKOV3/C (blue) cell lines are indicated. **(C)** Murine DEGs involved in the "IPA_Ferroptosis signaling pathway" were identified. The relative Log_2FCs of selected genes in *Mettl5*-KO cells were illustrated. **(D)** Immunohistochemical staining of 4-HNE in tumor tissues. On day 13, tumor tissues were collected from C57BL/6 mice bearing modified ID8/GC cells (n=3 per group), and stained by 4-HNE to assess lipid peroxidation. Positive cells were quantified from at least three independent microscopic fields per sample. Representative images from each group were shown. Data are presented as mean ± SEM and were analyzed using Student's t-test. $^{**}p < 0.01$.

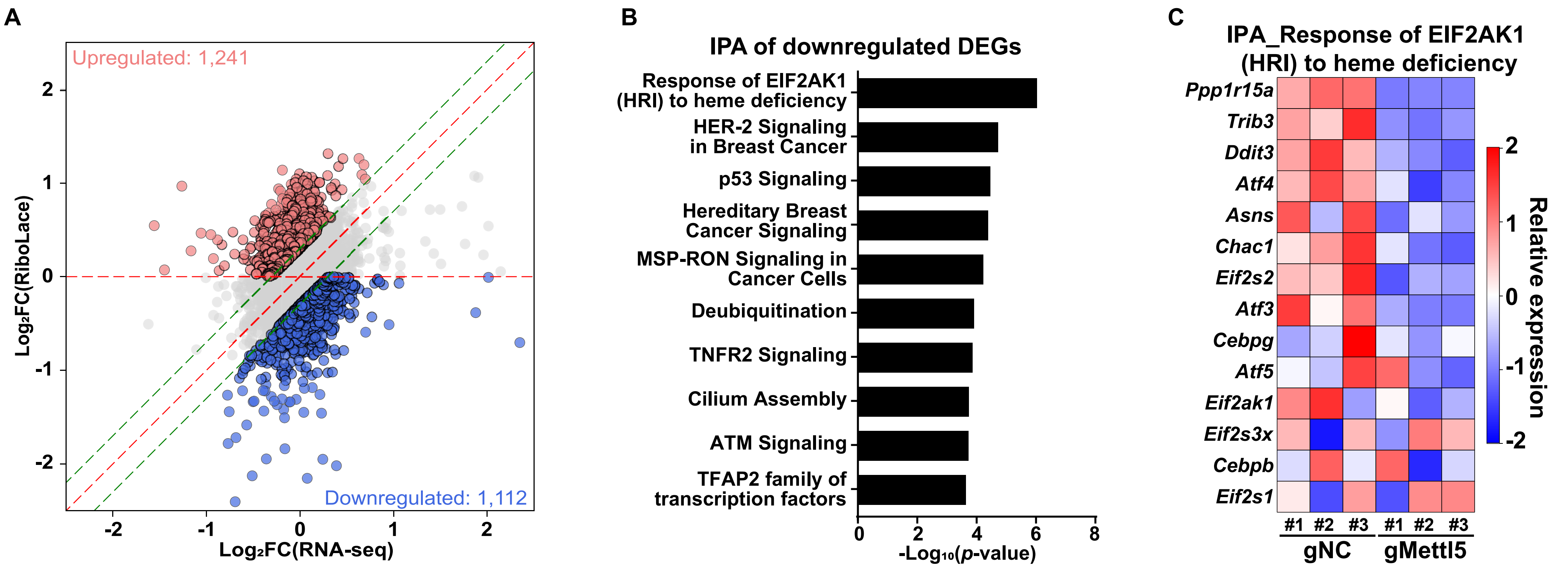
A
Upregulated: 1,241
Downregulated: 1,112
Log₂FC(RiboLace)
Log₂FC(RNA-seq)
2
1
0
-1
-2
B
IPA of downregulated DEGs
Response of EIF2AK1 (HRI) to heme deficiency
HER-2 Signaling in Breast Cancer
p53 Signaling
Hereditary Breast Cancer Signaling
MSP-RON Signaling in Cancer Cells
Deubiquitination
TNFR2 Signaling
Cilium Assembly
ATM Signaling
TFAP2 family of transcription factors
0
2
4
6
8
-Log₁₀(p-value)
C
IPA_Response of EIF2AK1 (HRI) to heme deficiency
Ppp1r15a
Trib3
Ddit3
Atf4
Asns
Chac1
Eif2s2
Atf3
Cebpg
Atf5
Eif2ak1
Eif2s3x
Cebpb
Eif2s1
#1 #2 #3 #1 #2 #3
gNC
gMettl5
Relative expression
2
1
0
-1
-2

Supplementary Figure S8. Integration of results from RNAseq and RiboLace analyses. (A) Correlations between transcriptional changes and those in active ribosome profiles of *Mettl5*-KO ID8/GC cells. DEGs were identified based on translation efficiency (|Log_2FC (RiboLace/RNA-seq)|) > 0.3 and |Log_2_RiboLace| > 0.3. **(B)** Ingenuity Pathway Analysis of downregulated DEGs in *Mettl5*-KO ID8/GC cells. The top 10 enriched pathways with statistical significance of downregulated DEGs ($p < 0.05$) were illustrated. **(C)** Expression levels of genes in the "IPA_Response to EIF2AK1 (HRI) to heme deficiency pathway" in *Mettl5*-KO and control ID8/GC cells. Normalized relative expression levels derived from RiboLace results were illustrated.

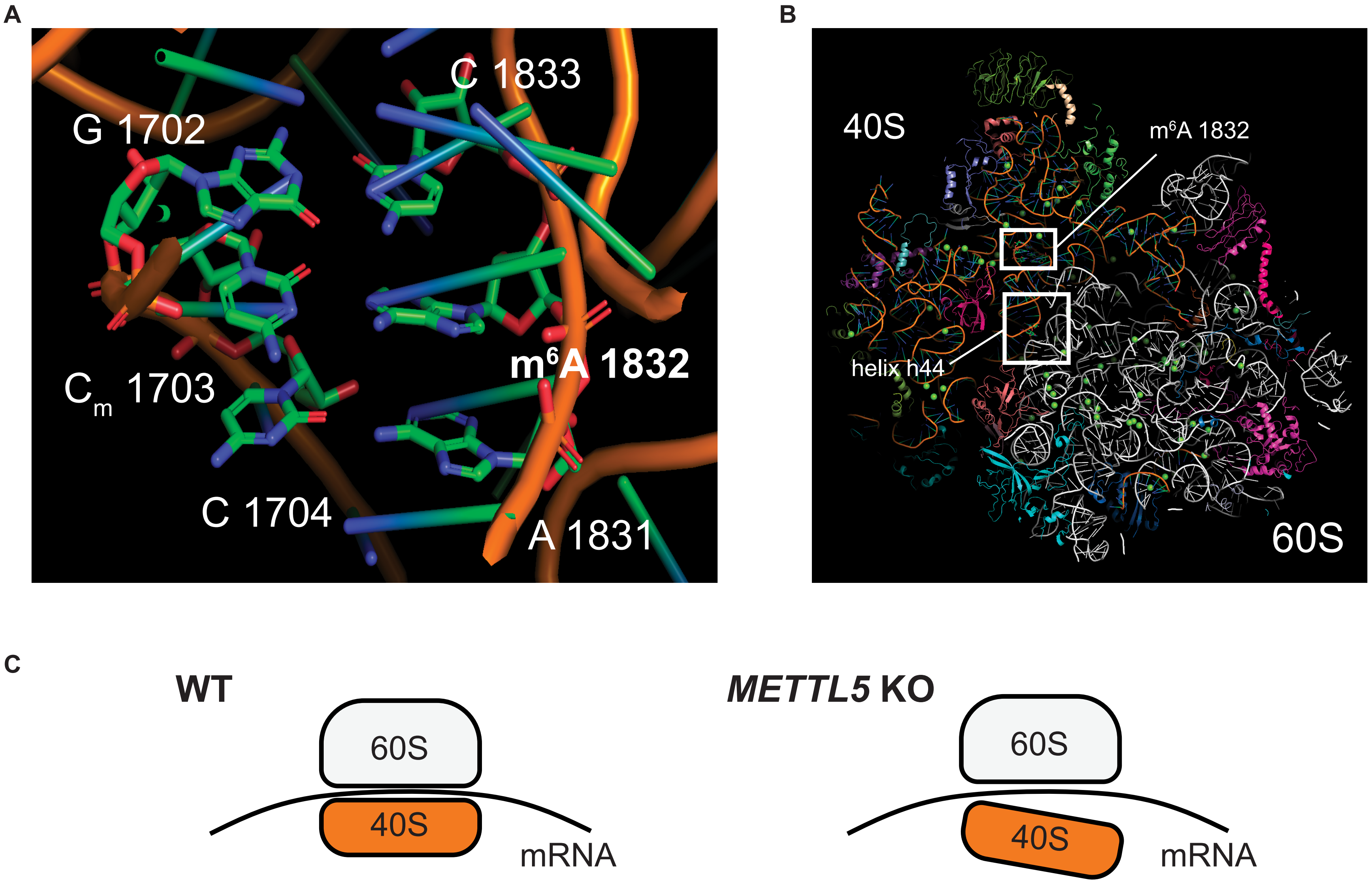
A
C 1833
G 1702
m6A 1832
Cm 1703
C 1704
A 1831
B
40S
m6A 1832
helix h44
60S
C
WT
60S
40S
mRNA
METTL5 KO
60S
40S
mRNA

Supplementary Figure S9. Impacts of m^6A1832 on ribosome assembly and scanning. (A) Cryo-EM structure illustrating the local environment of m^6A1832, surrounded by neighboring nucleotides. Altering m^6A1832 to adenine disrupts base interactions in this vicinity. **(B)** Cryo-EM structure of human 80S ribosome. m^6A1832 is physically close to helix 44 (h44) of the 18S rRNA, highlighted in white. Cryo-EM figures were visualized using PyMOL **(C)** Schematic representation of aberrant ribosome assembly upon *METTL5* depletion. The absence of *METTL5* results in abnormal assembly of the 40S ribosomal subunit. This disruption in assembly impairs ribosome scanning.

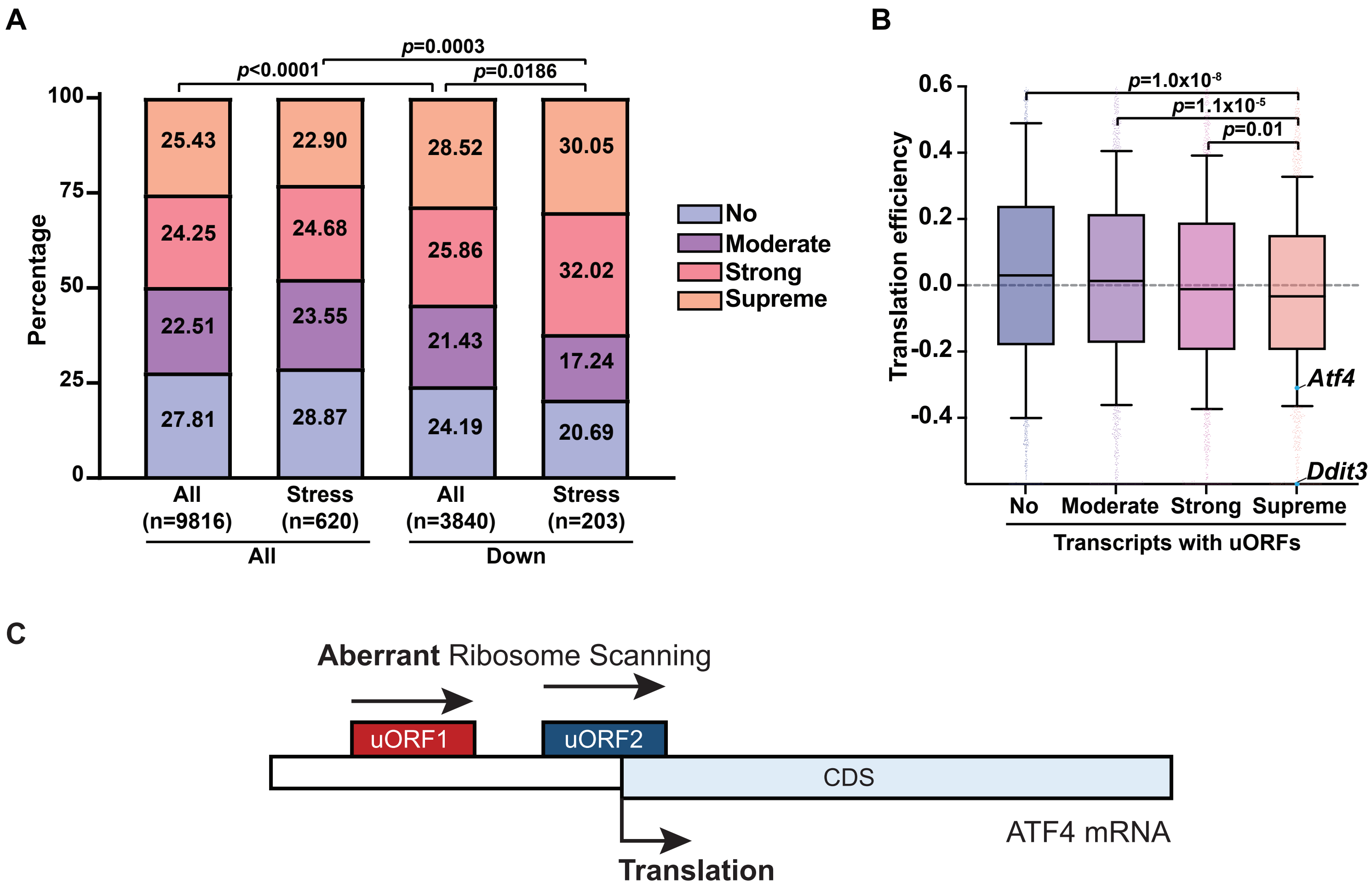
A
p=0.0003
p<0.0001
p=0.0186
Percentage
100
75
50
25
0
25.43
24.25
22.51
27.81
22.90
24.68
23.55
28.87
28.52
25.86
21.43
24.19
30.05
32.02
17.24
20.69
All (n=9816)
Stress (n=620)
All (n=3840)
Stress (n=203)
All
Down
No
Moderate
Strong
Supreme
B
p=1.0x10-8
p=1.1x10-5
p=0.01
Translation efficiency
0.6
0.4
0.2
0.0
-0.2
-0.4
Atf4
Ddit3
No
Moderate
Strong
Supreme
Transcripts with uORFs
C
Aberrant Ribosome Scanning
uORF1
uORF2
CDS
ATF4 mRNA
Translation

Supplementary Figure S10. METTL5 negatively regulates the translation of genes involved in cell stress responses. (A) Percentages of transcripts with varying Kozak strengths in RiboLace results. Transcripts sequenced by RiboLace were categorized into four groups based on their corresponding Kozak strengths. The four groups include 'No' (no or only weak Kozak sequence), 'Moderate' (at least one moderate Kozak sequence), 'Strong' (one strong Kozak sequence), and 'Supreme' (multiple strong Kozak sequences). Percentages of these categories were compared among all detected transcripts and genes listed in the GO pathway “Cellular responses to stress” (GO: 0033554), labeled as “Stress”. Exact *p*-values calculated by chi-squared test were shown. **(B)** Boxplot illustrating changes of four categories of transcripts in *Mettl5*-KO cells. Translation efficiency of genes with greater Kozak strength, including *Atf4* and *Ddit3,* significantly decreased after *Mettl5* knockout. Exact *p*-values with statistical significance were shown. **(C)** Overview of *ATF4* mRNA translation process. The translation of *ATF4* mRNA involves two key upstream open reading frames (uORFs). uORF1 is a positive-acting element that facilitates ribosome scanning and re-initiation at downstream coding regions in the *ATF4* mRNA, while uORF2 is an inhibitory element that blocks ATF4 expression. The absence of the m^6A modification in 18S rRNA triggers aberrant ribosome scanning, which is responsible for the unique response observed in *ATF4* mRNA translation.

Supplementary Figure S11

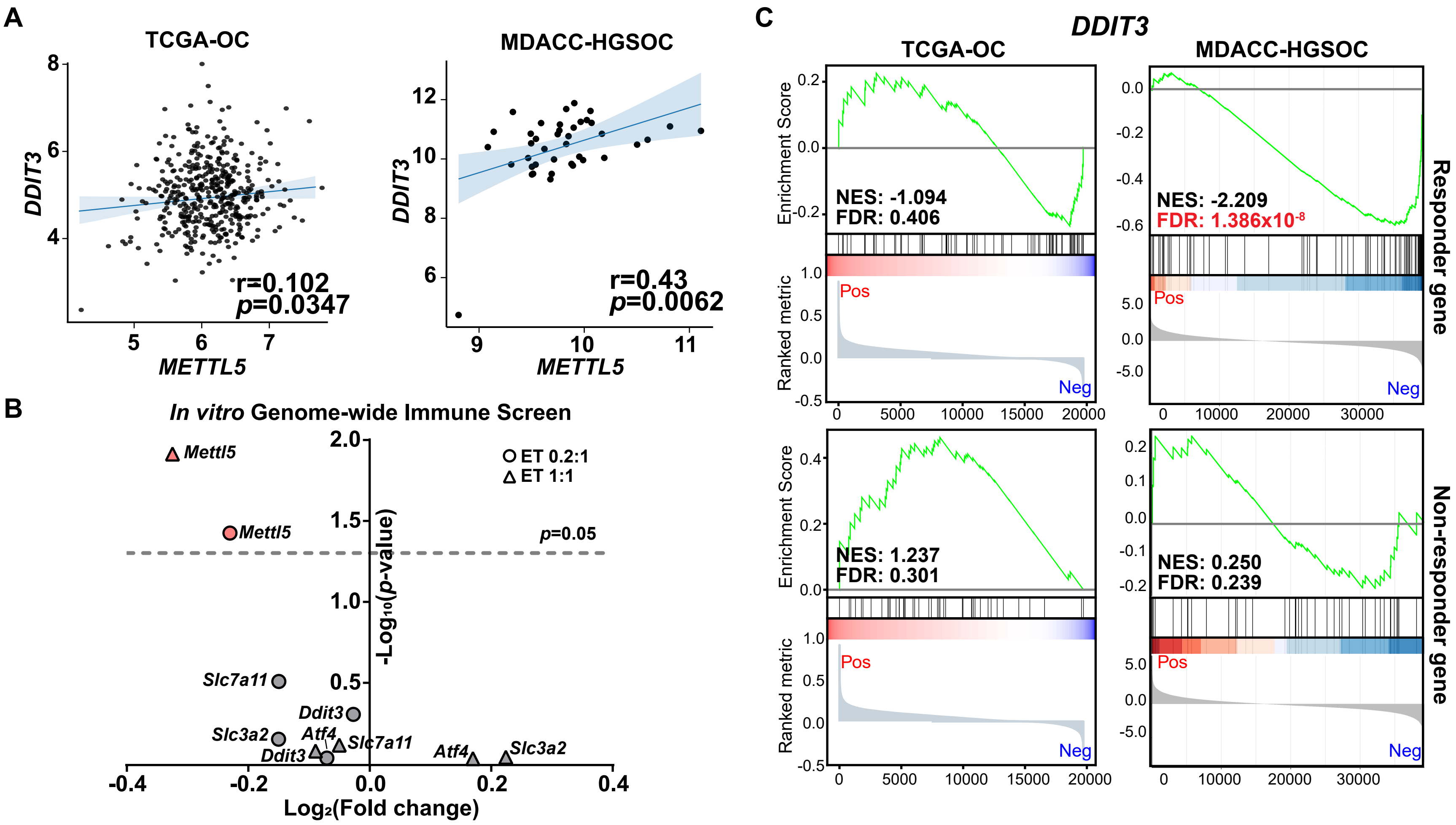

Supplementary Figure S11. Correlations of *DDIT3* expression and antitumor immune responses. (A) Analysis of the relationships between *METTL5* and *DDIT3* in the TCGA-OC and MDACC-HGSOC cohorts. The expression levels, calculated as Log_2(TPM+1) expression values were used for Pearson correlation analysis. **(B)** Effects of gene-specific KO on T cell mediated antitumor immune response. Relevant results of *in vitro* CRISPR immune screens were extracted. The Log_2 fold change of the second-best gRNA for each gene was selected for data representation. p-values were calculated using a negative binomial model. Grey dashed line indicated $p = 0.05$. **(C)** Gene set enrichment analysis (GSEA) of ICB response-associated gene sets in OC samples with differential *DDIT3* expressions. Transcriptomic data from baseline triple-negative breast cancer (TNBC) tumors in the TONIC cohort were used to identify differentially expressed genes (DEGs) between responders and non-responders. The top 100 upregulated genes in responders and non-responders formed the responder and non-responder gene sets, respectively. Patients in the TCGA-OC cohort (Left) and MDACC-HGSOC cohort (Right) were stratified into *DDIT3*-High and *DDIT3*-Low groups based on *DDIT3* expression in baseline tumor samples. Gene expression differences between these groups were analyzed, and GSEA was conducted to assess the association of *DDIT3* with ICB responses. Normalized enrichment scores (NES) and false discovery rates (FDR) were calculated, with statistically significant FDR values (FDR < 0.05) highlighted in red.

Supplementary Table S1. List of depleted genes identified *in vitro* genome-wide immune screens at an ET ratio of 0.2:1. The top 50 genes with gRNAs significantly depleted in the T cell-treated group at an ET ratio of 0.2:1 are listed and ranked according to their corresponding MAGeCK scores. Genes depleted under both ET ratio conditions of 0.2:1 and 1:1 are highlighted in yellow.

Supplementary Table S2. List of enriched genes identified in *in vitro* genome-wide immune screens at an ET ratio of 0.2:1. The top 50 genes with gRNAs significantly enriched in the T cell-treated group at an ET ratio of 0.2:1 are listed and ranked according to their corresponding MAGeCK scores. Genes enriched under both ET ratio conditions of 0.2:1 and 1:1 are highlighted in yellow.

Supplementary Table S3. List of publicly available ICB cohorts used for reported analysis.

Supplementary Table S4. List of gene-of-interest included in the ICB sub-library for *in vivo* ICB screen.

Supplementary Table S5. List of top 50 candidates from *in vivo* ICB screens based on the total score.

Supplementary Table S6. List of the responder gene set and non-responder gene set generated from the TONIC cohort.

Supplementary Table S7. The top 15 canonical pathways identified in Ingenuity Pathway Analysis (IPA). Top 15 canonical pathways enriched in differentially expressed genes (DEGs) were ranked by statistical significance (*p*-values). The analysis compares murine and human ovarian cancer cell lines with and without *METTL5* knockout.

Supplementary Table S8. Protospacer sequences of *METTL5*-specific gRNAs.

Supplementary Table S9. List of primers used in reported assays.

Gene symbol	Neg|LFC	Neg|*p* -value
Rnf31	-0.8827	2.69E-07
Cflar	-1.3087	2.69E-07
Traf2	-0.53765	2.69E-07
Map3k7	-0.80389	2.69E-07
Rela	-0.56255	2.69E-07
Fadd	-0.79195	2.69E-07
Rbck1	-0.64824	8.07E-07
Ikbkg	-0.94129	8.07E-07
Casp8	-0.49856	5.65E-06
Irgm1	-0.34216	1.48E-05
Ntn4	-0.33711	0.00014606
Zfp644	-0.4033	0.00017404
Ints6	-1.0211	0.0001813
Runx2	-0.35988	0.00018372
Copz2	-0.35118	0.00026388
Atraid	-0.37821	0.00027787
Arel1	-0.31942	0.00029939
Satb2	-0.38389	0.00046939
Tbc1d9b	-0.477	0.00049145
Dcbld2	-0.50361	0.00049413
Traf3	-0.43443	0.00054901
Cux1	-0.27792	0.00057752
Mprip	-0.48699	0.00060872
Ctdsp1	-0.39655	0.00063024
Zfp568	-0.3878	0.00065607
Fmo6	-0.48431	0.00077926
Nfxl1	-0.40807	0.00083091
Ppp2r2a	-0.37596	0.00083414
Acbd5	-0.27261	0.00090623
Fam213a	-0.46079	0.00093743
Ist1	-0.63806	0.00095895
Osmr	-0.35175	0.0010025
Irf2bp1	-0.44108	0.0010095
Lat	-0.4164	0.00083898
Akap7	-0.48533	0.0011096
Tapbp	-0.3804	0.0011284
Cdc42ep1	-0.33375	0.0013059
Manf	-0.45546	0.0013275
Rabl5	-0.45283	0.0013931
Smarca4	-0.53067	0.0014135
Wfdc13	-0.29927	0.0014415
Mospd2	-0.35389	0.0014496
Vmn1r213	-0.43536	0.0015922
Slc26a1	-0.42358	0.0018488
Ctsg	-0.5692	0.0019166
Ubtd2	-0.40471	0.0020984
Mtmr9	-0.35127	0.0021135
Calr	-0.29451	0.002164
Pex3	-0.29924	0.0022205
Prkcdbp	-0.34617	0.0023808

Gene symbol	Pos|LFC	Pos|*p* -value
Gm6531	0.81477	4.03E-06
Irf2	0.34548	2.18E-05
Jak2	0.47582	4.06E-05
B2m	0.37457	4.49E-05
Ifngr1	0.69832	4.55E-05
Socs3	0.51697	7.88E-05
Snrpa1	0.77383	0.00012831
Slc39a1	0.44141	0.00019018
Nek2	0.2759	0.00021223
Orc1	1.271	0.00027948
Wdr93	0.66264	0.00028056
Lurap1l	0.47816	0.00032091
Matn1	0.28323	0.00037148
Rad54l	0.60271	0.00044787
Fam49b	0.3617	0.0006141
Psmc3	0.59532	0.00063778
1700012P22Rik	0.41272	0.00088202
Arl5a	0.64328	0.00092667
Sdhaf2	0.56683	0.001037
Gpr101	0.43038	0.0010843
Gm14124	0.50307	0.00089923
Zfp109	0.30048	0.0011429
Rftn1	0.57226	0.0013458
Pgd	0.60229	0.0013544
Nat8	0.38098	0.0014334
Wars	0.7478	0.0015297
Nt5c3b	0.28799	0.0016416
Akr1b10	0.41231	0.001717
Tomm6	0.53647	0.0017245
Fibp	0.34864	0.0018434
Brs3	0.39694	0.0019149
Phf10	0.44015	0.0019311
Sil1	0.32602	0.002065
Plekha8	0.6661	0.0021796
Dmrt2	0.32363	0.0022297
Kmt2a	0.47582	0.0022302
Cry1	0.5153	0.002348
Prph2	0.32541	0.0025083
Kdm8	0.53947	0.0026175
Col4a5	0.44998	0.0026305
Sh2d4a	0.35155	0.0027838
Gtf3c6	0.65317	0.0028473
Barx2	0.28826	0.0030732
Tnfrsf1b	0.30761	0.0031087
Adamts4	0.43528	0.0031335
Gpatch3	0.29471	0.0032394
Rps6ka4	0.33563	0.0033433
Homer1	0.38857	0.0033476
Actl10	0.3947	0.0033734
Podnl1	0.27222	0.0036263

#	Study Desgination	PMID	Clinical trial Number	Cancer type	Clinical benefit assessment tool	Responders	Non-Responders
1	**SKCM-Riaz**	29033130	NCT01621490	Melanoma	RECIST v1.1	10	41
2	**SKCM-Gide (Mono)**	30753825	N/A	Melanoma	RECIST v1.1	33	21
3	**SKCM-Hugo**	26997480	N/A	Melanoma	RECIST v1.1	15	12
4	**SKCM-Gide (Dual)**	30753825	N/A	Melanoma	RECIST v1.1	38	13
5	**TNBC-TONIC**	31086347	NCT02499367	triple negative breast cancer	RECIST v1.1	10	43
6	**KIRC-CheckMate025**	26406148	NCT01668784	renal cell carcinoma	RECIST v1.1	7	36
7	**KIRC-Ascierto**	27491898	NCT01354431	renal cell carcinoma	RECIST v1.1	4	7
8	**KIRC-Miao Archival**	29301960	N/A	renal cell carcinoma	RECIST v1.1	3	13
9	**KIRC-Miao NCT01358721**	27169994	NCT01358721	renal cell carcinoma	RECIST v1.1	5	12
10	**KIRC-IMmotion150**	29867230	NCT01984242	renal cell carcinoma	RECIST v1.1	11	25
11	**KIRC-CheckMate010**	33657295	NCT03141177	renal cell carcinoma	RECIST v1.1	8	14
12	**BLCA-IMvigor210**	27939400	NCT02108652	Bladder Cancer	RECIST v1.1	34	133
13	**BLCA-Snyder**	28552987	NCT02951767	Bladder Cancer	RECIST v1.1	11	14
14	**STAD-Kim**	30013197	NCT02589496	Gastric Adenocarcinoma	RECIST v1.1	12	33
15	**GBM-Cloughesy**	30742122	NCT04201873	Glioblastoma Multiforme	RANO	NA	NA
16	**GBM-Zhao**	30742119	Trial 22981/26981 and CE.3	Glioblastoma Multiforme	RANO	9	8

Target													
1700017B05Rik	B3gat3	Ceacam2	Dgkq	Flrt3	Hsp90b1	Lactb	Mill2	Nup153	Prkdc	Rela	Slc17a5	Tango2	Ttc27
2900026A02Rik	Bag4	Cenpm	Dhcr7	Fosl2	Hspa1b	Lad1	Mixl1	Nup35	Prmt1	Rer1	Slc25a10	Tap1	Tuba1c
4933434E20Rik	BC049352	Cflar	Dhrs7	Foxk2	Hspa5	Lamp2	Mlh1	Nup85	Prmt5	Rest	Slc25a39	Tapbp	Txlng
9430015G10Rik	Bcap31	Chd8	Dhtkd1	Foxl2	Hspe1	Lamtor5	Mlst8	Ocln	Prmt7	Rfc2	Slc26a1	Tax1bp1	Txndc12
A430005L14Rik	Birc2	Chic2	Dkk4	Frmd8	Htra4	Laptm4a	Mlx	Osbp	Prpf40a	Rfc3	Slc30a9	Tbc1d9b	Tymp
Abcd1	Bloc1s1	Chit1	Dnajb8	Fsip1	Htt	Larp4b	Mmd2	Osgep	Prpf6	Rfx1	Slc6a15	Tbk1	Ubald2
Abcg3	Brat1	Chmp3	Dnase2a	Ftmt	Hus1	Lce1e	Mmp11	Osmr	Prph2	Rhag	Slco1b2	Tbr1	Ubb
Adad1	Brca1	Chmp5	Dnase2b	Gadd45g	Icos	Lelp1	Morf4l2	Oxct1	Prr11	Rhebl1	Slk	Tcea3	Ubd
Adam29	Brca2	Chmp6	Dnmt1	Gata3	Ido1	Lenep	Mos	Oxsr1	Prrc2c	Rmi1	Smad2	Tcf12	Ube2m
Adar	Brk1	Chrm4	Dock7	Gca	Iffo2	Leo1	Mphosph10	P2rx1	Psg29	Rnaseh1	Smarca4	Tctex1d1	Ubtd2
Adrm1	Brms1l	Clic6	Dok7	Gdap1	Ifnar2	Lgalsl	Mprip	Pabpc1	Psma2	Rnaseh2a	Smc2	Ten1	Ubxn1
Afg3l2	Btn1a1	Clspn	Dolpp1	Get4	Ifngr1	Lgr5	Mrgbp	Padi3	Psma5	Rnaseh2b	Smg5	Tenm4	Ubxn7
Agap1	Btrc	Cnn3	Dscc1	Gfpt1	Ifngr2	Lhfpl2	Mrpl3	Pagr1a	Psma7	Rnaseh2c	Smg7	Tfdp2	Ugdh
Aif1l	C1ql4	Cnot3	Dut	Gins1	Ifrd1	Lin54	Mrpl32	Parp4	Psmb3	Rnf168	Smim18	Tfpi2	Uggt2
Akap1	C4b	Coil	Dync1li1	Gk5	Ikbip	Litaf	Mrpl55	Pcbp1	Psmd1	Rnf31	Smim19	Tgfbrap1	Usp37
Akna	Cad	Col4a1	E2f1	Gla	Ikbkg	Lmnb2	Mrps36	Pcgf1	Psmd2	Rnf40	Smoc1	Tgif1	Usp7
Akt1s1	Calhm2	Copa	Ebf2	Glb1l	Ikzf2	Lnx2	Msh2	Pcif1	Psmd8	Rnps1	Snf8	Thada	Uty
Aldoart1	Calr	Copb1	Ebpl	Glis1	Il17rd	Lonrf3	Msh3	Pdia3	Psmg4	Rpl26	Snx3	Thoc2	Vcp
Alg2	Calu	Cops5	Ece1	Glt1d1	Il18bp	Loxl2	Mta2	Pdia6	Psph	Rpl8	Socs3	Timm10b	Vmn1r238
Alg3	Camk1g	Copz2	Eef1d	Gm765	Il21	Lrp10	Mthfd1	Pex11a	Ptger3	Rpn2	Sowaha	Timm8a1	Vps13c
Alg5	Camkk2	Cox4i2	Eef2	Gmfb	Il5ra	Lrrc1	Mtmr9	Pex13	Ptpn23	Rps11	Sp100	Tlcd1	Vps25
Alg8	Camsap3	Cpa6	Eftud2	Gmnc	Imp4	Lrrc45	Mtnr1b	Pex5l	Qk	Rps19bp1	Spata2	Tm9sf3	Vps26b
Amer2	Capza1	Cpvl	Eif2ak3	Gnpnat1	Ints12	Lrrc8e	Myo9b	Pfdn2	R3hcc1l	Rps2	Spata31d1d	Tm9sf4	Vps28
Ankrd11	Capzb	Crip2	Eif3b	Gorasp2	Ints2	Lsm7	Mzb1	Pgap3	Rab11b	Rpusd2	Spns1	Tmed3	Vps33a
Ankrd16	Car14	Csf2	Eif4a3	Gpaa1	Ints6	Lypd2	Mzf1	Pgpep1	Rab27a	Rtn2	Sptssa	Tmem147	Vps37a
Apobec3	Card14	Csf3	Eif4g2	Gpn2	Ints7	Mafk	N4bp1	Phrf1	Rab27b	Ruvbl1	Sri	Tmem173	Vps39
Aqr	Casp8	Csk	Eif6	Gpr143	Ints9	Map2k2	Naa40	Pigs	Rab2a	Samhd1	Srp9	Tmem184a	Wdr1
Arcn1	Cbfa2t2	Csnk1d	Elac1	Gpr158	Irf1	Map3k7	Nae1	Pigu	Rab3gap2	Scamp2	Srsf1	Tmem191c	Wdr45b
Arel1	Cbx4	Csnk2b	Ell	Gprin1	Irf2	Map6	Napa	Pisd	Rab43	Scamp3	Srsf6	Tmem42	Wee1
Arfip2	Ccdc25	Cstf1	Emc1	Gpx6	Irf3	Mapkapk2	Ncl	Pla2g12a	Rab6b	Scimp	Ssu72	Tmprss13	Wfdc10
Arhgap19	Ccl11	Cstl1	Endov	Grb7	Irf4	Mark2	Ncor1	Pla2g4e	Rabggtb	Scnn1b	St8sia6	Tmx2	Wif1
Armcx6	Ccl3	Ctdp1	Ercc1	Grhl2	Irf5	Mb21d1	Ndufv3	Plcl2	Rabif	Sdc1	Stat1	Tnfaip6	Wnt2b
Arpc1a	Ccl4	Cttn	Ercc2	Grip1	Irf6	Mbd3l2	Nelfcd	Pld2	Rad1	Sec23b	Stat3	Tnfrsf1b	Wrnip1
Asap3	Ccl5	Cul1	Ergic3	Gripap1	Irgc1	Mbtd1	Nfkbib	Plekhj1	Rad17	Secisbp2l	Stip1	Tnip2	Xpo1
Asb18	Ccl7	Cul3	Ermap	Gstcd	Irx5	Mcm7	Nfxl1	Plk1	Rad21	Serinc1	Strap	Top2a	Yes1
Asic5	Ccna2	Cux1	Erp27	Gtf2a2	Ist1	Med12	Nipal2	Pmpcb	Rad50	Serinc2	Stub1	Topbp1	Ywhaz
Ate1	Ccpg1	Cux2	Exosc10	Gtf2h3	Iws1	Med13	Nit1	Pms1	Rad51	Serpinb9	Styxl1	Tpcn1	Zcchc2
Atf4	Cct4	Cxcl13	Ext1	Gtpbp4	Jak2	Med14	Nit2	Podxl2	Rad51c	Sestd1	Suclg1	Tpd52l1	Zfat
Atf6	Cd109	Cxcl9	F8a	Gusb	Jakmip1	Med15	Nme1	Polr1c	Rad51d	Sf3a3	Sulf1	Traf2	Zfp185
Atg13	Cd151	Cyb5r1	Fadd	Hadhb	Jtb	Med20	Nmrk1	Pou4f3	Rad9a	Sf3b1	Sumo3	Traf3	Zfp384
Atg5	Cd274	Cyc1	Faim	Haus1	Katna1	Med30	Nol10	Ppp1cc	Rad9b	Sft2d2	Sun1	Traf7	Zfp647
Atp2c1	Cd2ap	D10Wsu102e	Fam160b1	Hdac3	Kdelr2	Med8	Nop58	Ppp1r15b	Raf1	Sgms1	Supt16	Trappc4	Zfx
Atp4a	Cd38	D3Ertd751e	Fam219a	Hoxb1	Kdm5c	Megf6	Npr2	Ppp1r16b	Rala	Sharpin	Supt20	Trex1	Zyg11b
Atp6ap2	Cd40	Dbr1	Fam89b	Hoxc10	Kdm8	Mepce	Nps	Ppp1r2	Ralgapa2	Shd	Supt4a	Trmt1l	
Atp6v0e	Cd47	Ddb1	Fasl	Hp	Kif11	Mettl5	Nr4a3	Ppp2cb	Ranbp9	Shisa5	Supt6	Trpm5	
Atraid	Cd74	Ddi2	Fat1	Hpse2	Kif4	Mettl7a2	Ntn3	Ppp2r2a	Rangap1	Sil1	Swsap1	Trpv1	
Aurka	Cd99l2	Ddit3	Fgf3	Hrct1	Kmt2a	Mfsd5	Ntn4	Prelid1	Rasl11b	Sim1	Sypl	Trpv5	
Aurkb	Cdc42ep1	Ddx21	Fgf4	Hsd17b14	Kmt2b	Mgll	Nubp2	Prkaa1	Rbck1	Sin3a	Syt17	Tsfm	
AW209491	Cdx2	Ddx3y	Fjx1	Hsp90aa1	Kpnb1	Micall2	Nudt12	Prkab1	Rbms3	Sipa1l1	Szt2	Tsr2	
B2m	Cdyl	Defb29	Flrt2	Hsp90ab1	Krt79	Mier2	Nudt5	Prkcz	Reg4	Slc16a9	Taf5l	Ttc1	

Gene	Total Score	Patient Score	*In vitro* score	Merged Score
Stub1	-6.456045243	-0.857142593	-3	-3.857142593
A430005L14Rik	-5.808713028	-1.304590492	-2	-3.304590492
Mettl5	-5.655458828	-2.447611636	-1	-3.447611636
Agap1	-5.498253309	-2.290460583	-1	-3.290460583
Coil	-5.18909127	-3.257708752	0	-3.257708752
Foxk2	-4.941183715	-0.942495271	-3	-3.942495271
Lypd2	-4.91124713	-1.316274206	-2	-3.316274206
Birc2	-4.892645472	0.983326194	3	3.983326194
Ppp2r2a	-4.82172488	0.587683956	3	3.587683956
Cpvl	-4.793668188	-1.580614018	-3	-4.580614018
Tbc1d9b	-4.781089519	-1.871972345	-2	-3.871972345
Nit1	-4.573027353	-1.958259813	-3	-4.958259813
Rbck1	-4.553840422	-0.882372263	-3	-3.882372263
Pcbp1	-4.510851574	-1.2012609	-2	-3.2012609
Padi3	-4.510504364	-2.415241235	-1	-3.415241235
AW209491	-4.498395793	1.11072801	2	3.11072801
D3Ertd751e	-4.412123416	-1.353014952	-2	-3.353014952
Lamp2	-4.287211984	-1.453464728	0	-1.453464728
Ist1	-4.247786768	-1.569059615	0	-1.569059615
Ebpl	-4.227610365	-3.256700864	0	-3.256700864
Hadhb	-4.219830663	-1.566919634	-2	-3.566919634
Lrp10	-4.15560048	-1.772362469	-2	-3.772362469
Pdia3	-4.140004465	2.539355954	0	2.539355954
Pla2g12a	-4.135823212	-2.168618764	-2	-4.168618764
Sharpin	-4.134382694	-0.645182859	-3	-3.645182859
Ppp2cb	-4.122923423	1.963539387	2	3.963539387
Slk	-4.120900832	-1.369185014	-2	-3.369185014
Gorasp2	-4.116035802	-2.878427047	-1	-3.878427047
Scnn1b	-4.10950627	0.229145062	3	3.229145062
Suclg1	-4.06244649	-3.28726845	0	-3.28726845
Spns1	-4.041588467	-1.817268554	-2	-3.817268554
Hp	-4.037667632	-1.798159801	-2	-3.798159801
Tlcd1	-4.01194665	2.435106078	0	2.435106078
Hspa5	-3.997068732	2.438902657	0	2.438902657
Mrgbp	-3.968254882	-1.523793049	-2	-3.523793049
Fadd	-3.960615948	-1.247868211	0	-1.247868211
Il17rd	-3.87472514	-2.215495606	0	-2.215495606
Bcap31	-3.871755117	-0.48720036	-3	-3.48720036
Zfp647	-3.853094255	-0.758308572	-3	-3.758308572
Ttc1	-3.85145613	-1.796910212	-2	-3.796910212
Frmd8	-3.789622073	0.42120723	3	3.42120723
N4bp1	-3.789508447	-1.47748909	-2	-3.47748909
Ubtd2	-3.74159933	-2.376811752	-2	-4.376811752
Psma5	-3.729589843	-2.686208851	-2	-4.686208851
Rnps1	-3.727129759	-1.460492749	0	-1.460492749
Sdc1	-3.707155121	-1.289853859	0	-1.289853859
Rnf40	-3.699156551	-1.765460897	-2	-3.765460897
Tmem147	-3.676542634	-0.350227971	4	3.649772029
Serinc1	-3.673201715	-1.931322599	-3	-4.931322599
Styxl1	-3.668879989	-0.691245154	-3	-3.691245154

Non-Responder gene set			
CNIH4	*ENST00000652729*	*AC117498.3*	*DAO*
AC046134.1	*ENST00000673523*	*JMJD4*	*ENST00000642232*
AC090958.2	*AC004898.1*	*SRP68P3*	*ENST00000649389*
UAP1	*ENST00000648334*	*CKS1BP7*	*ENST00000646359*
ENST00000643951	*CCZ1*	*HIST1H2BM*	*C1GALT1*
AC090958.1	*ENST00000651565*	*PPIAP79*	*ENST00000642519*
Z98885.1	*ENST00000652053*	*STX3*	*AC108693.1*
AC024937.2	*TMUB1*	*ENST00000650907*	*HSD3BP4*
ENST00000650615	*CICP22*	*C20orf24*	*ENST00000645433*
PLPPR1	*RPL7P12*	*ENST00000649838*	*PTMAP3*
AC100827.2	*ENST00000674376*	*ENST00000643587*	*BUD31*
ERICH6B	*ENST00000648236*	*RPS12P31*	*AC093591.3*
HIST2H2AB	*AC009220.3*	*ENST00000645124*	*TMED10P2*
NSMCE2	*WDR45B*	*GGPS1*	*ITPKC*
ENST00000648420	*HSF1*	*AC073055.1*	*ENST00000645650*
ENST00000647554	*ENST00000665513*	*TAS2R2P*	*KRT18P16*
FRG2C	*MTND4P15*	*KRT18P8*	*NPM1P19*
RANP6	*ENST00000645667*	*NPTX1*	*TRIB3*
AL732414.1	*ENST00000642557*	*AC089987.1*	*TPT1P1*
RPL6P30	*GET4*	*PPIAP6*	*HECW2*
CHCHD2P8	*PUSL1*	*AC005077.2*	*ENST00000647134*
TPSP2	*AC130689.1*	*ZEB2P1*	*ENST00000651348*
GAPDHP61	*ENST00000645851*	*AC105250.1*	*AC136687.1*
ENST00000643137	*PLAC1*	*EEF1E1P1*	*ENST00000644693*
ENST00000646252	*SNRPGP14*	*PGRMC1*	*SMIM34A*

Responder gene set			
KCNAB2	*IGKV3D-20*	*ENST00000648405*	*ENST00000648061*
GADD45G	*CD27*	*ADA2*	*CHIT1*
ZAP70	*AC073111.5*	*IGHG1*	*LIME1*
GZMH	*IGHG3*	*CD177*	*POU2AF1*
IGHG4	*HVCN1*	*MICAL1*	*FGF14*
NOTCH1	*CCL18*	*FOXF1*	*NME5*
APOBEC3G	*LDLRAD2*	*DLG4*	*CDC14A*
IGLC1	*GZMB*	*HLA-DPB2*	*PNOC*
ENST00000648762	*CLEC2D*	*PHYHD1*	*TMEM91*
COL6A4P2	*MARCO*	*ENST00000647138*	*ENST00000673657*
GBP5	*ARHGAP9*	*IGHM*	*ACAP1*
MMAA	*SEPTIN1*	*BCL2A1*	*MZB1*
ENST00000649273	*RSPO3*	*MTHFD1*	*IGKV2D-29*
ENST00000650477	*AC234301.3*	*WDFY4*	*CNN1*
KCNN3	*ENST00000647932*	*RRN3P1*	*SLC38A5*
MEF2B	*FCGR1A*	*IGLV1-41*	*ENST00000651450*
REC8	*SLC17A9*	*ENST00000648395*	*IRF4*
CCL4	*MPZ*	*ADAMTS8*	*CD38*
PRF1	*ENST00000672386*	*CCBE1*	*CD247*
DPY19L2	*IGKC*	*DERL3*	*CD37*
ENST00000642990	*CCL3*	*ENST00000648117*	*C3orf62*
P2RX1	*ENST00000652640*	*ENST00000673253*	*COX7A1*
IGLC2	*WNT2B*	*TMEM232*	*SLAMF8*
C2orf88	*FAM19A5*	*MED13L*	*LILRB2*
TOGARAM2	*VILL*	*ENST00000644072*	*FMN2*

**Top15_Ingenuity Canonical Pathways_Murine**	**-log(p-value)**
Ferroptosis Signaling Pathway	6.45
LPS/IL-1 Mediated Inhibition of RXR Function	5.82
Xenobiotic Metabolism AHR Signaling Pathway	5.71
Aryl Hydrocarbon Receptor Signaling	4.8
Autophagy	4.74
NRF2-mediated Oxidative Stress Response	4.3
Sirtuin Signaling Pathway	3.86
Remodeling of Epithelial Adherens Junctions	3.85
Axonal Guidance Signaling	3.82
HIF1α Signaling	3.67
γ-linolenate Biosynthesis II (Animals)	3.61
IL-10 Signaling	3.55
Xenobiotic Metabolism CAR Signaling Pathway	3.4

**Top15_Ingenuity Canonical Pathways_Human**	**-log(p-value)**
Estrogen Receptor Signaling	12.2
Mitochondrial Dysfunction	11.3
Pulmonary Fibrosis Idiopathic Signaling Pathway	11.2
Myelination Signaling Pathway	10.7
Hepatic Fibrosis Signaling Pathway	10.6
Superpathway of Cholesterol Biosynthesis	10.4
Axonal Guidance Signaling	10.3
Osteoarthritis Pathway	9.75
Sirtuin Signaling Pathway	9.56
ILK Signaling	8.15
Tumor Microenvironment Pathway	7.9
Colorectal Cancer Metastasis Signaling	7.82
Ferroptosis Signaling Pathway	7.7
CLEAR Signaling Pathway	7.16

gRNA ID	protospacer sequences of gRNAs
NC	5'-CCGCAATATATGCGGTAAG
murine Mettl5_1#	5'-AGCGGCAATGCTAGGAGCA
murine Mettl5_2#	5'-GGAGTACTTAGCATCGGAG
Human Mettl5_1#	5'-AGAGAGTCGCCTGCAACAAG
Human Mettl5_2#	5'-CCGCCCTACCTGCAATGTG

Flanking sequences for gRNA construction		
Forward	5'-CACCGXXXXXXXXXXXXXXXXXXX	(X:19nt protospacer sequence)
Reverse	5'-AAACXXXXXXXXXXXXXXXXXXXC	(X:19nt reverse complement protospacer sequence)

Supplementary Table S9

	Sequence (5'-3')	Application	Description
**Amp_F**	5'-ATTTCTTGGCTTTATATATCTTGTGG	ICB sublibrary construction	Forward primer for oligo amplification for gRNA sublibrary construction
**Amp_R**	5'-TTAAACTTGCTATGCTGTTTCCA		Reverse primer for oligo amplification for gRNA sublibrary construction
**1st_F**	5'-CAGGAAGAGGGCCTATTTCCCATG	RNA-seq for detection of gRNA distribution-1st round PCR	Forward primer for 1st Round of PCR -RNAseq for detection of gRNA distribution
**1st_R**	5'-TGCCACTTTTTCAAGTTGATAACGGAC		Reverse primer for 1st Round of PCR -RNAseq for detection of gRNA distribution
**2nd-F-P5_D5XX**	5'-AATGATACGGCGACCACCGAGATCTACAC########ACACTCTTTCCCTACACGACGCTCTTCCGATCTTCTTGTGGAAAGGACGAAACACC	RNA-seq for detection of gRNA distribution-2nd round PCR for adding standard Illumina adapater and sample barcode	Forward primer for 2nd Round of PCR -RNAseq for detection of gRNA distribution-adding P5 adapter; ######### is 8bp sample index of Trueseq HT D5XX from Illumina
**2nd-R-P7_D7XX**	5'-CAAGCAGAAGACGGCATACGAGAT########GTGACTGGAGTTCAGACGTGTGCTCTTCCGATCCACTTTTTCAAGTTGATAACGGACTAGCC		Reverse primer for 2nd Round of PCR -RNAseq for detection of gRNA distribution-adding P7 adapter; ######### is 8bp sample index of Trueseq HT D7XX from Illumina
**FLAG-Atf4_F**	5'-GTGAGGATCTATTTCCGGTGATGGACTACAAAGACGATGAC	Vector construction for the overexpression of FLAG tagged Atf4	Forward primer for the plasmid construction with FLAG tagged Atf4
**FLAG-Atf4_R**	5'-GGGGGGAGGGAGAGGGGCGGTTACGGAACTCTCTTCTTC		Reverse primer for the plasmid construction with FLAG tagged Atf4
**Slc7a11_ChIP_F**	5'-TAAGCAACCGCCTGTCACAC	Atf4 Chromatin IP assay	Forward primer for detecting Atf4 binding site on the Slc7a11 promoter region
**Slc7a11_ChIP_R**	5'-GCTGAGTAATGTTGGCGCTTT		Reverse primer for detecting Atf4 binding site on the Slc7a11 promoter region
**Slc3a2_ChIP_F**	5'-CTCGCACACCGCTTACCTTA		Forward primer for detecting Atf4 binding site on the Slc3a2 promoter region
**Slc3a2_ChIP_R**	5'-CGACAAGAACAACGTGTGGC		Reverse primer for detecting Atf4 binding site on the Slc3a2 promoter region
**Negatvie_ChIP_R**	5'-ATTTTGTGCTGCATAACCTCCT		Forward primer of non-binding site served as the negative control for ChIP assay
**Negatvie_ChIP_F**	5'-TAGCAACATCCTAAGCTGGACA		Reverse primer of non-binding site served as the negative control for ChIP assay

Primer for qPCR	Species	Sequence
**SLC7A11_F**	Homo sapiens	5'-TCCTGCTTTGGCTCCATGAACG
**SLC7A11_R**	Homo sapiens	5'-AGAGGAGTGTGCTTGCGGACAT
**SLC3A2_F**	Homo sapiens	5'-CCAGAAGGATGATGTCGCTCAG
**SLC3A2_R**	Homo sapiens	5'-GAGTAAGGTCCAGAATGACACGG
**ACTB-F**	Homo sapiens	5'-CACCATTGGCAATGAGCGGTTC
**ACTB-R**	Homo sapiens	5'-AGGTCTTTGCGGATGTCCACGT
**GAPDH-F**	Homo sapiens	5'-GTCTCCTCTGACTTCAACAGCG
**GAPDH-R**	Homo sapiens	5'-ACCACCCTGTTGCTGTAGCCAA
**Slc7A11_F**	Mus musculus	5'-CTTTGTTGCCCTCTCCTGCTTC
**Slc7A11_R**	Mus musculus	5'-CAGAGGAGTGTGCTTGTGGACA
**Slc3A2_F**	Mus musculus	5'-GAGCGTACTGAATCCCTAGTCAC
**Slc3A2_R**	Mus musculus	5'-GCTGGTAGAGTCGGAGAAGATG
**Actb-F**	Mus musculus	5'-CATTGCTGACAGGATGCAGAAGG
**Actb-R**	Mus musculus	5'-TGCTGGAAGGTGGACAGTGAGG
**Gapdh-F**	Mus musculus	5'-CATCACTGCCACCCAGAAGACTG
**Gapdh-R**	Mus musculus	5'-ATGCCAGTGAGCTTCCCGTTCAG
**18sRNA-F**	Mus musculus	5'-CGCACGGCCGGTACAGTGAAACTG
**18sRNA-R**	Mus musculus	5'-CACCCGTGGTCACCATGGTAGGCA
